# Isolation of a Staudinger‐type Intermediate Utilizing a Five‐Membered Phosphorus‐Centered Biradicaloid

**DOI:** 10.1002/chem.202403893

**Published:** 2024-12-13

**Authors:** Y. Pilopp, J. Bresien, K. P. Lüdtke, A. Schulz

**Affiliations:** ^1^ Anorganische Chemie Institut für Chemie Universität Rostock A.-Einstein-Str. 3a 18059 Rostock; ^2^ Leibniz Institut für Katalyse e. V. Albert-Einstein-Str. 29a 18059 Rostock

**Keywords:** azides, biradical, Staudinger reaction, reaction mechanism, structure

## Abstract

The Staudinger reaction provides chemists with a valuable tool for the reduction of azides, which are notoriously unstable and can decompose explosively. By providing a controlled method for the conversion of azides to amines, the reaction opened up new avenues for the synthesis of various amine‐containing compounds that are widely used in natural products, pharmaceuticals and polymers. The Staudinger reaction begins with the nucleophilic attack of a trivalent phosphine (usually triphenylphosphine), leading to the formation of a triazenide intermediate, a highly reactive species. Here we report how a divalent phosphorus‐centered biradicaloid reacts with covalent azides and show that it is possible to capture and fully characterize the transient intermediate. The experimental data is supported by quantum chemical calculations of the reaction paths and in terms of thermodynamics and chemical bonding.

## Graphical abstract

**Trapping transient Staudinger‐type intermediates**. The reaction of five‐membered biradicaloids with azides (e. g. Dipp‐N_3_) leads to a variety of reaction pathways. Staudinger‐type intermediates can be isolated directly from the reaction mixture (without loss of N_2_) if specific time or temperature control is applied. SCXRD, low‐temperature NMR studies and calculations help to understand under which conditions addition or Staudinger type reaction takes place.

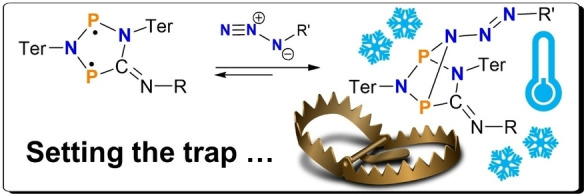

## Introduction

**Scheme 1:**
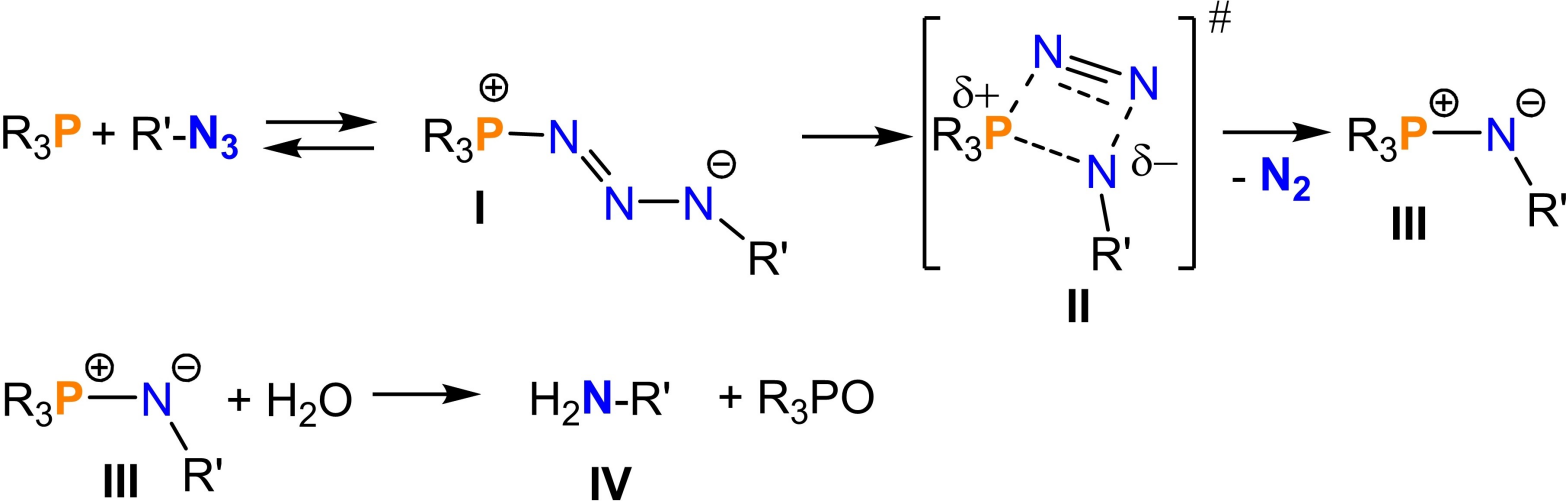
Mechanism of the Staudinger reaction (#=transition state).

The Staudinger reaction, named after the German chemist Hermann Staudinger, who discovered it in 1919, is a cornerstone in the field of organic chemistry.[^1^, ^2^, ^3^, ^4^, ^5^, ^6^, ^7^, ^8^] The reaction not only revolutionized synthetic organic chemistry by providing a direct method to produce amines from azides but also laid the groundwork for the subsequent discovery of the Staudinger ligation, a bioorthogonal reaction that has found widespread application in chemical biology.[^5^, ^8^, ^9^] From a mechanistic point of view, the Staudinger reaction can be divided into three steps (Scheme 1):^[10]^ (i) The azide addition to the trivalent phosphorus to form a triazenide (**I**), which (ii) rearranges in the transition state to form a PNNN four‐membered ring (**II**), which then decomposes to form an azaylide (**III**) with N_2_ elimination, whereby the phosphorus becomes formally pentavalent, i. e. it is an oxidation of the phosphorus from +III to +V. (iii) The subsequent hydrolysis of the azaylide, which is also known as an iminophosphorane, leads to the formation of the amine (**IV**).[^3^, ^11^] Due to the flat potential energy surface in the second step, the Staudinger reaction essentially requires one step to release N_2_, which means that Staudinger intermediates of type **I** are transient species and can only be isolated if they are stabilized by bulky substituents[^6^, ^10^, ^12^, ^13^, ^14^, ^15^] or by using an additional Lewis acid that forms an FLP assembly together with the R_3_P moiety.[^16^, ^17^, ^18^, ^19^, ^20^, ^21^, ^22^, ^23^] Metal cations can also be used as Lewis acids in the stabilization of the R’‐N_3_‐PR_3_ (**I**) species.[^24^, ^25^]

**Scheme 2:**
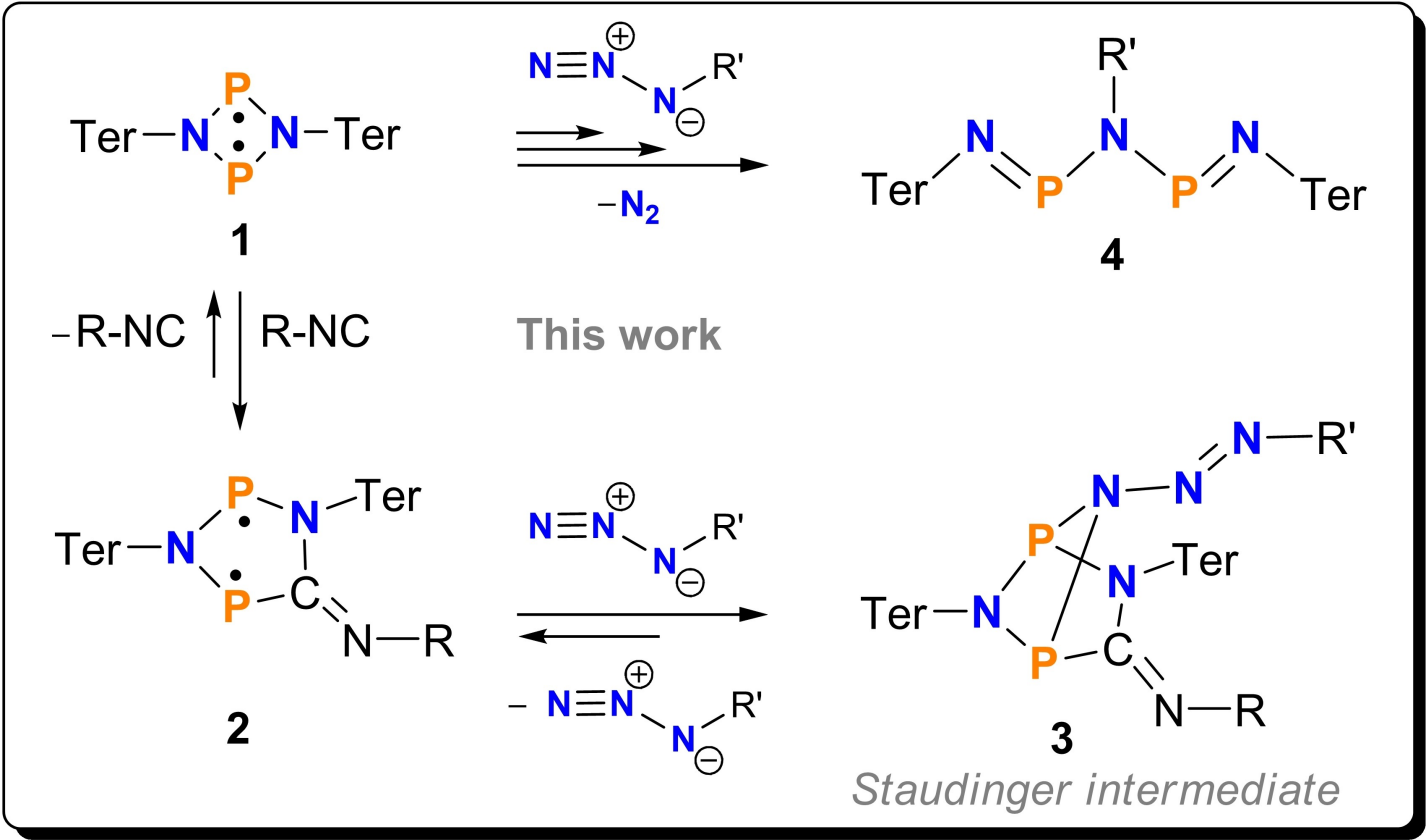
Reaction of biradicaloids **1** and **2** with a covalent azide (R always Mtp=2,6‐dimethyl‐4‐*tert*‐butyl‐phenyl; Ter=2,6‐dimesityl‐phenyl).

Recently, we studied the reaction of cyclic phosphorus‐centered biradicaloid **1** with covalent azides which displayed reaction channels with multiple reaction steps to form Staudinger‐type products **4** (Scheme 2).^[26]^ Cyclic phosphorus‐centered biradicaloids of type **1** and **2**, which were used throughout this work, have been intensely investigated,[^27^, ^28^, ^29^, ^30^, ^31^, ^32^, ^33^] especially for their use in small molecule activation or as molecular photoswitches.[^34^, ^35^, ^36^, ^37^, ^38^, ^39^]

In contrast to the classical Staudinger reaction, which involves one phosphorus(III) center, biradicaloids of type **1** and **2** feature two phosphorus atoms, each with radical character. Therefore, no oxidation to a P^(V)^ species takes place upon reaction with azides, but the formation of a triaza‐diphospha‐pentadiene (**4**) with two P^(III)^ atoms is observed. Since both P atoms in the biradicaloid can be described with the formal oxidation state +II, each P atom is oxidized by the loss of an electron. This also means a total loss of two electrons in agreement with the oxidation of P^(III)^ to P^(V)^ in the common Staudinger reaction (Scheme 1) when only one P^(III)^ atom is involved. So far, along the biradicaloid/azide reaction pathway it was impossible to isolate any of the Staudinger‐type intermediates of type **I** known for the classical Staudinger reaction.^[26]^ Therefore, we changed to a five‐membered biradicaloid (**2**) to reduce ring tension and steric hindrance, which in turn should lead to a stabilization of a Staudinger‐type intermediate product (**3**), now with two phosphorus centers, as depicted in Scheme 2. Here we report the successful isolation of a transient Staudinger‐type intermediate that is stabilized by a diphosphorus‐centered biradicaloid at low temperatures.

## Results and Discussion

### Synthesis

**Scheme 3:**
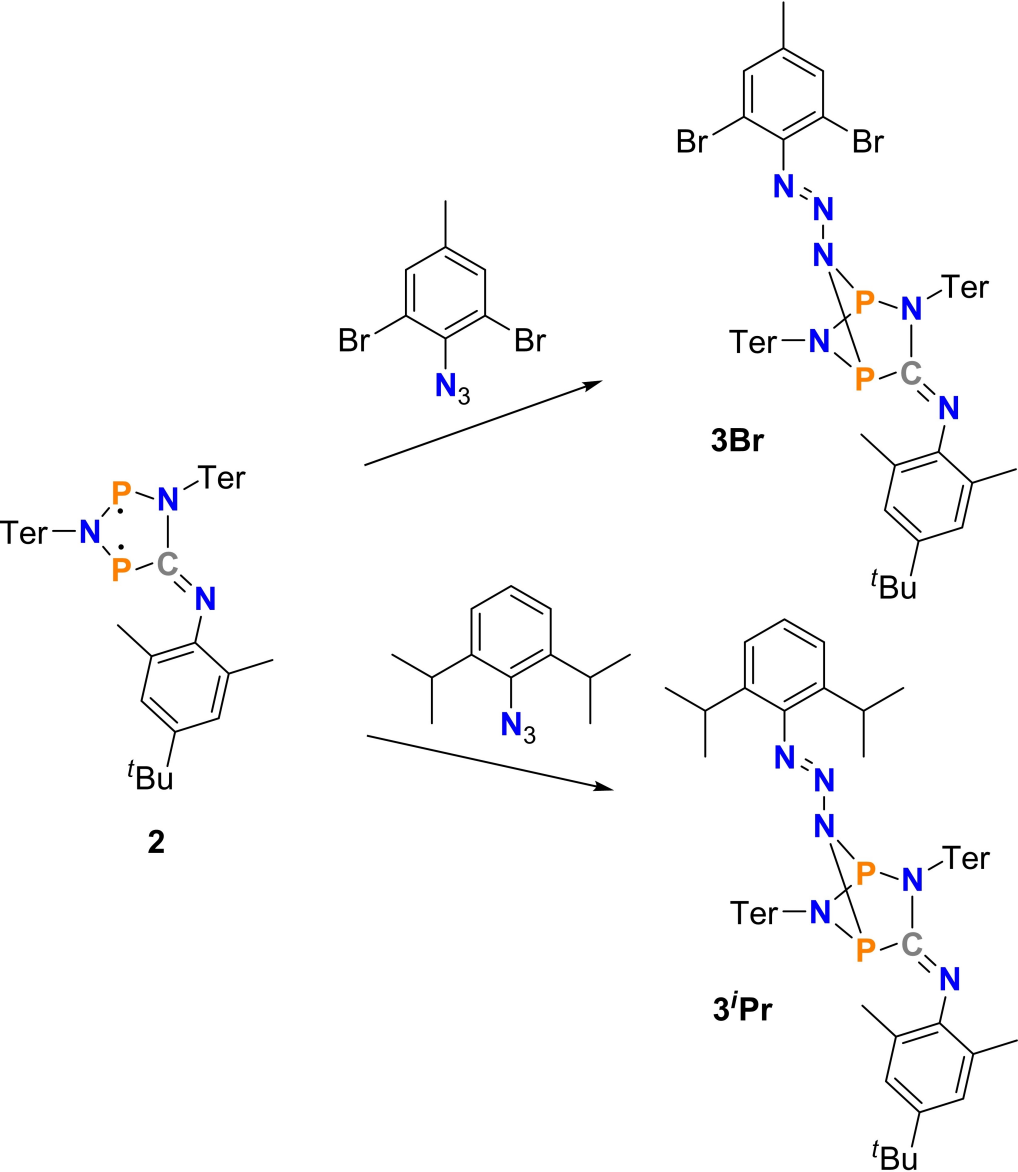
Synthesis of Staudinger‐type adducts **3R′**.

The synthesis of the five‐membered heterocyclic P‐centered biradicaloid **2** succeeds in very good yields (82 %) when biradicaloid **1**, dissolved in benzene, is mixed with an equivalent of 2,6‐dimethyl‐4‐*tert‐*butyl‐phenylisonitrile (Mtp‐NC, Mtp=2,6‐dimethyl‐4‐*tert*‐butyl‐phenyl, Scheme 2).^[40]^ This results in an immediate color change from orange to deep blue, the intrinsic color of biradical **2**. With the easily isolated, blue biradicaloid **2** in hand, we began our investigations into the reaction with covalent organic azides. For this purpose, biradicaloid **2** was reacted with two different organic azides in benzene (Scheme 3). A solution of 2,6‐dibromo‐4‐methylphenyl azide, Dbmp‐N_3_, in benzene was added to a solution of **2** in benzene, and the blue color changed to deep green indicating the formation of a new azide adduct species (**3Br**). The same color change was observed when **2** was reacted with 2,6‐diisopropylphenyl azide. The use of benzene as solvent for the reaction instead of toluene has the advantage that the removal of the solvent can be carried out relatively quickly without extensive heating of the solution. This is essential to keep the addition products **3R′** intact (see below). After removal of the solvent and recrystallization from *n*‐hexane, colorless crystals could be isolated in both cases, which were identified as Staudinger‐type intermediates **3R′** (yields: **3Br** 53 %, **3** ^***i***^ **Pr** 40 %). Single‐crystal X‐ray studies of both species unequivocally revealed the formation of a Staudinger‐type adduct, in which the γ nitrogen atom of the azide is now bound to both biradical phosphorus atoms, in contrast to the classical Staudinger intermediate, in which only one atom is involved in the adduct formation (cf. species **I** in Scheme 1 vs. **3** in Scheme 2).

### Structure

**Figure 1:**
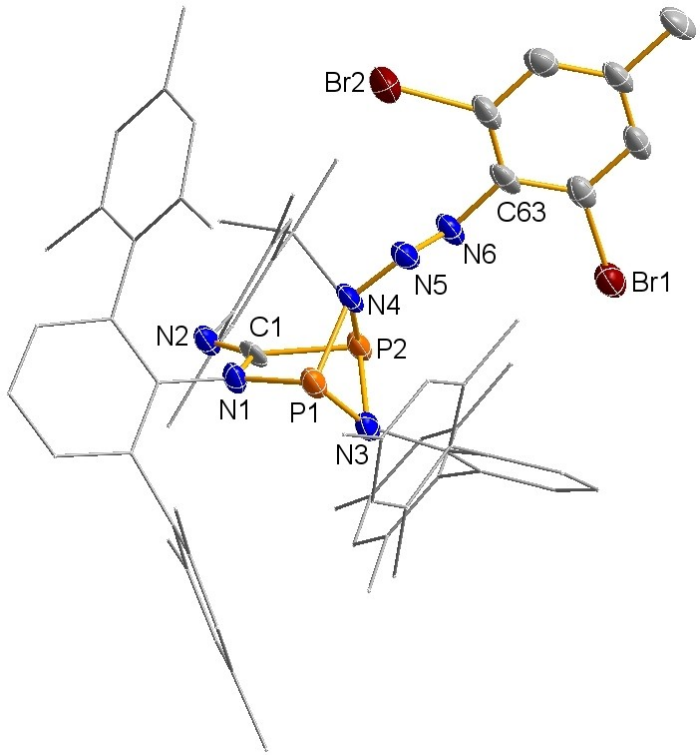
Molecular structure (ORTEP) of **3Br** in the single crystal (*T*=123 K, ellipsoids set at 50 % probability). Hydrogen atoms are omitted for clarity. Ter and Mtp substituents shown as wireframe. Selected bond lengths [Å] and angles [°]: N2−C1 1.252(6), N1−C1 1.417(6), P1−N1 1.716(4), P1−N3 1.775(5), P1−N4 1.804(4), P2−N4 1.796(5), P2−N3 1.778(4), N4−N5 1.346(5), N5−N6 1.275(5), P1⋅⋅⋅P2 2.561(2), N1−P1−N4 90.0(2), N1−P1−N3 92.5(2), N1−P1−N3 92.5(2), P2−N4−P1 90.7(2), N6−N5−N4−112.5(4), N5−N6−C63 113.1(4), P1−N1−C1−P2 4.2(4), P1−N4−P2−N3 −26.5(2), C1−N1−P1−N4 38.9(4), N4−N5−N6−C63 174.7(4).

As only the SCXRD data set for **3Br** was usable, the structural data is only discussed for this species in the following. However, the other data set for **3** ^***i***^ **Pr** ‐although very poor‐ was sufficient for structural proof (Figure S2). The isolation of single crystals of low quality can be ascribed to the fact that the crystals must be formed as quickly as possible, ideally within the first 1–2 h after starting the reaction. As the addition products **3R′** are unstable in solution with respect to the loss of molecular nitrogen at ambient temperatures, prolonged reaction times lead to decomposition and formation of species **4R′** (see below). Colorless crystals of **3Br** crystallized in the triclinic space group *P* $\bar{1}\;$ with two molecules per unit cell (Figure 1). There are no significant intermolecular interactions, however, small intramolecular π‐C_arene_ bromine van der Waals interactions (*d*(Br⋅⋅⋅C_arene_)=3.5 ‐ 4.0 Å; cf. Σ*r*_vdW_(C−Br)=3.63 Å) are present.^[41]^ Probably the most prominent structural motif is the angled five‐membered ring, whereby the P1−N1−C1−P2 fragment remains almost planar (∢(P1−N1−C1−P2)=4.2(4)°). The planarity of the five‐membered heterocycle in **2** is destroyed by the formation of two new P−N_azide_ bonds (1.796(5) and 1.804(4) Å, cf. *d*(P1−N1)=1.716(4) Å), although the P−N_azide_ bond lengths are relatively long and already indicate weak P−N_azide_ bonds. The addition of the azide thus leads to the formation of a [2.1.1] bicyclic compound. The *trans*‐bent azide unit (∢(N4−N5−N6−C63)=174.7°) has a relatively small N−N−N angle with 112.5(4)°, which is usually between 160–180° in organic azides,[^42^, ^43^, ^44^, ^45^, ^46^] and two distinctly different N−N bonds. While the N4−N5 distance is clearly increasedwith 1.346(5) Å upon adduct formation, the N5−N6 distance with 1.275(5) Å is in the range of a N=N double bond (Σ*r*_cov_(N=N)=1.20 Å, Σ*r*_cov_(N−N)=1.42 Å),^[47]^ so that the P_2_N_3_R fragment can also be formally referred to as an amino‐diazene (R_2_N−N=N−R’, R=biradicaloid fragment, R’=organic substituent, Scheme 3).

### Decomposition

Crystals of both azide adducts (**3Br** and **3** ^***i***^ **Pr**) are thermally stable up to just over 120 °C. At this temperature, decomposition takes place with the release of N_2_. In solution, both adducts are not stable with respect to N_2_ release. This prompted us to investigate the decomposition pathway in solution in more detail. Experimentally, we knew that due to the instability of **3** in solution, the yield of isolated **3** is best when the reaction is monitored by ^31^P NMR spectroscopy and rapid recrystallization is performed at the maximum concentration of **3**.

**Figure 2:**
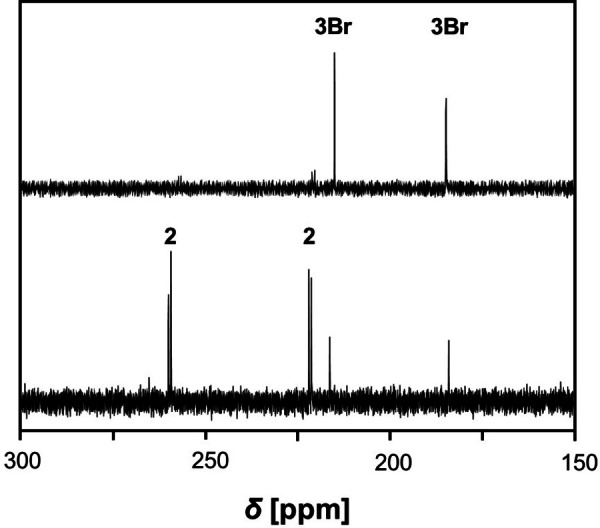
^31^P{^1^H} NMR spectra of freshly dissolved **3Br** at room temperature (bottom) and at −40 °C (top) in THF‐*d*_8_.

**Figure 3:**
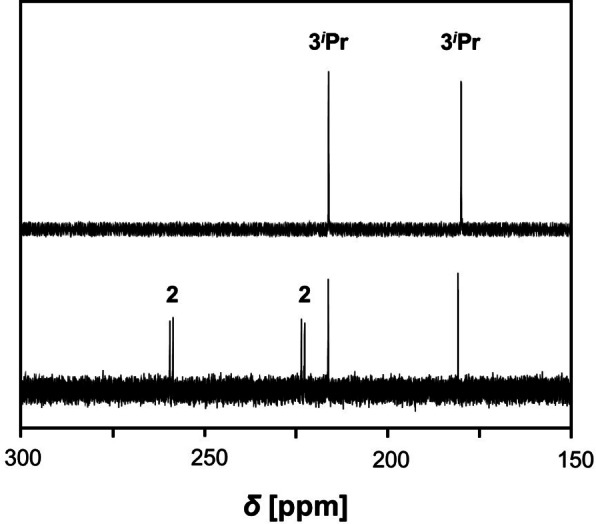
^31^P{^1^H} NMR spectra of freshly dissolved **3** ^***i***^ **Pr** at room temperature (bottom) and at −40 °C (top) in THF‐*d*_8_.

**Figure 4:**
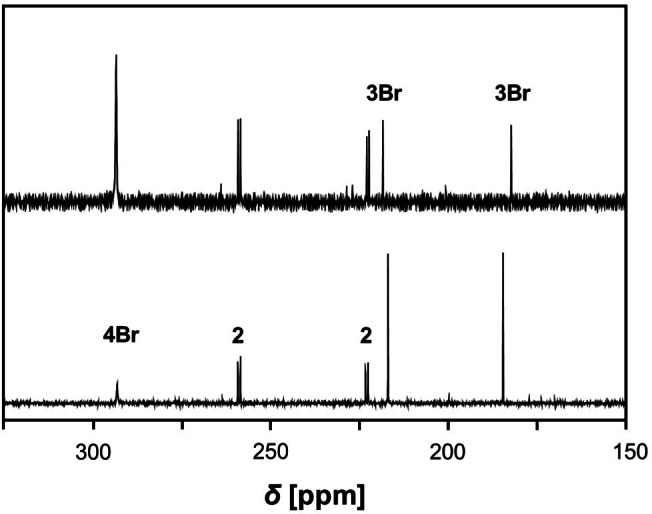
^31^P{^1^H} NMR spectra of the reaction mixture of **2** with Dbmp‐N_3_ after 2 h (bottom) and after 4 days (top) at room temperature.

When colorless pure crystals of **3** were dissolved in THF at temperatures below −40 °C, only the signals of **3** (**3Br**: *δ*=184.5 and 214.8, **3** ^***i***^ **Pr**: 179.9 and 216.1 ppm; Figure 2 and Figure 3) were detected in the ^31^P NMR spectrum. As time passed or the temperature raised slowly to 20 °C, the colorless solution slowly turned deep blue and the reaction back to the free azide and biradicaloid **2** (*δ*=222.7 and 258.7 ppm, Figure S8) was observed (Scheme 2). The concentration of **2** then slowly decreased over time, while a new signal was observed at 293.8 ppm (decomposition of **3Br**, Figure 4) or 297.4 ppm (decomposition of **3** ^***i***^ **Pr**), respectively, which we were able to clearly assign to the Staudinger‐type products **4Br** and **4** ^***i***^ **Pr** (cf. 296.0 ppm for R’=mesityl; Scheme 2).^[26]^ These signals (together with other analytical data after isolation, see ESI) showed us that triaza‐diphospha‐pentadienes, Ter−N=P−N(R’)−P=N−Ter (**4**, Scheme 2), had formed, which we also recently observed in the reaction of the four‐membered biradicaloid **1** with covalent azides.^[26]^ This in turn means that our five‐membered biradical is also always in equilibrium with biradicaloid **1** and free CN−R, although we did not observe free **1**, as it always continues to react immediately with the free azide in a Staudinger‐type reaction with spontaneous N_2_ release (Scheme 2). The test reactions of biradicaloid **1** with both azides, R’‐N_3_, led to a spontaneous vigorous reaction, in which N_2_ release and the formation of triaza‐diphospha‐pentadienes **4R′** was observed (see ESI sections 4.3 and 4.4). The first triaza‐diphospha‐pentadiene was described by Niecke et al. which was obtained in the reaction of chloro(aryl‐imino)phosphanes with an 1,3‐diaza‐2‐phosphaallylic species.[^48^, ^49^]

### Computations

Since, we have recently studied the reaction of biradicaloid **1** with organic azides in detail both theoretically and experimentally, we would like to refer to the literature at this point.^[26]^ Using quantum chemical calculations at the PBE−D3/def2‐TZVP (for geometry optimization)[^50^, ^51^, ^52^, ^53^, ^54^, ^55^, ^56^] and single‐point DLPNO‐CCSD(T) (for accurate energies) level of theory,[^57^, ^58^, ^59^, ^60^] we therefore only focused on the kinetics and thermodynamics of the azide addition reaction resulting in the formation of **3**.

**Figure 5:**
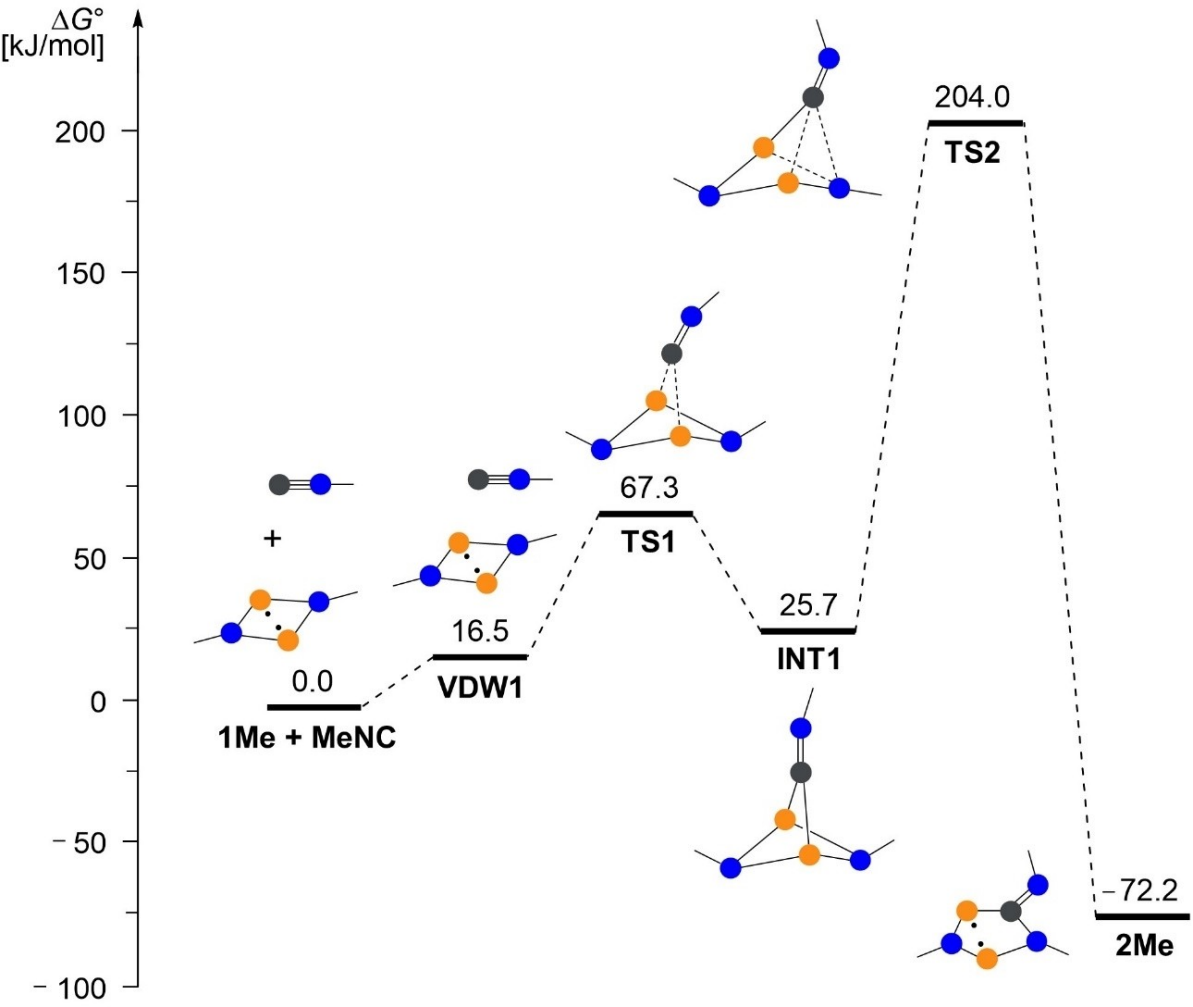
Computed reaction path (A) for the formation of **2Me** at the DLPNO‐CCSD(T)/def2‐TZVP//PBE‐D3/def2‐TZVP level of theory (*c*°=1 mol/L).

**Figure 6:**
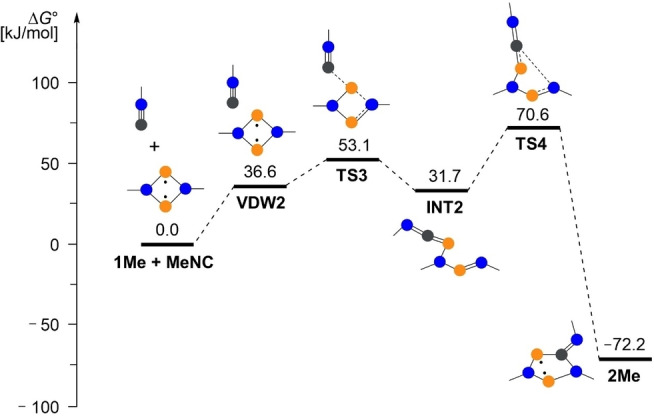
Computed reaction path (B) for the formation of **2Me** at the DLPNO‐CCSD(T)/def2‐TZVP//PBE‐D3/def2‐TZVP level of theory (*c*°=1 mol/L).

First, we looked at the formation of the five‐membered ring starting from **1**. The reaction mechanism of the isonitrile insertion reaction was initially studied using a simple model system in which all bulky substituents (Ter and Mtp) were replaced by methyl groups. Two different reaction pathways were found: (i) The addition of the isonitrile group via the C atom to both P atoms, followed by a rearrangement reaction with insertion of the CN group into a P−N bond (path A, Figure 5). However, the activation energies here are very high at 204.0 kJ/mol (TS2) for the second step. Therefore, it seems more likely that the second reaction pathway is used as depicted in Figure 6 (path B), which leads from a van der Waals adduct of the isonitrile on the biradicaloid via an open chain intermediate to the five‐membered heterocyclic product **2Me**. Here, the highest activation barrier is only 70.6 kJ/mol (TS4). For both reaction channels, the overall process is exergonic with −72.2 kJ/mol.

**Figure 7:**
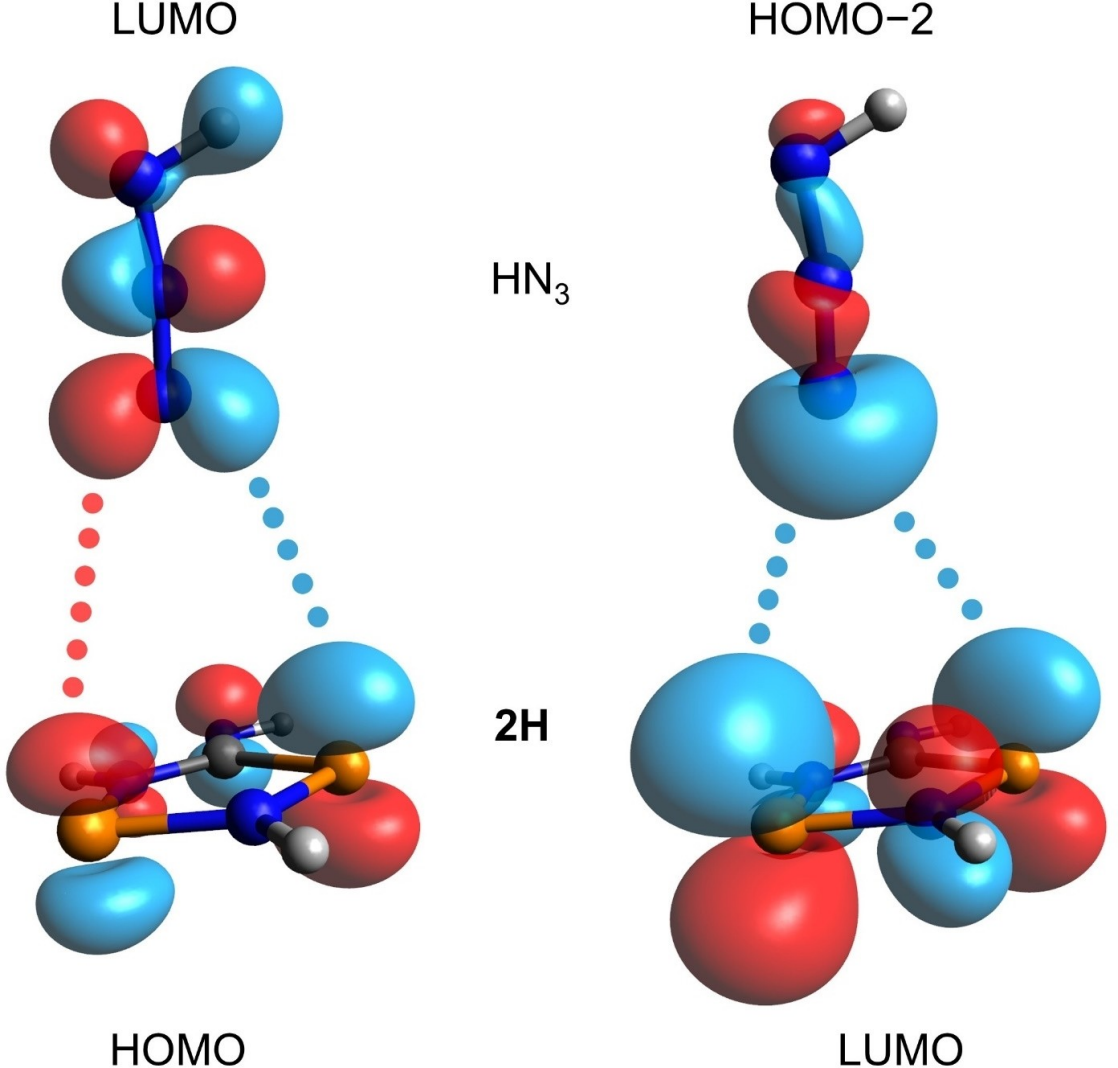
Schematic representation of the interaction of the frontier MOs of the model system **2H** (*C*_s_ symmetry) and HN_3_ (*C*_s_ symmetry).

**Figure 8:**
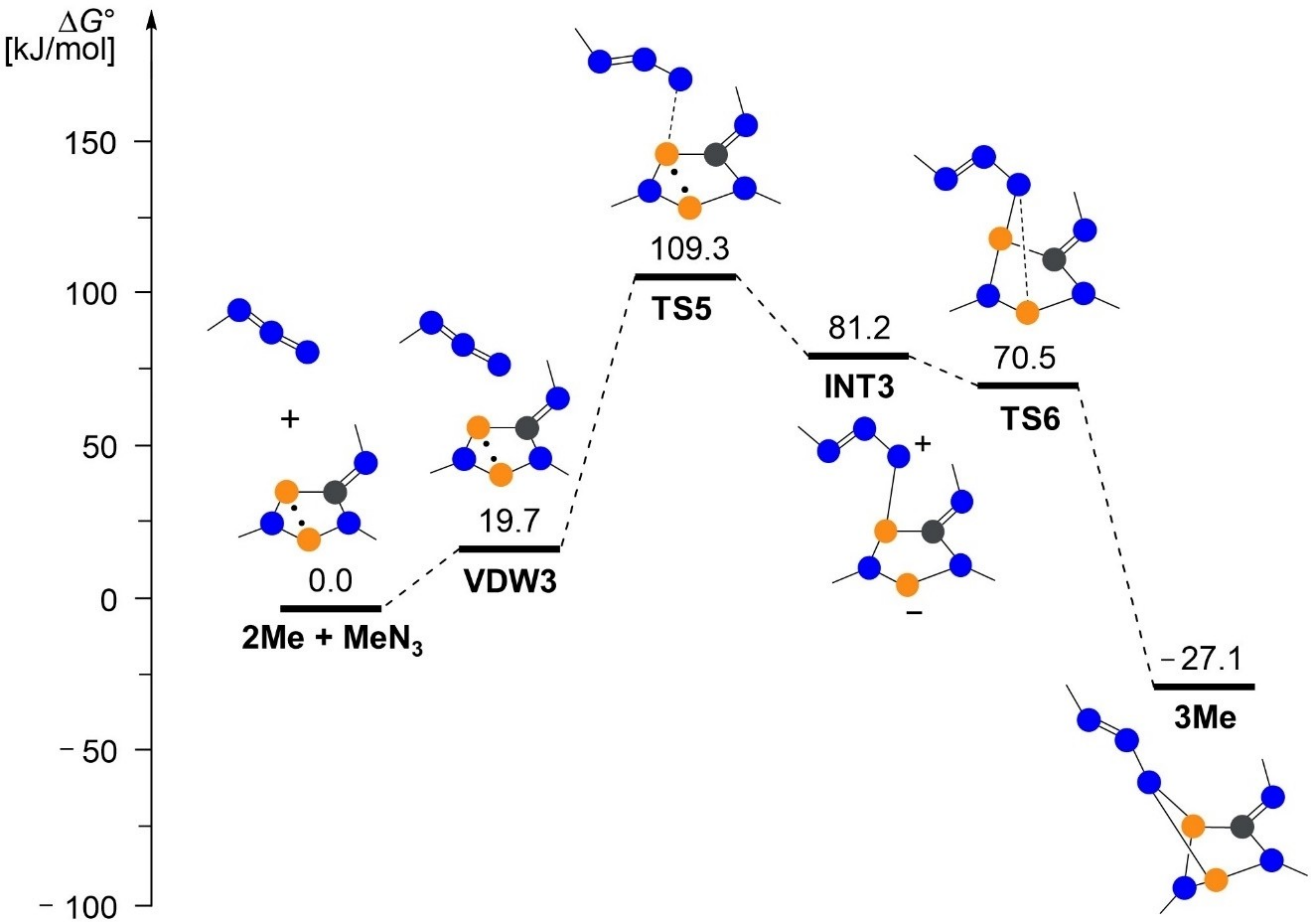
Computed reaction path for the reaction of MeN_3_ with **2Me** at the DLPNO‐CCSD(T)/def2‐TZVP//PBE‐D3/def2‐TZVP level of theory (*c*°=1 mol/L).

In a second series of calculations, we looked at the addition reaction of the covalent azide to biradicaloid **2**. The symmetry of the frontier orbitals (Figure 7 for the model system **2H** / HN_3_) indicated a concerted mechanism for the addition of HN_3_ to **2H**. The search for a transition state (TS), however, proved to be rather difficult. For the model system **2Me** / MeN_3_, we could only find a two‐step mechanism with relatively small activation barriers of 109.3 and 70.5 kJ/mol, respectively (Figure 8). At this theoretical level (DLPNO‐CCSD(T)/def2‐TZVP//PBE−D3/def2‐TZVP), the overall process is slightly exergonic with −27.1 kJ/mol.

**Figure 9:**
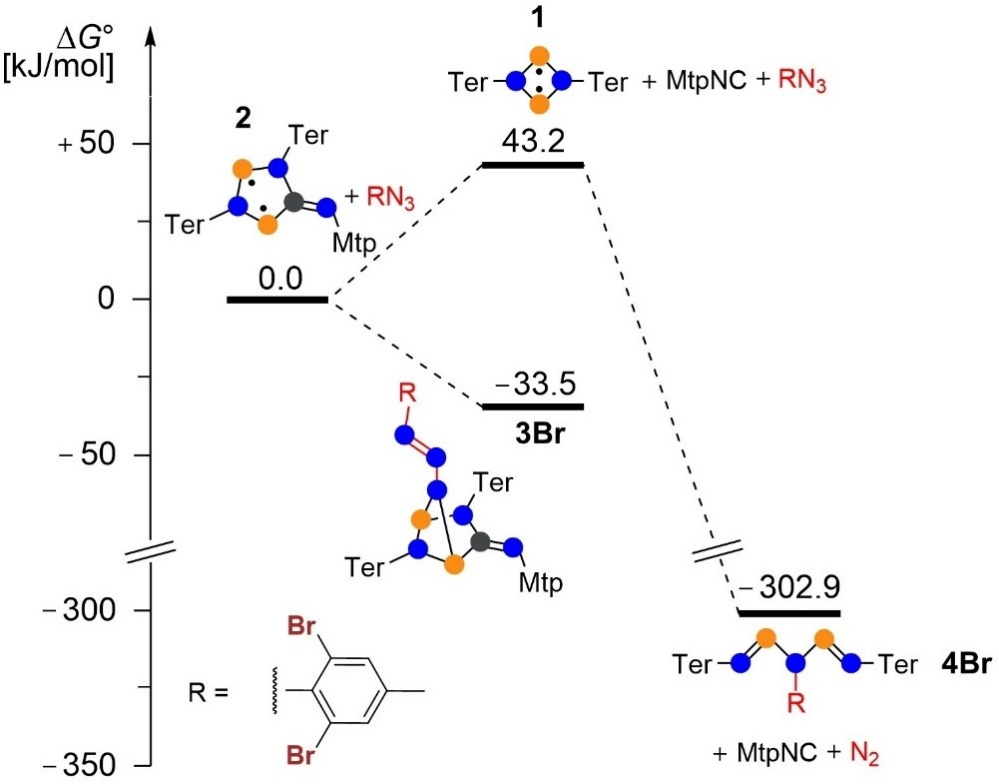
Gibbs free energies of the reaction of Dbmp‐N_3_ with **2** at the DLPNO‐CCSD(T)/def2‐TZVP//PBE‐D3/def2‐TZVP level of theory (c°=1 mol/L).

In order to obtain more accurate and meaningful thermodynamic data, we performed single point calculations for the experimentally observed system **3Br** at the DLPNO‐CCSD(T)/def2‐TZVP//PBE‐D3/def2‐TZVP level of theory (Figure 9). Unfortunately, it was not possible to scan the entire potential energy surface at this level for the real system, as it is significantly flatter and, above all, not feasible with so many atoms. However, in comparison with the model system, it is noticeable that (i) the bulky substituents destabilize the five‐membered biradicaloid compared to the reverse reaction (**1** + CN‐Mtp), probably due to the larger Pauli repulsion (cf. **3Br**: −43.2 vs. **3Me**:−72.2 kJ/mol) and (ii) the azide adduct **3Br** is thermodynamically slightly more stabilized compared to the starting compounds with −33.5 kJ/mol. In agreement with the experimental observations, the Staudinger‐type products **4Br** + Mtp‐NC + N_2_ are the thermodynamic sink with −302.9 kJ/mol compared to −33.5 kJ/mol for the azide adduct **3Br**.

The NBO analysis of compound **3Br** finds the Lewis representation shown in Scheme 3 as one of the energetically preferred formula, i. e. trivalent P atoms and a formal amino function of the terminal N atom of the previous azide group. The P−N bonds are all strongly polarized, and the charge transfer from the biradicaloid to the azide is 0.9 electrons, i. e. the biradicaloid moiety is almost monocationic in the adduct **3Br**.

## Conclusions

In summary, we have succeeded for the first time in binding an intact organic azide to a P‐centered biradicaloid. The addition products can be considered as Staudinger‐type intermediates involving two P atoms. Both P atoms are oxidized from the formal oxidation state +II to +III when the azide releases molecular nitrogen. This represents a novel reaction behavior, in contrast to the classical Staudinger intermediates, which consist of only one P^(III)^ atom bound to the azide (see Schemes 1 and 2) that is oxidized to P(V) when N_2_ is eliminated. However, both in the formation of our Staudinger intermediates and in the classical Staudinger intermediates, the azide fragment remains intact, and a 2‐electron‐oxidation takes place to form the Staudinger or Staudinger‐type products, respectively. Experiments show that when the azide is added to biradicaloid **2**, azide adduct **3** is formed, which is, however, in equilibrium with **2** and the azide. The biradicaloid **2** in turn is in equilibrium with the four‐membered biradicaloid **1** and isonitrile CN−R. It is known that the biradicaloid **1** reacts very rapidly with the azide at ambient temperatures and forms a triaza‐diphospha‐pentadiene **4** in a strongly exergonic reaction with elimination of molecular nitrogen. Therefore, this reaction dominates at ambient temperaturesand azide adduct **3** must be isolated quickly as it is only reasonably stable in terms of N_2_ release in the solid state at low temperatures.

## Supporting Information

The authors have cited additional references within the Supporting Information[^61^, ^62^, ^63^, ^64^, ^65^, ^66^, ^67^, ^68^, ^69^, ^70^, ^71^, ^72^, ^73^, ^74^, ^75^, ^76^, ^77^, ^78^, ^79^, ^80^, ^81^, ^82^, ^83^, ^84^, ^85^, ^86^, ^87^, ^88^, ^89^, ^90^, ^91^] alongside deposition numbers for supplementary crystallographic data.^[92]^

## Conflict of Interests

The authors declare no conflict of interest.

1

## Supporting information

As a service to our authors and readers, this journal provides supporting information supplied by the authors. Such materials are peer reviewed and may be re‐organized for online delivery, but are not copy‐edited or typeset. Technical support issues arising from supporting information (other than missing files) should be addressed to the authors.

Supporting Information

## Data Availability

The data that support the findings of this study are available in the supplementary material of this article.

## References

[1] H. Staudinger , J. Meyer , Helv. Chim. Acta 1919, 2, 635–646.

[2] H. Staudinger , E. Hauser , Helv. Chim. Acta 1921, 4, 861–886.

[3] J. E. Leffler , R. D. Temple , J. Am. Chem. Soc. 1967, 89, 5235–5246.

[4] Y. G. Gololobov , I. N. Zhmurova , L. F. Kasukhin , Tetrahedron 1981, 37, 437–472.

[5] Y. G. Gololobov , L. F. Kasukhin , Tetrahedron 1992, 48, 1353–1406.

[6] M. Desamparados Velasco , P. Molina , P. M. Fresneda , M. A. Sanz , Tetrahedron 2000, 56, 4079–4084.

[7] R. Mülhaupt , Angew. Chem. Int. Ed. 2004, 116, 1072–1080.

[8] S. Bräse , C. Gil , K. Knepper , V. Zimmermann , Angew. Chem. Int. Ed. 2005, 44, 5188–5240. 10.1002/anie.200400657 16100733

[9] S. S. van Berkel , M. B. van Eldijk , J. C. M. van Hest , Angew. Chem. Int. Ed. 2011, 50, 8806–8827. 10.1002/anie.201008102 21887733

[10] M. W. P. Bebbington , D. Bourissou , Coord. Chem. Rev. 2009, 253, 1248–1261.

[11] F. L. Lin , H. M. Hoyt , H. Van Halbeek , R. G. Bergman , C. R. Bertozzi , J. Am. Chem. Soc. 2005, 127, 2686–2695. 15725026 10.1021/ja044461m

[12] P. M. Fresneda , M. Castañeda , M. A. Sanz , P. Molina , Tetrahedron Lett. 2004, 45, 1655–1657.

[13] R. D. Kennedy , Chem. Commun. 2010, 46, 4782. 10.1039/c0cc00426j 20490418

[14] A. V. Alexandrova , T. Mašek , S. M. Polyakova , I. Císařová , J. Saame , I. Leito , I. M. Lyapkalo , Eur. J. Org. Chem. 2013,, 1811–1823.

[15] J. F. Kögel , N. C. Abacılar , F. Weber , B. Oelkers , K. Harms , B. Kovačević , J. Sundermeyer , Chem. Eur. J. 2014, 20, 5994–6009. 24687589 10.1002/chem.201304498

[16] D. H. A. Boom , A. R. Jupp , M. Nieger , A. W. Ehlers , J. C. Slootweg , Chem. Eur. J. 2019, 25, 13299–13308. 31497899 10.1002/chem.201902710 PMC6851766

[17] C. M. Mömming , G. Kehr , B. Wibbeling , R. Fröhlich , G. Erker , Dalton Trans. 2010, 39, 7556. 20617234 10.1039/c0dt00015a

[18] A. Stute , L. Heletta , R. Fröhlich , C. G. Daniliuc , G. Kehr , G. Erker , Chem. Commun. 2012, 48, 11739. 10.1039/c2cc36782c 23111350

[19] X. Xu , G. Kehr , C. G. Daniliuc , G. Erker , J. Am. Chem. Soc. 2013, 135, 6465–6476. 23528161 10.1021/ja3110076

[20] J. Schneider , K. M. Krebs , S. Freitag , K. Eichele , H. Schubert , L. Wesemann , Chem. Eur. J. 2016, 22, 9812–9826. 27273819 10.1002/chem.201601224

[21] L. Keweloh , H. Klöcker , E. Würthwein , W. Uhl , Angew. Chem. Int. Ed. 2016, 55, 3212–3215. 10.1002/anie.201511048 26836748

[22] L. Elmer , G. Kehr , C. G. Daniliuc , M. Siedow , H. Eckert , M. Tesch , A. Studer , K. Williams , T. H. Warren , G. Erker , Chem. Eur. J. 2017, 23, 6056–6068. 27925311 10.1002/chem.201603954

[23] J. Backs , M. Lange , J. Possart , A. Wollschläger , C. Mück-Lichtenfeld , W. Uhl , Angew. Chem. Int. Ed. 2017, 56, 3094–3097. 10.1002/anie.201612485 28156031

[24] T. K. K. Dickie , P. G. Hayes , Organometallics 2022, 41, 278–283.

[25] T. K. K. Dickie , C. S. MacNeil , P. G. Hayes , Dalton Trans. 2020, 49, 578–582. 31808763 10.1039/c9dt04517a

[26] A. Schulz , A. Hinz , A. Rölke , A. Villinger , R. Wustrack , Z. Anorg. Allg. Chem. 2021, 647, 245–257.

[27] T. Beweries , R. Kuzora , U. Rosenthal , A. Schulz , A. Villinger , Angew. Chem. Int. Ed. 2011, 50, 8974–8978. 10.1002/anie.201103742 21858902

[28] A. Hinz , R. Kuzora , U. Rosenthal , A. Schulz , A. Villinger , Chem. Eur. J. 2014, 20, 14659–14673. 25266101 10.1002/chem.201403964

[29] A. Hinz , A. Schulz , A. Villinger , Chem. Eur. J. 2014, 20, 3913–3916. 24615784 10.1002/chem.201400143

[30] A. Hinz , A. Schulz , A. Villinger , Angew. Chem. Int. Ed. 2015, 54, 2776–2779. 10.1002/anie.201410276 25604347

[31] A. Hinz , A. Schulz , A. Villinger , J. Am. Chem. Soc. 2015, 137, 9953–9962. 26204242 10.1021/jacs.5b05596

[32] A. Schulz , Dalton Trans. 2018, 47, 12827–12837. 30183025 10.1039/c8dt03038c

[33] J. Bresien , L. Eickhoff , A. Schulz , E. Zander , in Comprehensive Inorganic Chemistry III (Eds.: J. Reedijk , K. R. Poeppelmeier ), Elsevier, 2021, pp. 1–68.

[34] J. Bresien , T. Kröger-Badge , S. Lochbrunner , D. Michalik , H. Müller , A. Schulz , E. Zander , Chem. Sci. 2019, 10, 3486–3493. 30996939 10.1039/c8sc04893b PMC6430090

[35] J. Rosenboom , A. Villinger , A. Schulz , J. Bresien , Dalton Trans. 2022, 51, 13479–13487. 35997123 10.1039/d2dt02229j

[36] J. Bresien , D. Michalik , A. Schulz , A. Villinger , E. Zander , Angew. Chem. Int. Ed. 2021, 60, 1507–1512. 10.1002/anie.202011886 PMC7839750 33038288

[37] E. Zander , J. Bresien , V. V. Zhivonitko , J. Fessler , A. Villinger , D. Michalik , A. Schulz , J. Am. Chem. Soc. 2023, 145, 14484–14497. 37315222 10.1021/jacs.3c03928 PMC10368346

[38] A. Hinz , J. Bresien , F. Breher , A. Schulz , Chem. Rev. 2023, 123, 10468–10526. 37556842 10.1021/acs.chemrev.3c00255

[39] M. Abe , Chem. Rev. 2013, 113, 7011–7088. 23883325 10.1021/cr400056a

[40] Y. Pilopp , J. Bresien , D. T. Gschwind , A. Villinger , D. Michalik , A. Schulz , Chem. Eur. J. 2023, 29, e202300764. 36947665 10.1002/chem.202300764

[41] S. Alvarez , Dalton Trans. 2013, 42, 8617–8636. 23632803 10.1039/c3dt50599e

[42] I. C. Tornieporth-Oetting , T. M. Klapötke , Angew. Chem. Int. Ed. Engl. 1995, 34, 511–520.

[43] Z. Dori , R. F. Ziolo , Chem. Rev. 1973, 73, 247–254.

[44] E. F. V. Scriven , K. Turnbull , Chem. Rev. 1988, 88, 297–368.

[45] P. Portius , M. Davis , Coord. Chem. Rev. 2013, 257, 1011–1025.

[46] L. Zhu , R. Kinjo , Chem. Soc. Rev. 2023, 52, 5563–5606. 37519098 10.1039/d3cs00290j

[47] P. Pyykkö , M. Atsumi , Chem. Eur. J. 2009, 15, 12770–12779. 19856342 10.1002/chem.200901472

[48] E. Niecke , R. Detsch , M. Nieger , Chem. Ber. 1990, 123, 797–799.

[49] R. Detsch , E. Niecke , M. Nieger , F. Reichert , Chem. Ber. 1992, 125, 321–330.

[50] J. P. Perdew , K. Burke , M. Ernzerhof , Phys. Rev. Lett. 1996, 77, 3865–3868. 10062328 10.1103/PhysRevLett.77.3865

[51] J. P. Perdew , K. Burke , M. Ernzerhof , Phys. Rev. Lett. 1997, 78, 1396–1396. 10.1103/PhysRevLett.77.3865 10062328

[52] C. Adamo , V. Barone , J. Chem. Phys. 1999, 110, 6158–6170.

[53] S. Grimme , J. Antony , S. Ehrlich , H. Krieg , J. Chem. Phys. 2010, 132, 154104. 20423165 10.1063/1.3382344

[54] S. Grimme , S. Ehrlich , L. Goerigk , J. Comput. Chem. 2011, 32, 1456–1465. 21370243 10.1002/jcc.21759

[55] F. Weigend , R. Ahlrichs , Phys. Chem. Chem. Phys. 2005, 7, 3297. 16240044 10.1039/b508541a

[56] F. Weigend , Phys. Chem. Chem. Phys. 2006, 8, 1057–1065. 16633586 10.1039/b515623h

[57] C. Riplinger , F. Neese , J. Chem. Phys. 2013, 138, 034106. 23343267 10.1063/1.4773581

[58] D. G. Liakos , Y. Guo , F. Neese , J. Phys. Chem. A 2020, 124, 90–100. 31841627 10.1021/acs.jpca.9b05734

[59] D. G. Liakos , M. Sparta , M. K. Kesharwani , J. M. L. Martin , F. Neese , J. Chem. Theory Comput. 2015, 11, 1525–1539. 26889511 10.1021/ct501129s

[60] C. Riplinger , P. Pinski , U. Becker , E. F. Valeev , F. Neese , J. Chem. Phys. 2016, 144, 024109. 26772556 10.1063/1.4939030

[61] H. Braunschweig , F. Hupp , I. Krummenacher , L. Mailänder , F. Rauch , Chem. Eur. J. 2015, 21, 17844–17849. 26482113 10.1002/chem.201503048

[62] A. A. Ageshina , G. A. Chesnokov , M. A. Topchiy , I. V. Alabugin , M. S. Nechaev , A. F. Asachenko , Org. Biomol. Chem. 2019, 17, 4523–4534. 30994147 10.1039/c9ob00615j

[63] K. Tanaka, A. R. Pradipta (RIKEN), *EP4233916 A1*, **2023**.

[64] B. L. Small , R. Rios , E. R. Fernandez , D. L. Gerlach , J. A. Halfen , M. J. Carney , Organometallics 2010, 29, 6723–6731.

[65] S. Wu , J. Huang , S. Gazzarrini , S. He , L. Chen , J. Li , L. Xing , C. Li , L. Chen , C. G. Neochoritis , G. P. Liao , H. Zhou , A. Dömling , A. Moroni , W. Wang , ChemMedChem 2015, 10, 1837–1845. 26506405 10.1002/cmdc.201500318

[66] F. Reiß , A. Schulz , A. Villinger , N. Weding , Dalton Trans. 2010, 39, 9962. 20842305 10.1039/c0dt00700e

[67] G. M. Sheldrick , Acta Crystallogr. Sect. A Found. Adv. 2015, 71, 3–8. 25537383 10.1107/S2053273314026370 PMC4283466

[68] G. M. Sheldrick , Acta Crystallogr. Sect. C Struct. Chem. 2015, 71, 3–8. 25567568 10.1107/S2053229614024218 PMC4294323

[69] G. M. Sheldrick, *SADABS Version 2*, University of Göttingen, Germany, **2004**.

[70] *Gaussian 09, Revision E.01*, M. J. Frisch, G. W. Trucks, H. B. Schlegel, G. E. Scuseria, M. A. Robb, J. R. Cheeseman, G. Scalmani, V. Barone, B. Mennucci, G. A. Petersson, H. Nakatsuji, M. Caricato, X. Li, H. P. Hratchian, A. F. Izmaylov, J. Bloino, G. Zheng, J. L. Sonnenberg, M. Hada, M. Ehara, K. Toyota, R. Fukuda, J. Hasegawa, M. Ishida, T. Nakajima, Y. Honda, O. Kitao, H. Nakai, T. Vreven, J. A. Montgomery Jr., J. E. Peralta, F. Ogliaro, M. Bearpark, J. J. Heyd, E. Brothers, K. N. Kudin, V. N. Staroverov, T. Keith, R. Kobayashi, J. Normand, K. Raghavachari, A. Rendell, J. C. Burant, S. S. Iyengar, J. Tomasi, M. Cossi, N. Rega, J. M. Millam, M. Klene, J. E. Knox, J. B. Cross, V. Bakken, C. Adamo, J. Jaramillo, R. Gomperts, R. E. Stratmann, O. Yazyev, A. J. Austin, R. Cammi, C. Pomelli, J. W. Ochterski, R. L. Martin, K. Morokuma, V. G. Zakrzewski, G. A. Voth, P. Salvador, J. J. Dannenberg, S. Dapprich, A. D. Daniels, O. Farkas, J. B. Foresman, J. V. Ortiz, J. Cioslowski, D. J. Fox, Gaussian, Inc., Wallingford CT, **2013**.

[71] F. Neese, *The ORCA program system*, Wiley Interdiscip. Rev.: Comput. Mol. Sci. **2012**,* 2*, 1, 73–78.

[72] E. D. Glendening, J. K. Badenhoop, A. E. Reed, J. E. Carpenter, J. A. Bohmann, C. M. Morales, C. R. Landis, F. Weinhold, *NBO 6.0*, Theoretical Chemistry Institute, University of Wisconsin, Madison, **2013**.

[73] F. Weinhold , J. E. Carpenter , in The Structure of Small Molecules and Ions (Eds.: R. Naaman , Z. Vager ), Springer, Boston, MA, 1988, pp. 227–236.

[74] F. Weinhold , C. R. Landis , Valency and Bonding. A Natural Bond Orbital Donor-Acceptor Perspective, Cambridge University Press, 2005.

[75] F. London , J. Phys. Radium 1937, 8, 397–409.

[76] R. McWeeny , Phys. Rev. 1962, 126, 1028–1034.

[77] R. Ditchfield , Mol. Phys. 1974, 27, 789–807.

[78] K. Wolinski , J. F. Hinton , P. Pulay , J. Am. Chem. Soc. 1990, 112, 8251–8260.

[79] J. R. Cheeseman , G. W. Trucks , T. A. Keith , M. J. Frisch , J. Chem. Phys. 1996, 104, 5497–5509.

[80] A. Hellweg , C. Hättig , S. Höfener , W. Klopper , Theor. Chem. Acc. 2007, 117, 587–597.

[81] C. J. Jameson , A. De Dios , A. Keith Jameson , Chem. Phys. Lett. 1990, 167, 575–582.

[82] C. van Wüllen , Phys. Chem. Chem. Phys. 2000, 2, 2137–2144.

[83] W. Deng , J. R. Cheeseman , M. J. Frisch , J. Chem. Theory Comput. 2006, 2, 1028–1037. 26633062 10.1021/ct600110u

[84] C. J. Cramer , Essentials of Computational Chemistry: Theories and Models, John Wiley & Sons, Ltd, Chichester, UK, 2004.

[85] G. Mills , H. Jónsson , G. K. Schenter , Surf. Sci. 1995, 324, 305–337.

[86] H. Jónsson , G. Mills , K. W. Jacobsen , in Classical and Quantum Dynamics in Condensed Phase Simulations (Eds.: B. J. Berne , G. Ciccotti , D. F. Coker ), World Scientific, Singapore, 1998, pp. 385–404.

[87] G. Henkelman , H. Jónsson , J. Chem. Phys. 2000, 113, 9978–9985.

[88] G. Henkelman , B. P. Uberuaga , H. Jónsson , J. Chem. Phys. 2000, 113, 9901–9904.

[89] E. Maras , O. Trushin , A. Stukowski , T. Ala-Nissila , H. Jónsson , Comput. Phys. Commun. 2016, 205, 13–21.

[90] V. Ásgeirsson , B. O. Birgisson , R. Bjornsson , U. Becker , F. Neese , C. Riplinger , H. Jónsson , J. Chem. Theory Comput. 2021, 17, 4929–4945. 34275279 10.1021/acs.jctc.1c00462

[91] J. E. Carpenter , F. Weinhold , J. Mol. Struct. 1988, 169, 41–62.

[92] Deposition number 2371934 (for **3Br**), contains the supplementary crystallographic data for this paper. These data are provided free of charge by the joint Cambridge Crystallographic Data Centre and Fachinformationszentrum Karlsruhe Access Structures service.

