# Supplementary material for: Isolation of a Staudinger‐type Intermediate Utilizing a Five‐Membered Phosphorus‐Centered Biradicaloid
Source: Chemistry. 2024 Dec 13;31(8):e202403893. doi: 10.1002/chem.202403893 (PMC11803359; doi:10.1002/chem.202403893)
Supplement: Supplementary file 1 — Supporting Information [file CHEM-31-e202403893-s001.pdf]

Chemistry–A European Journal

Supporting Information

Isolation of a Staudinger-type Intermediate Utilizing a Five-Membered Phosphorus-Centered Biradicaloid

Y. Pilopp, J. Bresien, K. P. Lüdtke, and A. Schulz*

SUPPORTING INFORMATION

Isolation of a Staudinger-type Intermediate Utilizing a Five-membered Phosphorus-centered Biradicaloid

Y. Pilopp, J. Bresien, K. P. Lüdtke, A. Schulz

This file includes:

1 Experimental 3

2 Structure elucidation 5

3 Syntheses of starting materials 8

4 Syntheses of compounds 12

5 Additional spectroscopic details 30

6 Computational details 37

7 References 65

1 Experimental

General Information. If not stated otherwise, all manipulations were carried out under oxygen- and moisture-free conditions under an inert atmosphere of argon using standard Schlenk or Drybox techniques. All glassware was heated three times *in vacuo* using a heat gun and cooled under argon atmosphere. Solvents were transferred using syringes, which were purged three times with argon prior to use. Solvents and reactants were either obtained from commercial sources or synthesized as detailed in Table S1.

Table S1: Origin and purification of solvents and reactants.

Substance	Origin	Purification
n-hexane	local trade	dried over Na/benzophenone/tetraglyme<br>freshly distilled prior to use
C_6H_6	local trade	dried over Na/benzophenone<br>freshly distilled prior to use
THF-d_8	euriso-top	dried over Na<br>distilled and stored over molecular sieves (4 Å)
C_6D_6	euriso-top	dried over Na<br>freshly distilled prior to use
2,6-dibromo-4-methylphenylazide	synthesized[1,2]	column chromatography and re-crystallization as described in literature[1,2]
2,6-diisopropyl-phenylazide	synthesized[3]	column chromatography as described in literature[3]
MtpNC	synthesized[4–6]	re-crystallized as described in literature[4–6]
[P(μ-NTer)]$_2$	synthesized[7–9]	re-crystallized as described in literature[7–9]

NMR spectra were recorded on a Bruker spectrometer (AVANCE 500) or a Jeol spectrometer (JNM-ECZL 400) and were referenced internally to the deuterated solvent (C_6D_6 δ_{ref} = 128.4 ppm, THF-d_7 $\delta_{ref,1}$ = 25.3 ppm, $\delta_{ref,2}$ = 67.2 ppm), to the protonated species in the deuterated solvent (C_6HD_5 δ_{ref} = 7.16 ppm, THF-d_7 $\delta_{ref,1}$ = 1.73 ppm, $\delta_{ref,2}$ = 3.58 ppm) or externally (^{31}P: 85% H_3PO_4 $\delta_{ref.}$ = 0 ppm). All measurements were carried out at ambient temperature unless denoted otherwise. NMR signals were assigned using experimental data (e.g. chemical shifts, coupling constants, integrals where applicable) in conjunction with computed NMR data (GIAO method, *cf.* section 6.1). The signs of $^{n}J(^{31}P,^{31}P)$ coupling constants were derived from calculated values.

IR spectra of crystalline samples were recorded on a Bruker Alpha II FT-IR spectrometer equipped with an ATR unit at ambient temperature under argon atmosphere. Relative intensities are reported according to the following intervals: very weak (vw, 0–10%), weak (w, 10–30%), medium (m, 30–60%), strong (s, 60–90%), very strong (vs, 90–100%).

Raman spectra of crystalline samples were recorded using a LabRAM HR 800 Horiba Jobin YVON Raman spectrometer equipped with an Olympus BX41 microscope with variable lenses. The samples were excited by a red laser (633 nm, 17 mW, air-cooled HeNe laser). All measurements were carried out at ambient temperature unless stated otherwise.

Elemental analyses were obtained using an Elementar vario Micro cube CHNS analyser.

Melting points (uncorrected) were determined using a Stanford Research Systems EZ Melt at a heating rate of 20 °C/min. Clearing points are reported.

Mass spectra were recorded on a Thermo Electron MAT 95-XP sector field mass spectrometer using crystalline samples.

2 Structure elucidation

X-ray Structure Determination: X-ray quality crystals were selected in Fomblin YR-1800 perfluoroether (Alfa Aesar) at ambient temperature. The samples were cooled to 123(2) K during measurement. The data were collected on a Bruker D8 Quest diffractometer using Mo K_α radiation (λ = 0.71073 Å). The structures were solved by iterative methods (SHELXT)[10] and refined by full matrix least squares procedures (SHELXL).[11] Semi-empirical absorption corrections were applied (SADABS).[12] All non-hydrogen atoms were refined anisotropically, hydrogen atoms were included in the refinement at calculated positions using a riding model.

The reaction products **3Br** and **3*ⁱ*Pr** had to be crystallized relatively quickly, as longer reaction times led to decomposition of these adducts and formation of **4Br** or **4^{i}Pr** respectively. In the case of **3^{i}Pr** only small and twined crystals could be isolated. The diffraction pattern of these crystals could only be collected up to a low resolution. Therefore, this data is only used for structural proof and not discussed further.

Figure S1. Molecular structure of **3Br** in the single crystal. Ellipsoids are set at 50% probability (123 K). Color code: grey = carbon; white = hydrogen; blue = nitrogen, orange = phosphorus; brown = bromine.

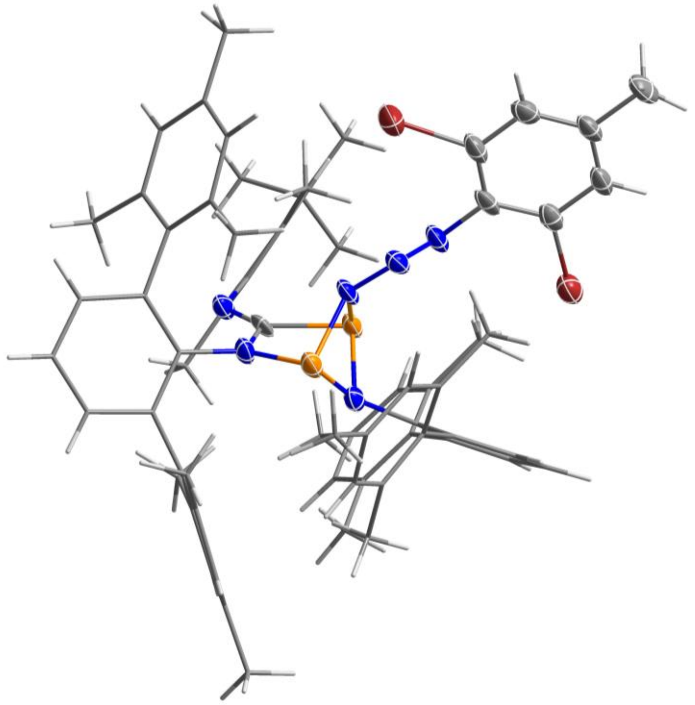

Figure S2. Molecular structure of **3^{i}Pr** in the single crystal. A-layer depicted for clarity. Color code: grey = carbon; white = hydrogen; blue = nitrogen, orange = phosphorus.

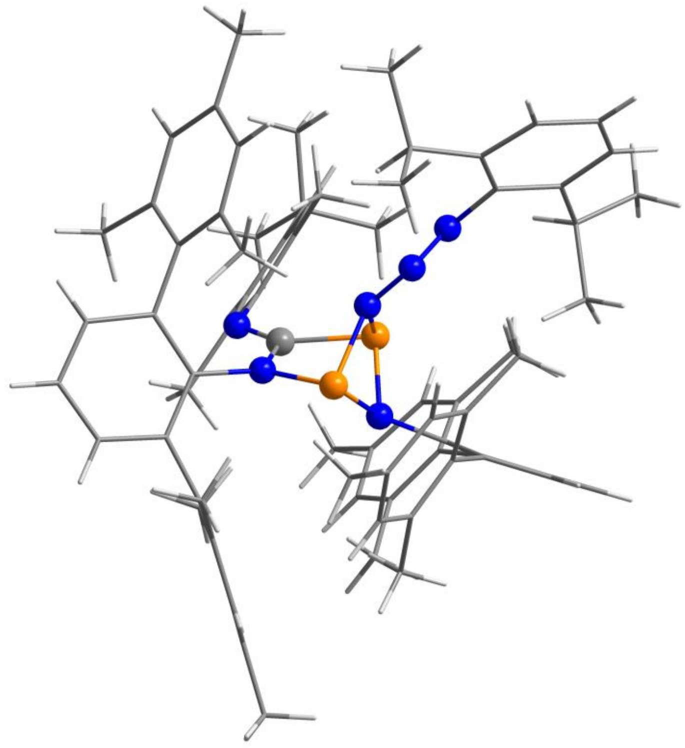

Table S2: Crystallographic details.

Compound	**3Br**	**3^{i}Pr**
Chem. Formula	$C_{68}H_{72}Br_2N_6P_2{\cdot}C_6H_{14}$	$C_{73}H_{84}N_6P_2{\cdot}C_6H_{14}$
Formula weight [g/mol]	1281.24	1193.61
Color	colorless	colorless
Crystal system	triclinic	triclinic
Space group	$P\bar{1}$	$P\bar{1}$
a [Å]	12.3368(19)	12.625(3)
b [Å]	16.186(2)	17.637(5)
c [Å]	17.445(3)	32.543(8)
α [°]	85.486(6)	98.806(9)
β [°]	87.556(7)	90.460(10)
γ [°]	72.176(6)	95.259(10)
V [Å^3]	3305.4(9)	7129(3)
Z	2	4
$\rho_{calcd.}$ [g/cm^3]	1.287	1.112
μ [mm^{-1}]	1.322	0.107
T [K]	123(2)	123(2)
Measured reflections	64978	29112
Independent reflections	9759	15823
Reflections with $I > 2\sigma(I)$	5791	10065
R_{int}	0.1781	0.0746
F(000)	1344	2376
$R_1(R[F^2>2\sigma(F^2)])$	0.055	0.0662
w$R_2(F^2)$	0.1433	0.1781
GooF	1.013	1.013
No. of Parameters	721	1595
CCDC #	2371934	

3 Syntheses of starting materials

3.1 [P(μ-NTer)]$_2$(MtpNC) (2)

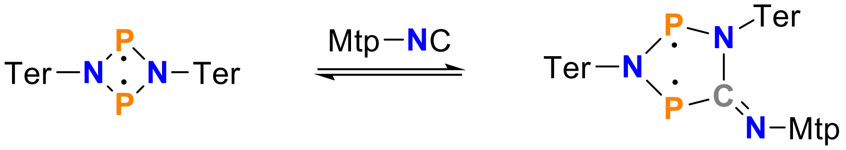

2 *was synthesized according to a literature procedure.*[6]

In an argon filled dry box one equivalent of [P(μ-NTer]$_2$ (0.22 g, 0.31 mmol) and one equivalent of MtpNC (0.057 g, 0.31 mmol) were combined in a vial and benzene (5 mL) was added. An immediate change of color from orange to dark blue was observed. After stirring the solution for 15 min the solvent was evaporated *in vacuo* (1×10^{-3} mbar, 50 °C, water bath) yielding product **2** as a dark blue solid. Yield: 0.23 g (0.25 mmol, 82 %). Immediate generation of the housane type species could be observed in the Raman spectrum (data indicated by asterisks).

Mp: 155 °C (decomposition). **EA:** calc. (found) in %: C 81.03 (80.06), H 7.47 (7.08), N 4.65 (4.56). **^{31}P{^{1}H} NMR** (298 K, C_6D_6, 202.46 MHz): δ = 222.7 (d, 1 P, $^{2}J(^{31}P,^{31}P)$ = 136 Hz, N*P*C); 258.7 (d, 1 P, $^{2}J(^{31}P,^{31}P)$ = 136 Hz, N*P*N). **^{1}H NMR** (298 K, C_6D_6, 500.13 MHz): δ = 1.28 (s, 9 H, *t*Bu-C*H*$_3$); 1.72 (s, 6 H, Mes *o*-C*H*$_3$); 1.74 (s, 6 H, Mes *o*-C*H*$_3$); 1.95 (s, 12 H, Mes *o*-C*H*$_3$); 2.27 (s, 6 H, C*H*$_3$); 2.29 (s, 6 H, C*H*$_3$); 2.30 (s, 6 H, C*H*$_3$); 6.72-7.10 (m, 16 H, Ph-C*H*). **^{13}C{^{1}H} NMR** (THF-d$_8$, 298 K, 125.77 MHz): δ = 19.3 (s, *C*H$_3$); 19.4 (s, *C*H$_3$); 21.3 (s, *C*H$_3$); 21.4 (s, *C*H$_3$); 21.6 (s, *C*H$_3$); 21.6 (s, *C*H$_3$); 21.7 (s, *C*H$_3$); 21.8 (s, *C*H$_3$); 32.2 (s, C-(*C*H$_3$)$_3$); 34.5 (s, *C*-(CH$_3$)$_3$); 125.6 (s, PhCH); 128.3 (s, PhCH); 128.4 (s, PhCH); 129.2 (s, PhCH); 129.3 (s, Ph*C*); 129.5 (s, PhCH); 130.3 (s, PhCH); 132.1 (s, PhCH); 132.9 (s, PhCH); 136.4 (s, Ph*C*); 137.0 (s, Ph*C*); 137.4 (s, Ph*C*); 137.6 (s, Ph*C*); 137.7 (s, Ph*C*); 138.0 (s, Ph*C*); 138.2 (s, Ph*C*); 139.8 (s, Ph*C*); 141.0 (d, Ph*C*, *J* = 4 Hz); 143.7 (s, Ph*C*); 149.2 (s, Ph*C*); 149.2 (s, Ph*C*). **IR** (ATR, 32 scans, cm^{-1}): $\tilde{\nu}$ = 3460 (w), 3439 (w), 3423 (w), 3408 (w), 3386 (w), 3361 (w), 3031 (w), 2994 (w), 2949 (m), 2914 (m), 2858 (w), 2728 (w),

1634 (w), 1611 (m), 1568 (w), 1519 (m), 1477 (m), 1447 (m), 1405 (m), 1374 (m), 1360 (m), 1302 (w), 1271 (m), 1247 (m), 1228 (m), 1193 (s), 1164 (m), 1144 (m), 1123 (m), 1082 (m), 1012 (m), 989 (m), 954 (m), 940 (m), 907 (m), 882 (m), 870 (m), 845 (s), 835 (m), 802 (s), 775 (m), 752 (s), 715 (m), 692 (m), 676 (s), 653 (m), 602 (m), 560 (m), 538 (m), 530 (m), 509 (m), 501 (m), 470 (m), 449 (m), 429 (m), 408 (m). **Raman*** (633 nm, 10 s, 10 scans, cm^{-1}): $\tilde{\nu}$ = 3048 (6), 3035 (1), 3019 (1), 3016 (1), 2989 (1), 2955 (2), 2916 (3), 2871 (1), 2860 (1), 2734 (1), 1691 (1), 1631 (5), 1604 (10), 1580 (3), 1529 (1), 1482 (2), 1477 (2), 1429 (2), 1394 (1), 1382 (2), 1308 (7), 1286 (1), 1267 (1), 1255 (2), 1240 (1), 1211 (1), 1187 (1), 1176 (1), 1167 (1), 1132 (1), 1113 (1), 1103 (1), 1088 (1), 1070 (2), 1008 (1), 959 (1), 949 (1), 922 (1), 809 (1), 748 (1), 743 (1), 716 (1), 696 (1), 667 (1), 656 (1), 631 (3), 600 (1), 594 (1), 579 (6), 563 (2), 555 (3), 545 (1), 525 (2), 514 (2), 505 (3), 499 (2), 468 (1), 449 (2), 427 (1), 415 (1), 393 (1), 370 (1), 348 (2), 312 (1), 272 (1), 243 (1), 238 (1), 120 (1), 93 (2), 78 (1).

Figure S3: NMR, IR and Raman spectra of [P(μ-NTer)]$_2$MtpNC **2** (solvent signals indicated by asterisks).

$^{31}P\{^1H\}$ NMR spectrum

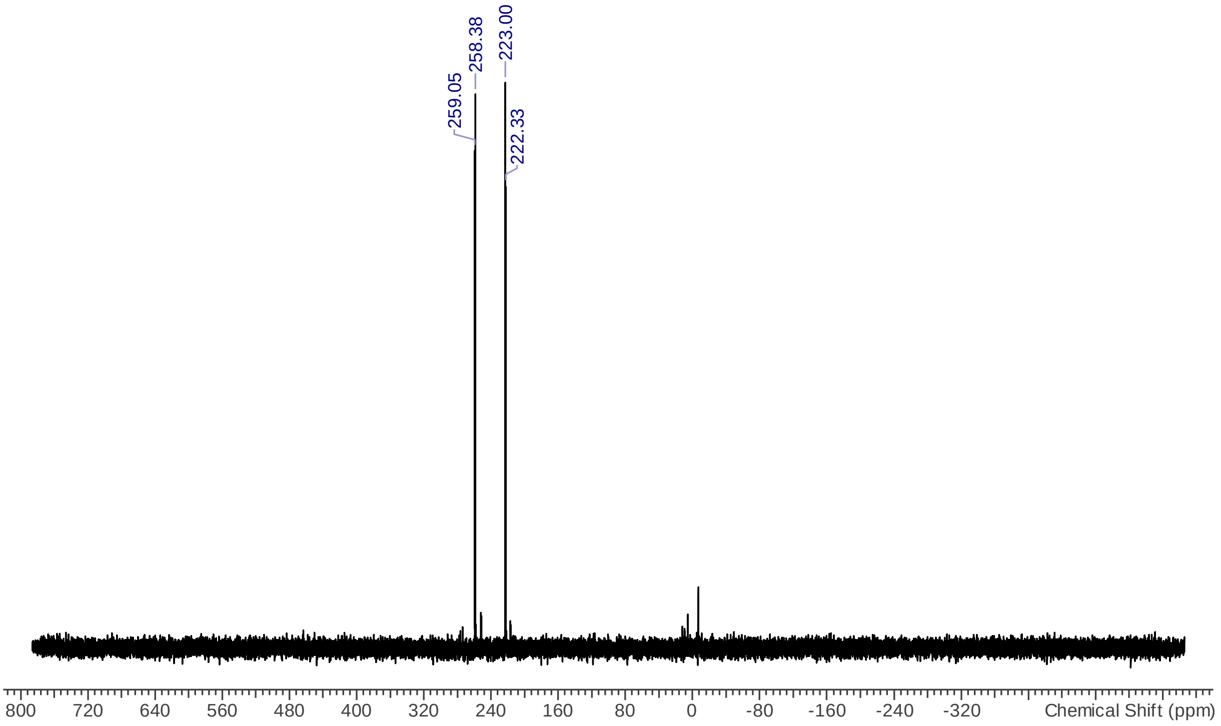

Figure S3 continued.

^{1}H NMR spectrum

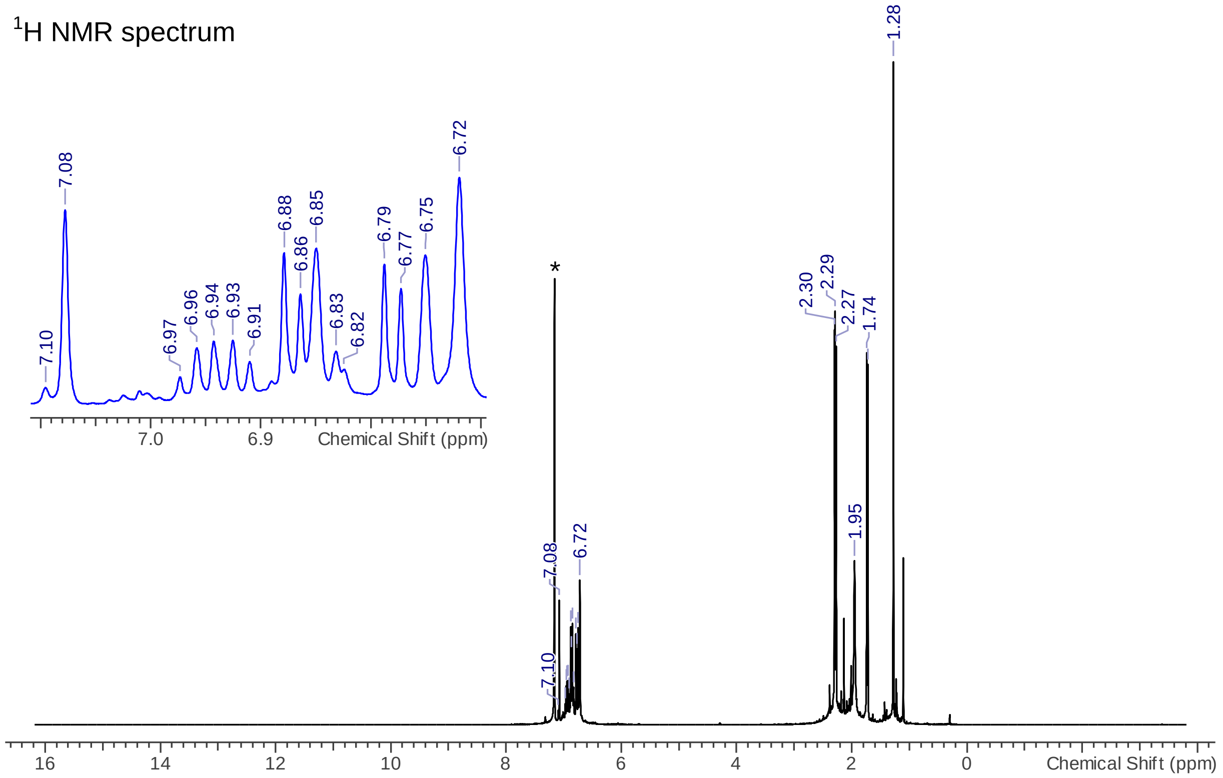

^{13}C{^{1}H} NMR spectrum

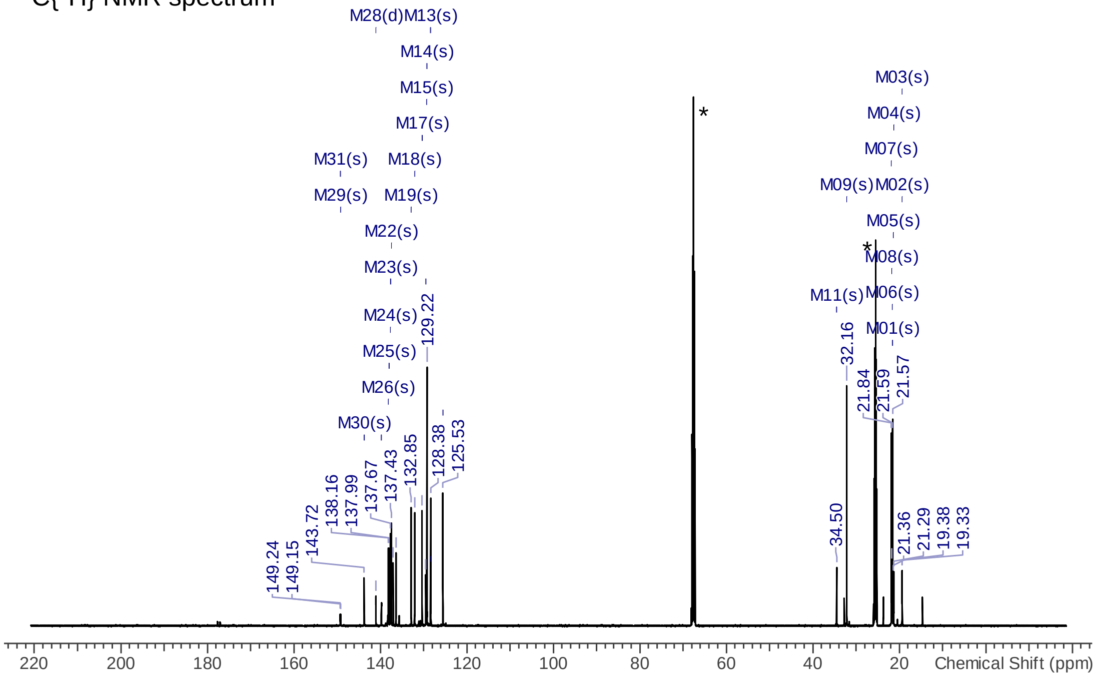

Figure S3 continued.

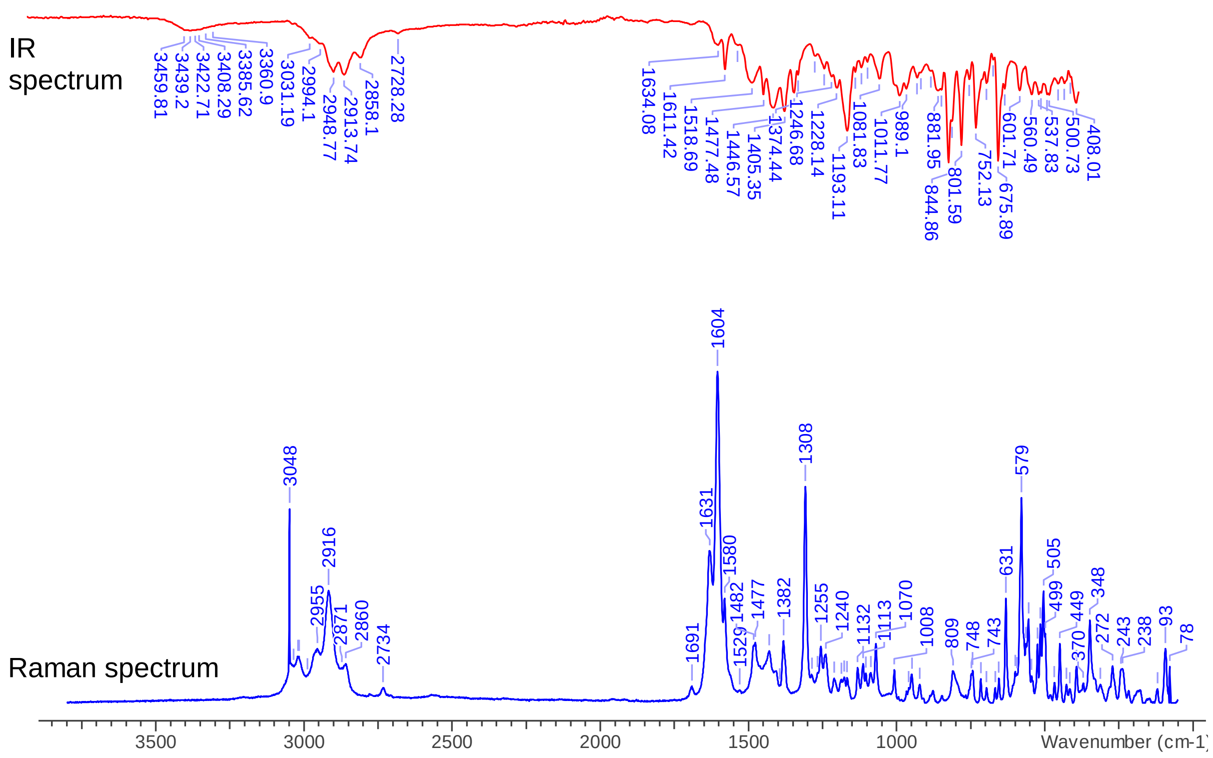

4 Syntheses of compounds

4.1 Synthesis of [P(μ-NTer)]$_2$(MtpNC)($C_7H_5Br_2N_3$) 3Br

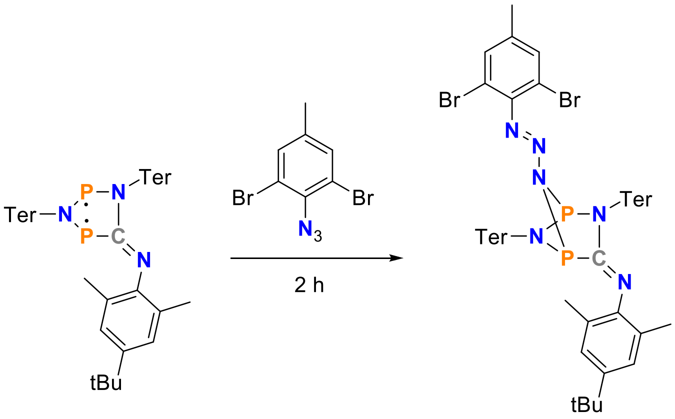

Five-membered biradical **2** (0.18 g, 0.20 mmol) was dissolved in 5 mL of benzene inside a glass vessel in an argon filled drybox. Subsequently, a solution of 2,6-dibromo-4-methylphenylazide (0.058 g, 0.20 mmol) in 2 mL of benzene was added and the mixture was stirred for 2 h with a glass stir bar, whereupon the color changed to an intense green. Afterwards, the solvent of the solution was removed *in vacuo* (1×10^{-3} mbar, 30 °C, water bath) and the solid residue was extracted with 10 mL of *n*-hexane followed by filtration through a syringe filter (0.2 µm pore size). The solvent of the filtrate was removed (1×10^{-3} mbar, 30 °C, water bath) until crystallization commences and the mixture was left undisturbed overnight (room temperature). Product **3Br** crystallized selectively from the mixture and was isolated by removal of the remaining amount of *n*-hexane solution. The crystals were washed with cold *n*-hexane (0 °C) for two times and the pure crystals were dried *in vacuo* (1×10^{-3} mbar, 30 °C, water bath) for 10 min yielding product **3Br** as a colorless solid. Yield: 0.126 g (0.106 mmol, 53%). Compound **3Br** should be stored in an argon filled drybox in solid state. Storage in solution is not possible as reaction to **4Br** commences.

$C_{68}H_{72}Br_2N_6P_2$ (1195 g/mol) **Mp**. 119.2 °C (decomp.). **CHN** calc. (found) in %: C 68.34 (68.71), H 6.07 (5.79), N 7.03 (6.70). **^{31}P{^{1}H} NMR** (−40 °C, THF-d_8, 202.5 MHz): δ = 184.5

(br. s, 1P, N*P*C), 214.8 (br. s, 1P, N*P*N). **^{1}H NMR** (−40 °C, THF-d_8, 500.1 MHz): δ = 1.30 (m, 6 H, C*H*$_3$); 1.36 (s, 9 H, *t*Bu-C*H*$_3$); 1.68 (br. s, 6 H, Mes *o*-C*H*$_3$); 1.82 (br. s, 6 H, Mes *o*-C*H*$_3$); 1.90-2.09 (m, 12 H, Mes *o*-C*H*$_3$); 2.22 (m, 3 H, $C_6H_2Br_2CH_3$); 2.27-2.38 (m, 12 H, C*H*$_3$); 6.46-7.10 (m, 16 H, Ph-C*H*); 7.57 (s, 2 H, $C_6H_2Br_2CH_3$). **^{13}C{^{1}H} NMR** (−40 °C, THF-d_8, 125.8 MHz): δ = 20.2 (s, *C*H$_3$); 20.6 (m, *C*H$_3$); 21.0 (s, *C*H$_3$); 21.2 (s, *C*H$_3$); 21.6 (m, *C*H$_3$); 22.4 (s, *C*H$_3$); 22.6 (m, *C*H$_3$); 23.9 (s, *C*H$_3$); 32.1 (s, *C*H$_3$); 32.2 (s, *C*H$_3$); 33.0 (s, C-(*C*H$_3$)$_3$); 34.9 (s, *C*-(CH$_3$)$_3$); 124.4 (s, Ph*C*H); 128.0 (m, Ph*C*H); 128.9 (s, Ph*C*H); 129.4 (s, Ph*C*H); 129.8 (s, Ph*C*); 130.1 (s, Ph*C*H); 130.6 (s, Ph*C*H); 132.1 (s, Ph*C*H); 132.2 (s, Ph*C*H); 134.5 (s, Ph*C*H); 135.5 (s, Ph*C*H); 136.2 (s, Ph*C*); 136.4 (s, Ph*C*); 136.6 (s, Ph*C*); 137.1 (s, Ph*C*); 137.5 (s, Ph*C*); 137.6 (br. s, Ph*C*); 137.8 (s, Ph*C*); 137.9 (s, Ph*C*); 138.0 (s, Ph*C*); 138.1 (s, Ph*C*); 139.7 (s, Ph*C*); 140.3 (m, Ph*C*); 141.4 (s, Ph*C*); 142.3 (s, Ph*C*); 143.1 (s, Ph*C*); 145.4 (s, Ph*C*). **IR** (ATR, 32 scans, cm^{-1}): $\tilde{\nu}$ = 2953 (w), 2916 (w), 2856 (w), 1632 (w), 1609 (w), 1535 (w), 1480 (w), 1453 (w), 1440 (w), 1401 (w), 1379 (w), 1358 (w), 1304 (m), 1228 (vs), 1201 (s), 1185 (s), 1119 (s), 979 (s), 923 (w), 890 (w), 874 (w), 853 (w), 802 (m), 754 (m), 742 (m), 723 (w), 709 (w), 692 (w), 649 (w), 618 (w), 610 (w), 602 (w), 587 (w), 575 (w), 552 (w), 523 (m), 492 (w), 476 (w), 462 (w), 449 (w), 414 (w). **Raman** (633 nm, 10 s, 10 scans, cm^{-1}): $\tilde{\nu}$ = 3052 (2), 3019 (2), 2964 (2), 2956 (3), 2922 (7), 2878 (2), 2862 (2), 1638 (2), 1614 (4), 1606 (3), 1593 (6), 1485 (2), 1475 (2), 1471 (1), 1446 (7), 1406 (9), 1396 (10), 1383 (8), 1358 (1), 1308 (8), 1287 (2), 1278 (1), 1263 (2), 1251 (2), 1236 (5), 1223 (2), 1213 (1), 1210 (1), 1206 (1), 1130 (2), 1077 (3), 950 (1), 808 (3), 797 (2), 758 (2), 748 (3), 745 (3), 713 (1), 623 (1), 605 (1), 583 (5), 567 (3), 554 (3), 543 (2), 528 (2), 517 (2), 504 (7), 481 (3), 466 (3), 421 (2), 408 (2), 360 (2), 327 (9), 305 (4), 293 (2), 263 (2), 257 (2), 244 (3), 218 (1), 197 (2), 120 (2), 91 (1). **MS** (EI, 70 eV, m/z): 282 (11); 292 (27, $[C_7H_5Br_2N_3+H]^+$); 294 (70); 295 (12); 296 (47); 297 (16); 310 (64); 311 (21); 312 (20, [Ter−H]$^+$); 313 (16, [Ter]$^+$); 314 (10); 324 (17); 326 (63, [TerN−H]$^+$); 327 (25, [TerN]$^+$); 328 (69, [TerN+H]$^+$); 329 (30); 330 (75); 342 (44); 343 (13); 344 (15); 356 (13); 357 (18, [TerNP−H]$^+$); 358 (100, [TerNP]$^+$); 359 (73, [TerNP+H]$^+$); 360 (10); 651 (11); 715 (97); 716 (87); 717 (32); 1178 (1, [M−CH$_3$−H]$^+$).

Figure S4: NMR, IR and Raman spectra of **3Br** (solvent signals indicated by asterisks).

$^{31}P\{^1H\}$ NMR spectrum

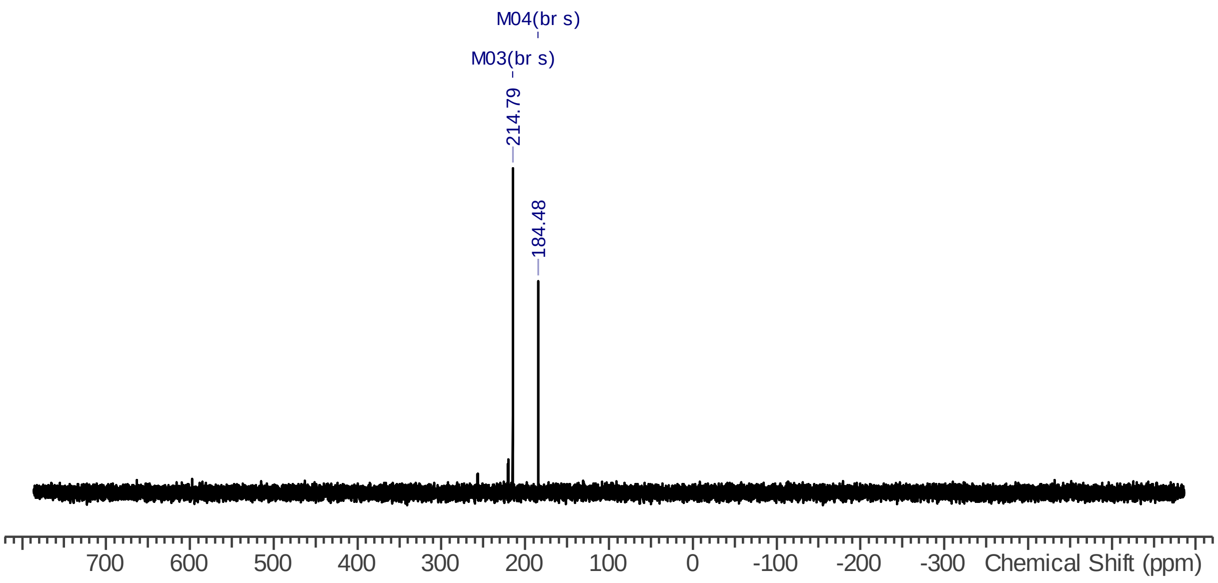

^1H NMR spectrum

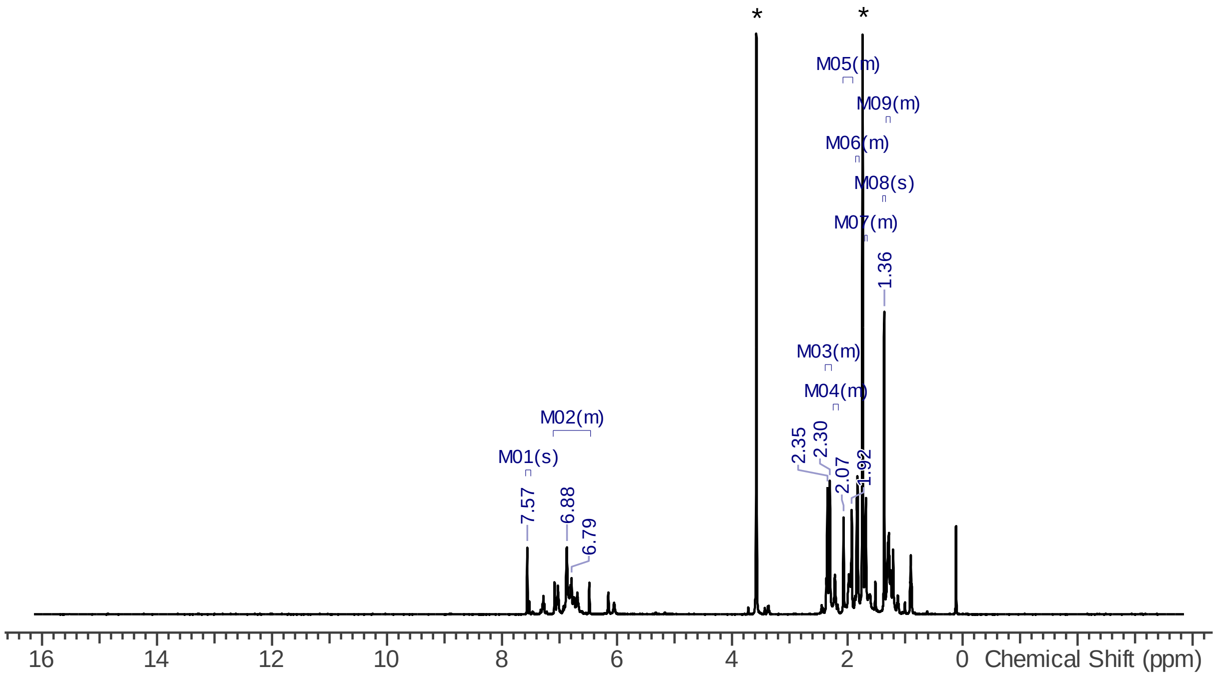

Figure S4 continued.

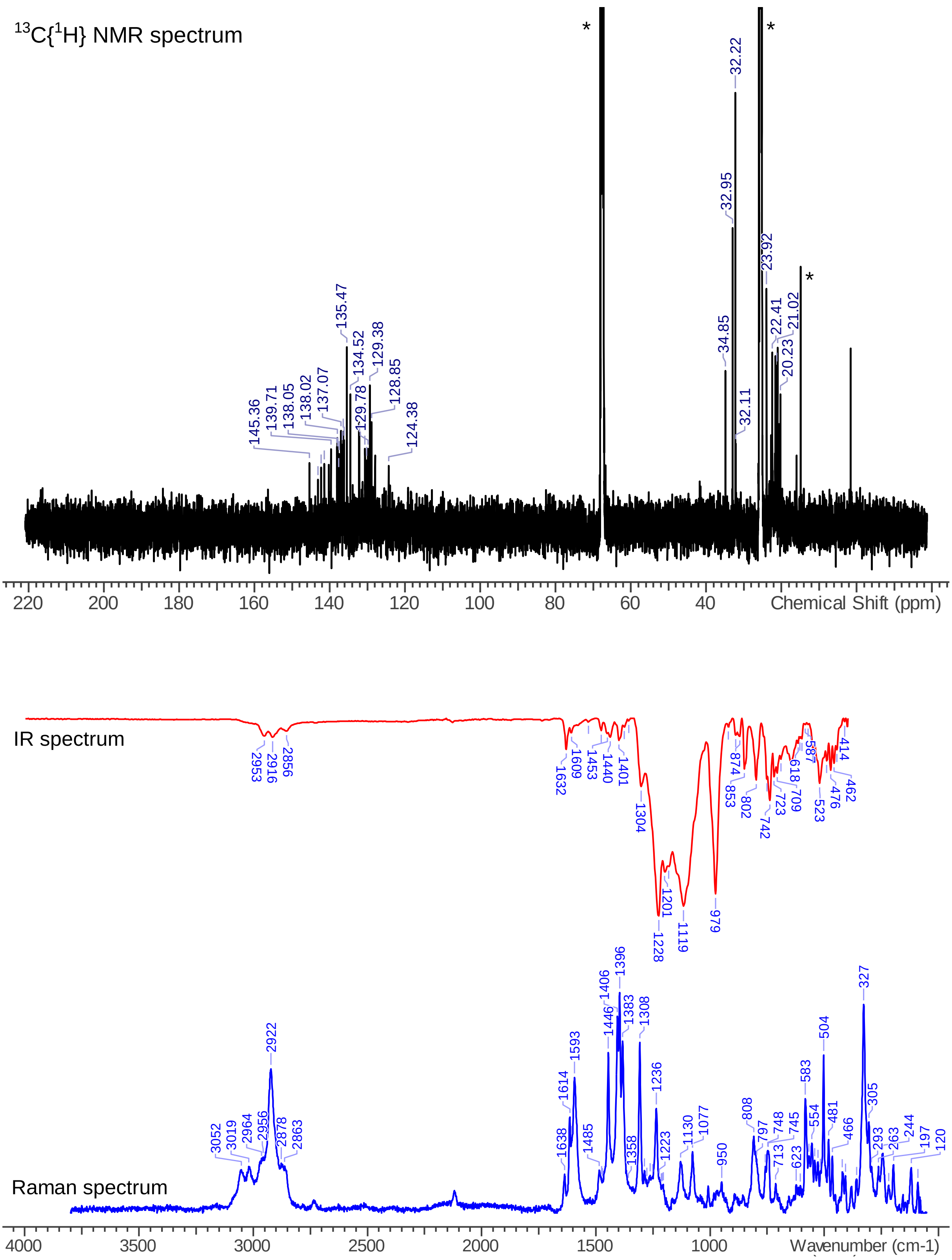

4.2 Synthesis of [P(μ-NTer)]$_2$(MtpNC)(Ph(^{i}Pr)$_2$N$_3$) 3^{i}Pr

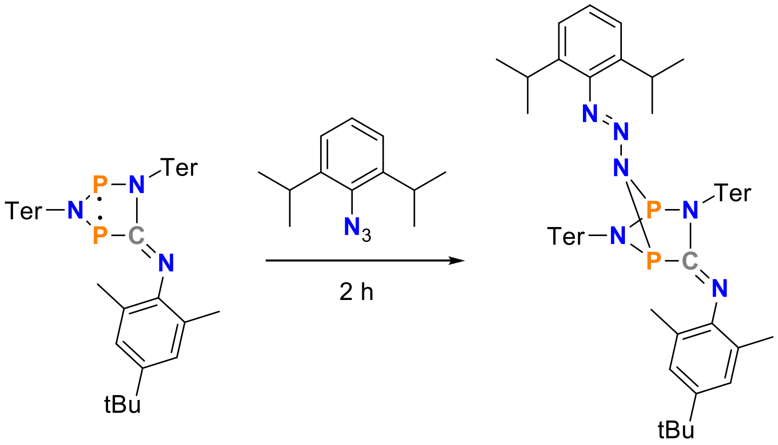

Five-membered biradical **2** (0.18 g, 0.20 mmol) was dissolved in 5 mL of benzene inside a glass vessel in an argon filled drybox. Subsequently, 2,6-diisopropylphenylazide (0.041 g, 0.20 mmol) was added and the mixture was stirred for 2 h with a glass stir bar, whereupon the color changed to an intense green. Afterwards, the solvent of the solution was removed *in vacuo* (1×10^{-3} mbar, 30 °C, water bath) and the solid residue was extracted with 10 mL of *n*-hexane followed by filtration through a syringe filter (0.2 μm pore size). The solvent of the filtrate was removed (1×10^{-3} mbar, 30 °C, water bath) until crystallization commences and the mixture was left undisturbed overnight (room temperature). Product **3^{i}Pr** crystallized selectively from the mixture and was isolated by removal of the remaining amount of *n*-hexane solution. The crystals were washed with cold *n*-hexane (0 °C) for two times and the pure crystals were dried *in vacuo* (1×10^{-3} mbar, 30 °C, water bath) for 10 min yielding product **3^{i}Pr** as a colorless solid. Yield: 0.088 g (0.079 mmol, 40%). Compound **3^{i}Pr** should be stored in an argon filled drybox in solid state. Storage in solution is not possible as reaction to **4^{i}Pr** commences.

$C_{73}H_{84}N_6P_2$ (1107 g/mol) **Mp**. 119.0 °C (decomp.). **CHN** calc. (found) in %: C 79.17 (78.83), H 7.65 (8.13), N 7.59 (7.44). **^{31}P{^{1}H} NMR** (−40 °C, THF-d_8, 161.8 MHz): δ = 179.9 (br. s, 1P, N*P*C), 216.1 (br. s, 1P, N*P*N). **^{1}H NMR** (−40 °C, THF-d_8, 399.8 MHz): δ = 1.01-1.07 (m, 6 H, C*H*$_3$); 1.09 (s, 3 H, Ter-C*H*$_3$); 1.17-1.19 (m, 12 H, *i*Pr-C*H*$_3$); 1.35 (s, 9 H, *t*Bu-C*H*$_3$); 1.53 (s, 3 H, Ter-C*H*$_3$); 1.63 (s, 3 H, Ter-C*H*$_3$); 1.80 (s, 3 H, Ter-C*H*$_3$); 1.88 (s, 3

H, Ter-C*H*$_3$); 1.90 (s, 3 H, Ter-C*H*$_3$); 2.01 (s, 3 H, Ter-C*H*$_3$); 2.07 (s, 3 H, Ter-C*H*$_3$); 2.15 (s, 3 H, Ter-C*H*$_3$); 2.22 (s, 3 H, Ter-C*H*$_3$); 2.24 (s, 3 H, Ter-C*H*$_3$); 2.29 (s, 3 H, Ter-C*H*$_3$); 3.00 (sept, 2 H, *i*Pr-C*H*); 6.52-6.97 (m, 14 H, Ph-C*H*); 7.11-7.18 (m, 4 H, Ph-C*H*); 7.33 (t, 1 H, Dipp-*p*-*H*). **^{13}C{^{1}H} NMR** (−40 °C, THF-*d*$_8$, 100.5 MHz): δ = 17.1 (s, *C*H$_3$); 17.4 (s, *C*H$_3$); 20.5 (s, *C*H$_3$); 21.1 (s, *C*H$_3$); 21.2 (s, *C*H$_3$); 21.3 (s, *C*H$_3$); 21.4 (s, *C*H$_3$); 21.5 (s, *C*H$_3$); 21.8 (s, *C*H$_3$); 21.9 (s, *C*H$_3$); 22.1 (s, *C*H$_3$); 22.2 (s, *C*H$_3$); 23.0 (s, *C*H$_3$); 23.1 (s, *C*H$_3$); 23.8 (br. s, *C*H$_3$); 23.9 (s, *i*Pr-*C*H$_3$); 26.2 (s, *C*H$_3$); 28.3 (s, *C*H$_3$); 29.7 (s, *i*Pr-*C*H); 31.4 (s, *C*H$_3$); 32.1 (s, C-(*C*H$_3$)$_3$); 34.7 (s, *C*-(CH$_3$)$_3$); 123.5 (s, Ph*C*H); 124.2 (s, Ph*C*H); 124.9 (s, Ph*C*H); 125.1 (s, Ph*C*H); 125.7 (s, Ph*C*H); 126.1 (s, Ph*C*H); 127.2 (s, Ph*C*H); 127.7 (s, Ph*C*H); 127.9 (s, Ph*C*H); 128.1 (s, Ph*C*H); 128.6 (s, Ph*C*H); 129.1 (s, Ph*C*H); 129.3 (br. s, Ph*C*H); 129.4 (br. s, Ph*C*H); 129.8 (s, Ph*C*); 130.3 (s, Ph*C*H); 130.9 (s, Ph*C*H); 131.0 (s, Ph*C*H); 131.5 (s, Ph*C*H); 131.7 (s, Ph*C*H); 131.9 (s, Ph*C*H); 132.7 (s, Ph*C*H); 132.9 (s, Ph*C*H); 134.4 (m, Ph*C*H); 135.6 (m, Ph*C*H); 135.7 (s, Ph*C*); 136.2 (m, Ph*C*); 136.7 (m, Ph*C*); 136.9 (s, Ph*C*); 137.2 (s, Ph*C*); 137.4 (s, Ph*C*); 137.6 (s, Ph*C*); 137.8 (m, Ph*C*); 137.9 (s, Ph*C*); 138.1 (m, Ph*C*); 138.4 (s, Ph*C*); 138.6 (s, Ph*C*); 139.8 (m, Ph*C*); 140.4 (s, Ph*C*); 141.6 (m, Ph*C*); 141.8 (s, Ph*C*); 142.7 (m, Ph*C*); 143.6 (s, Ph*C*); 144.9 (s, Ph*C*); 145.2 (s, Ph*C*); 145.9 (m, Ph*C*); 165.0 (m, N=*C*). **IR** (ATR, 32 scans, cm^{-1}): $\tilde{\nu}$ = 2959 (m), 2918 (m), 2864 (m), 1634 (m), 1611 (m), 1578 (w), 1480 (m), 1455 (m), 1447 (m), 1422 (vs), 1399 (m), 1379 (m), 1360 (m), 1319 (w), 1273 (w), 1249 (m), 1236 (m), 1208 (m), 1164 (w), 1113 (m), 1092 (m), 1072 (m), 1030 (m), 1006 (w), 977 (m), 968 (m), 923 (w), 878 (m), 847 (s), 802 (m), 791 (m), 773 (w), 756 (s), 744 (m), 727 (m), 711 (w), 694 (w), 649 (w), 620 (w), 610 (w), 538 (w), 523 (w), 507 (w), 488 (m), 476 (m), 455 (m). **MS** (EI, 70 eV, m/z): 98 (17); 122 (20); 126 (42); 130 (14); 145 (12); 168 (18); 187 (41, [MtpNC]$^+$); 189 (13); 190 (13); 205 (11); 208 (52); 224 (20); 225 (11); 227 (23); 228 (12); 240 (43); 241 (28); 243 (54); 244 (64); 245 (28); 246 (59); 248 (10); 255 (16); 257 (34); 258 (22); 259 (53); 260 (31); 262 (81); 263 (45); 264 (26); 265 (19); 271 (18); 272 (12); 273 (43); 274 (26); 275 (19); 276 (79); 277 (58); 279 (40); 280 (40); 281 (29); 282 (14); 283 (20); 290 (27); 292 (25); 293 (18); 294 (11); 295 (84); 296 (33); 297 (92, [Ter−Me−H]$^+$); 299 (86, [Ter−Me+H]$^+$); 300 (48); 309 (72); 310 (18); 311 (19); 312 (23, [Ter−H]$^+$); 315 (63); 316 (47); 326 (57, [TerN−H]$^+$); 327 (13, [TerN]$^+$); 329 (19); 425 (21);

426 (14); 427 (12); 439 (10); 440 (29); 441 (32); 442 (60); 443 (12); 453 (14); 455 (44); 456 (38); 457 (84, [M−DippN−Mtp−Ter]$^+$); 458 (33); 471 (41, [M−Dipp−Mtp−Ter]$^+$); 472 (100, [M−Dipp−Mtp−Ter+H]$^+$); 473 (74); 474 (15); 1106 (0.1, [M−H]$^+$).

Figure S5: NMR and IR spectra of **3^{i}Pr** (solvent signals indicated by asterisks).

$^{31}P\{^1H\}$ NMR spectrum

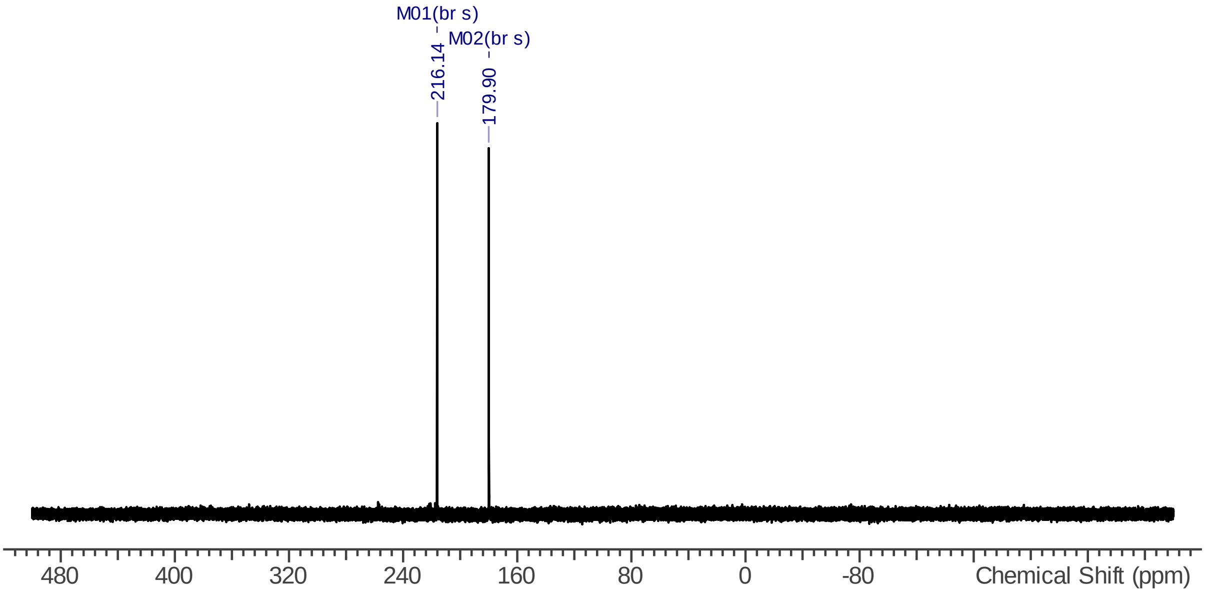

^1H NMR spectrum

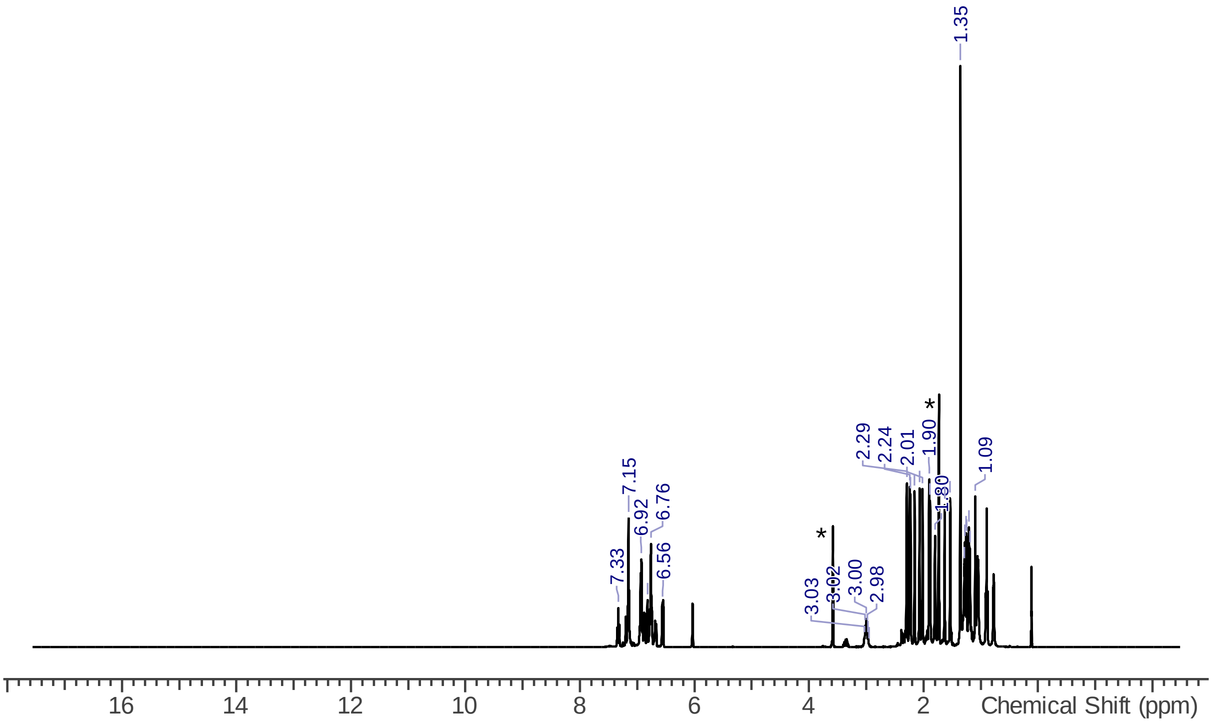

Figure S5 continued.

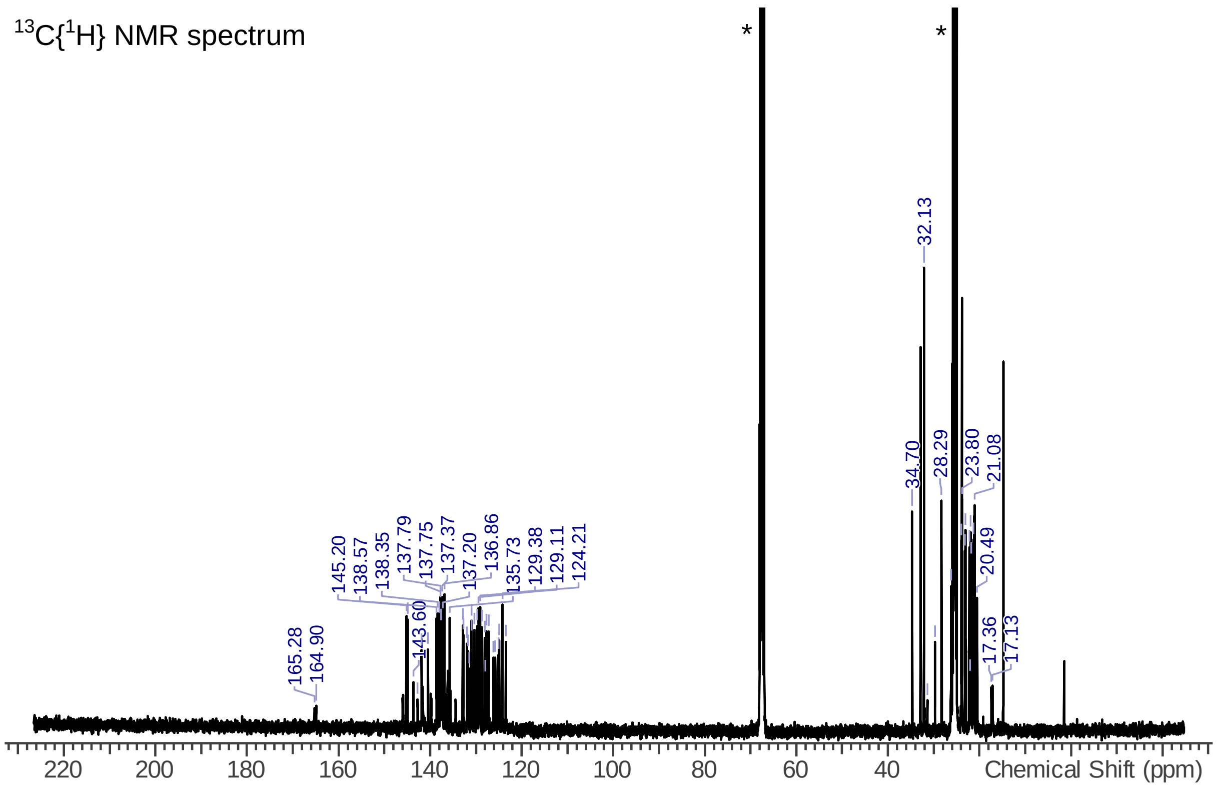

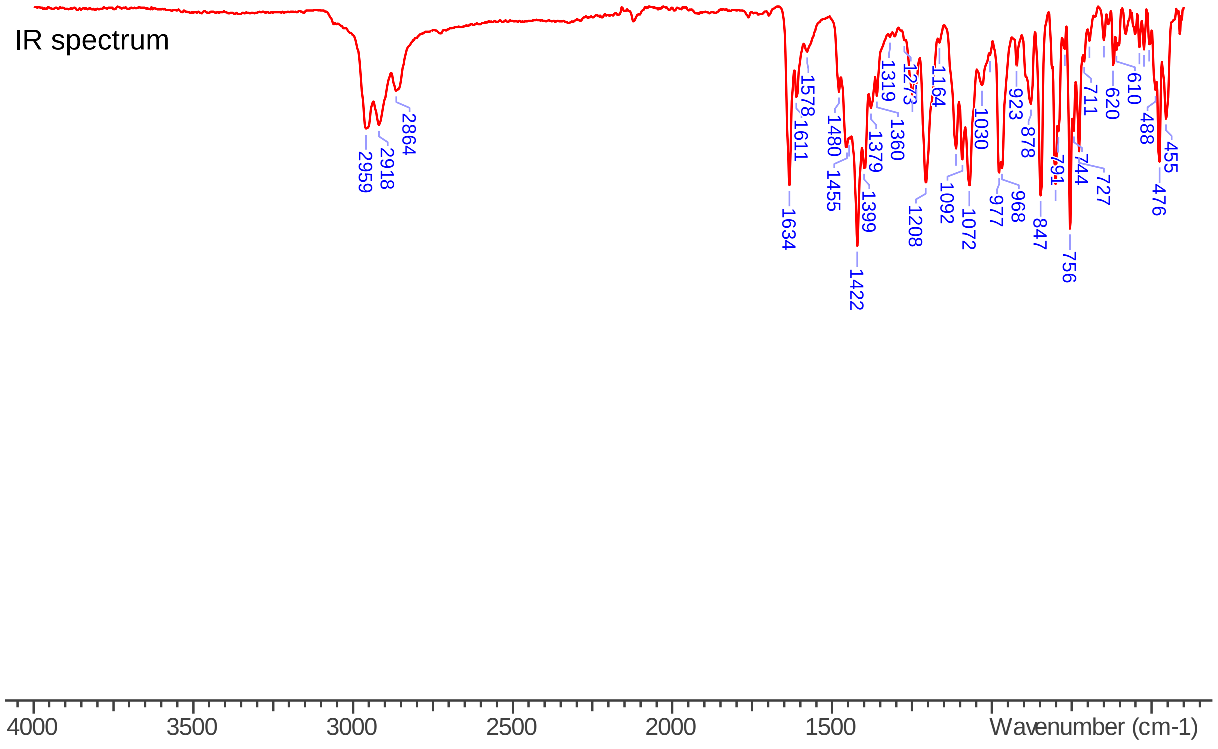

4.3 Generation of TerNPN(PhMeBr2)PNTer 4Br

Method A:

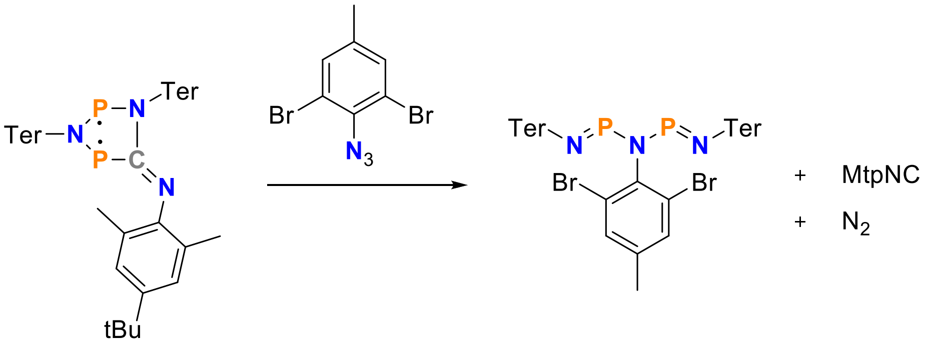

Five-membered biradical **2** (0.18 g, 0.20 mmol) was dissolved in 5 mL of benzene inside a glass vessel in an argon filled drybox. Subsequently, a solution of 2,6-dibromo-4-methylphenylazide (0.058 g, 0.20 mmol) in 2 mL of benzene was added and the mixture was stirred for 4 days with a glass stir bar, whereupon the color changed to an intense green. Afterwards, the solvent of the solution was removed *in vacuo* (1×10^{-3} mbar, 50 °C, water bath) and the solid residue was dried *in vacuo* (1×10^{-3} mbar, 50 °C, water bath) for 10 min. The remaining solid still contains impurities of five-membered biradical **2** and MtpNC alongside the desired product **4Br**. For isolation of pure compound **4Br** please refer to **Method B**.

Method B:

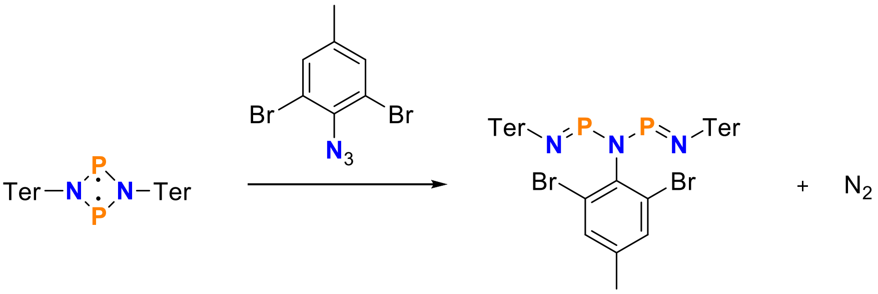

Biradical (**1**) [P(μ-NTer)]2 (0.060 g, 0.084 mmol) and 2,6-dibromo-4-methylphenylazide (0.024 g, 0.084 mmol) were added together in a glass vessel inside an argon filled drybox and dissolved in 3 mL of benzene. The color of the solution remained red but

an immediate evolution of gas was observable. Afterwards, the solvent of the solution was removed *in vacuo* (1×10^{-3} mbar, 50 °C, water bath) and the solid residue was dried *in vacuo* (1×10^{-3} mbar, 50 °C, water bath) for 10 min yielding product **4Br** as a red solid. Yield: 0.074 g (0.076 mmol, 90%).

$C_{55}H_{55}Br_2N_3P_2$ (980 g/mol) **Mp**. 93.3 °C (decomp.). **CHN** calc. (found) in %: C 67.42 (67.04), H 5.66 (5.68), N 4.29 (4.31). **$^{31}P\{^1H\}$ NMR** (25 °C, C_6D_6, 161.8 MHz): δ = 293.8 (br. s, 2P, N*P*N). **^1H NMR** (25 °C, C_6D_6, 399.8 MHz): δ = 1.85 (s, 3 H, $C_6H_2Br_2CH_3$); 1.97 (s, 24 H, Mes *o*-C*H*3); 2.29 (s, 12 H, Mes *p*-C*H*3); 6.76-6.81 (m, 10 H, Ph-C*H*); 6.91-6.97 (m, 6 H, Ph-C*H*). **$^{13}C\{^1H\}$ NMR** (25 °C, C_6D_6, 100.5 MHz): δ = 20.7 (s, *C*H3); 21.1 (m, *C*H3); 21.8 (s, *C*H3); 124.0 (s, Ph*C*); 124.5 (s, Ph*C*H); 128.8 (s, Ph*C*H); 129.2 (s, Ph*C*H); 129.4 (s, Ph*C*H); 132.3 (br. s, Ph*C*); 132.6 (s, Ph*C*H); 134.7 (s, Ph*C*); 136.6 (s, Ph*C*); 136.9 (s, Ph*C*); 138.0 (s, Ph*C*); 138.9 (s, Ph*C*); 143.9 (s, Ph*C*). **IR** (ATR, 32 scans, cm^{-1}): $\tilde{\nu}$ = 2997 (w), 2962 (w), 2943 (w), 2912 (w), 2875 (w), 2853 (w), 1612 (w), 1579 (w), 1482 (w), 1449 (m), 1437 (m), 1412 (m), 1375 (m), 1346 (w), 1292 (w), 1266 (w), 1245 (m), 1222 (w), 1191 (w), 1088 (w), 1031 (m), 1008 (w), 932 (w), 892 (m), 847 (vs), 800 (m), 754 (m), 740 (m), 697 (m), 676 (m), 651 (m), 618 (m), 600 (m), 557 (m), 524 (m), 509 (m), 499 (m), 493 (m), 460 (w), 439 (w), 416 (m). **MS** (EI, 70 eV, m/z): 133 (11); 134 (20); 140 (12); 141 (24); 142 (20); 148 (16); 149 (19); 150 (13); 156 (13); 157 (16); 164 (13); 165 (16); 263 (11); 265 (21); 267 (23); 268 (11); 281 (16); 282 (25); 283 (17, $[Ter-Me_2]^+$); 284 (10); 294 (21); 295 (13); 296 (34); 297 (33, $[Ter-Me-H]^+$); 298 (26, $[Ter-Me]^+$); 299 (31, $[Ter-Me+H]^+$); 309 (12); 310 (65); 311 (29); 312 (54, $[Ter-H]^+$); 313 (56, $[Ter]^+$); 314 (80, $[Ter+H]^+$); 315 (20); 324 (13); 326 (64, $[TerN-H]^+$); 327 (31, $[TerN]^+$); 328 (77, $[TerN+H]^+$); 329 (100); 330 (79); 342 (54); 343 (17, $[TerNP-Me]^+$); 344 (33, $[TerNP-Me+H]^+$); 357 (33, $[TerNP-H]^+$); 358 (48, $[TerNP]^+$); 359 (63, $[TerNP+H]^+$); 360 (19); 375 (21); 670 (11); 671 (44); 672 (18); 980 (0.2, $[M]^+$).

Figure S6: NMR and IR spectra of **4Br** (solvent signals indicated by asterisks).

$^{31}P\{^{1}H\}$ NMR spectrum

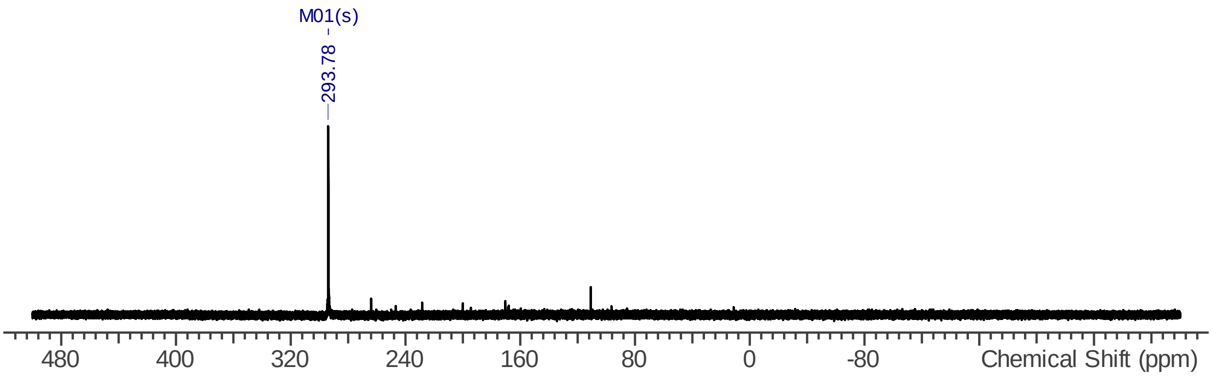

^{1}H NMR spectrum

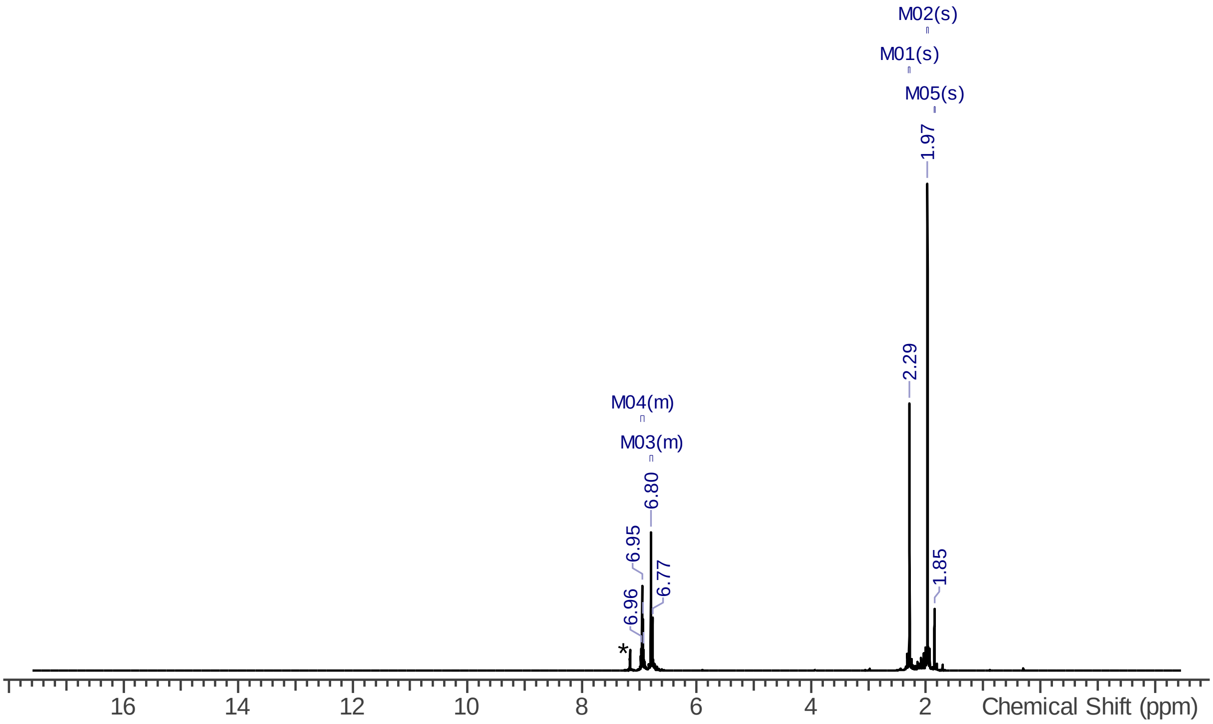

Figure S6 continued.

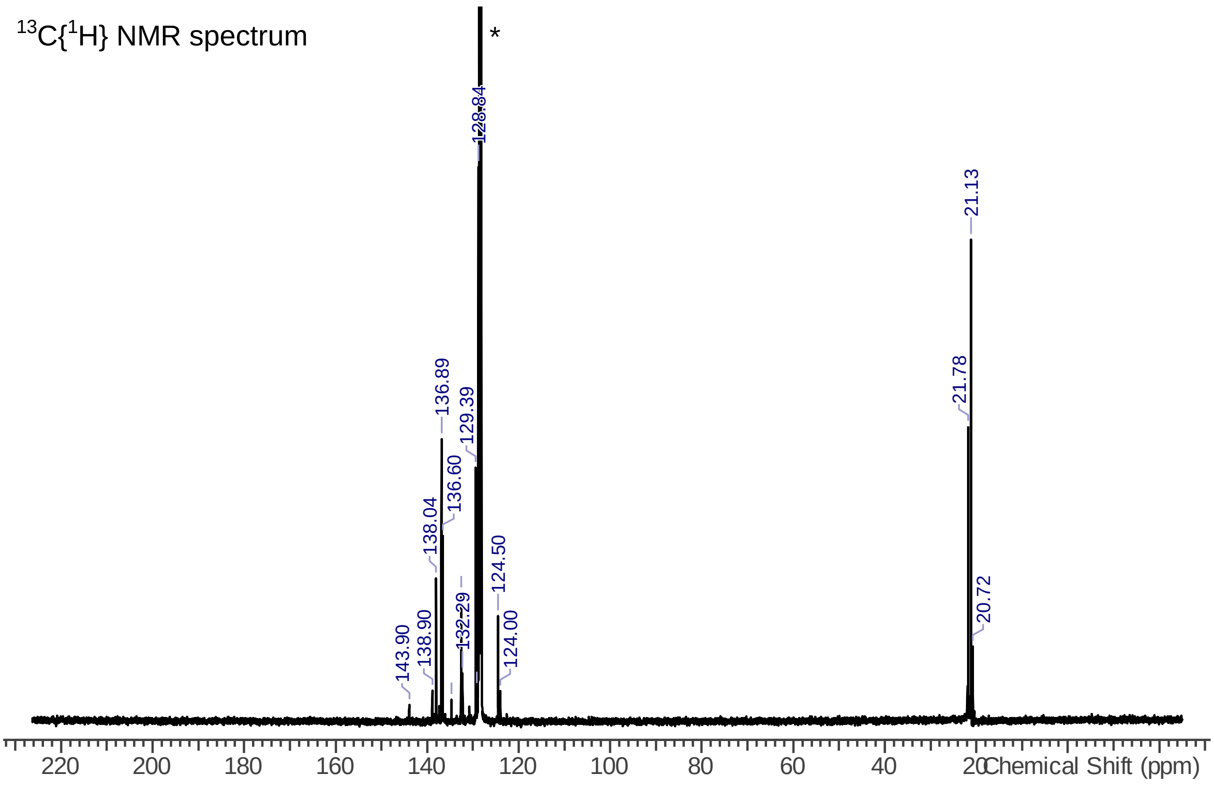

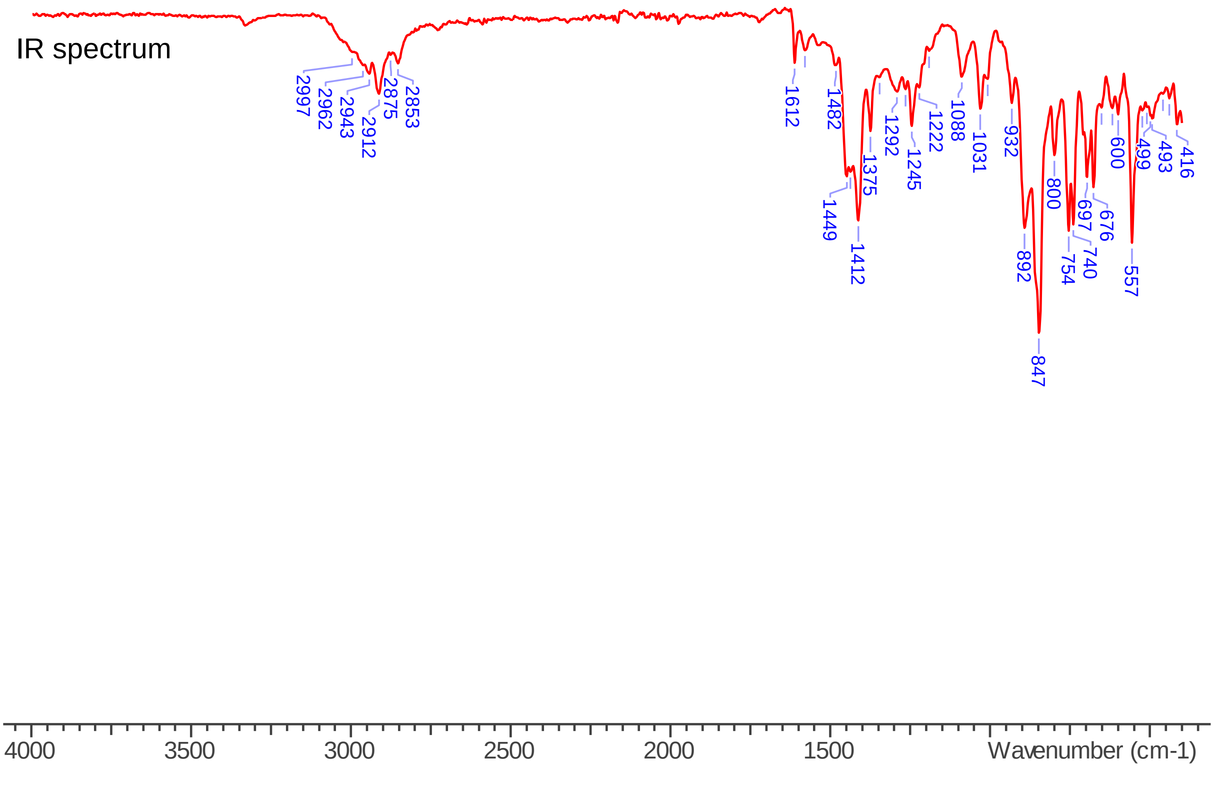

4.4 Generation of TerNPN(Ph(^{i}Pr)$_2$)PNTer 4^{i}Pr

Method A:

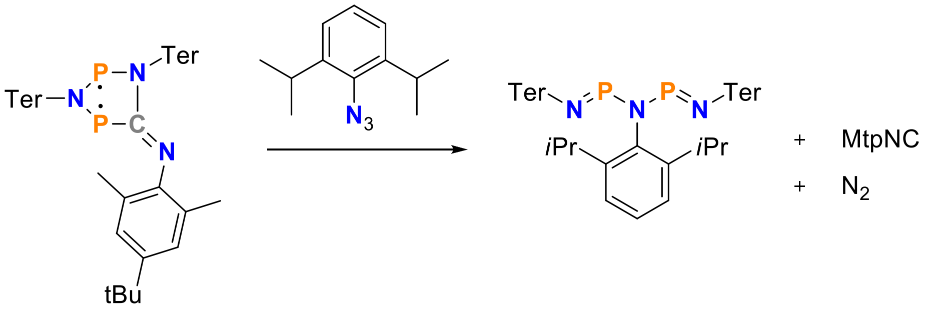

Five-membered biradical **2** (0.18 g, 0.20 mmol) was dissolved in 5 mL of benzene inside a glass vessel in an argon filled drybox. Subsequently, 2,6-diisopropylphenylazide (0.041 g, 0.20 mmol) was added and the mixture was stirred for 4 days with a glass stir bar, whereupon the color changed to an intense green. Afterwards, the solvent of the solution was removed *in vacuo* (1×10^{-3} mbar, 50 °C, water bath) and the solid residue was dried *in vacuo* (1×10^{-3} mbar, 50 °C, water bath) for 10 min. The remaining solid still contains impurities of five-membered biradical **2** and MtpNC alongside the desired product **4^{i}Pr**. For isolation of pure compound **4^{i}Pr** please refer to **Method B**.

Method B:

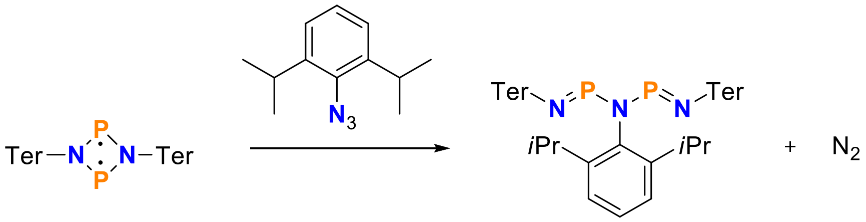

Biradical (**1**) [P(μ-NTer)]$_2$ (0.060 g, 0.084 mmol) and 2,6-diisopropylphenylazide (0.017 g, 0.084 mmol) were added together in a glass vessel inside an argon filled drybox and dissolved in 3 mL of benzene. The color of the solution remained red but an immediate evolution of gas was observable. Afterwards, the solvent of the solution was removed *in vacuo* (1×10^{-3} mbar, 50 °C, water bath) and the solid residue was dried

in vacuo (1×10^{-3} mbar, 50 °C, water bath) for 10 min yielding product **4^{i}Pr** as a red solid. Yield: 0.072 g (0.081 mmol, 96%).

$C_{60}H_{67}N_3P_2$ (892 g/mol) **Mp**. 76.6 °C (decomp.). **CHN** calc. (found) in %: C 80.78 (79.68), H 7.57 (7.49), N 4.71 (4.93). **^{31}P{^{1}H} NMR** (25 °C, C_6D_6, 161.8 MHz): δ = 297.4 (br. s, 2P, N*P*N). **^{1}H NMR** (25 °C, C_6D_6, 399.8 MHz): δ = 0.68-0.79 (m, 12 H, *i*Pr-C*H*$_3$); 1.12 (m, 2 H, *i*Pr-C*H*); 2.02 (s, 24 H, Mes *o*-C*H*$_3$); 2.26 (s, 12 H, Mes *p*-C*H*$_3$); 6.76-6.87 (m, 10 H, Ph-C*H*); 6.91-7.04 (m, 7 H, Ph-C*H*). **^{13}C{^{1}H} NMR** (25 °C, C_6D_6, 100.5 MHz): δ = 21.4 (s, *C*H$_3$); 21.7 (s, *C*H$_3$); 23.9 (s, *C*H$_3$); 24.7 (s, *i*Pr-*C*H$_3$); 29.6 (s, *i*Pr-*C*H); 124.1 (s, Ph*C*H); 124.2 (s, Ph*C*H); 124.6 (s, Ph*C*); 124.7 (s, Ph*C*); 129.7 (s, Ph*C*H); 132.2 (s, Ph*C*); 136.5 (s, Ph*C*); 136.8 (s, Ph*C*); 138.2 (s, Ph*C*); 146.6 (s, Ph*C*). **IR** (ATR, 32 scans, cm^{-1}): $\tilde{\nu}$ = 2997 (w), 2960 (m), 2914 (m), 1612 (w), 1577 (w), 1486 (w), 1455 (m), 1435 (m), 1408 (m), 1375 (m), 1362 (m), 1282 (m), 1253 (m), 1241 (m), 1210 (m), 1175 (m), 1109 (w), 1090 (m), 1031 (w), 1012 (w), 915 (m), 888 (s), 847 (vs), 791 (s), 754 (s), 738 (m), 715 (m), 676 (w), 653 (w), 620 (w), 600 (w), 565 (w), 524 (m), 431 (w). **MS** (EI, 70 eV, m/z): 128 (12); 133 (13); 134 (27); 135 (12); 140 (14); 141 (31); 142 (30); 148 (20); 149 (26); 150 (19); 156 (15); 157 (22); 162 (39, [Dipp+H]$^+$); 164 (16); 165 (23); 169 (11); 176 (11); 177 (18); 190 (14); 206 (26); 208 (11); 267 (15); 268 (15); 269 (12); 280 (10); 281 (19); 282 (32); 283 (23, [Ter−Me$_2$]$^+$); 284 (15); 295 (13); 296 (39); 297 (42, [Ter−Me−H]$^+$); 298 (35, [Ter−Me]$^+$); 299 (48, [Ter−Me+H]$^+$); 300 (13); 309 (11); 310 (54); 311 (27); 312 (67, [Ter−H]$^+$); 313 (69, [Ter]$^+$); 314 (95, [Ter+H]$^+$); 315 (29); 324 (12); 326 (48, [TerN−H]$^+$); 327 (26, [TerN]$^+$); 328 (89, [TerN+H]$^+$); 329 (87); 330 (100); 331 (17); 342 (33); 343 (14, [TerNP−Me]$^+$); 344 (40, [TerNP−Me+H]$^+$); 345 (11); 357 (20, [TerNP−H]$^+$); 358 (85, [TerNP]$^+$); 359 (35, [TerNP+H]$^+$); 360 (19); 375 (27); 519 (22); 534 (12); 655 (11); 670 (11); 671 (87); 672 (64); 673 (14); 686 (15); 891 (1, [M−H]$^+$); 892 (1, [M]$^+$).

Figure S7: NMR and IR spectra of **4^{i}Pr** (solvent signals indicated by asterisks).

$^{31}P\{^{1}H\}$ NMR spectrum

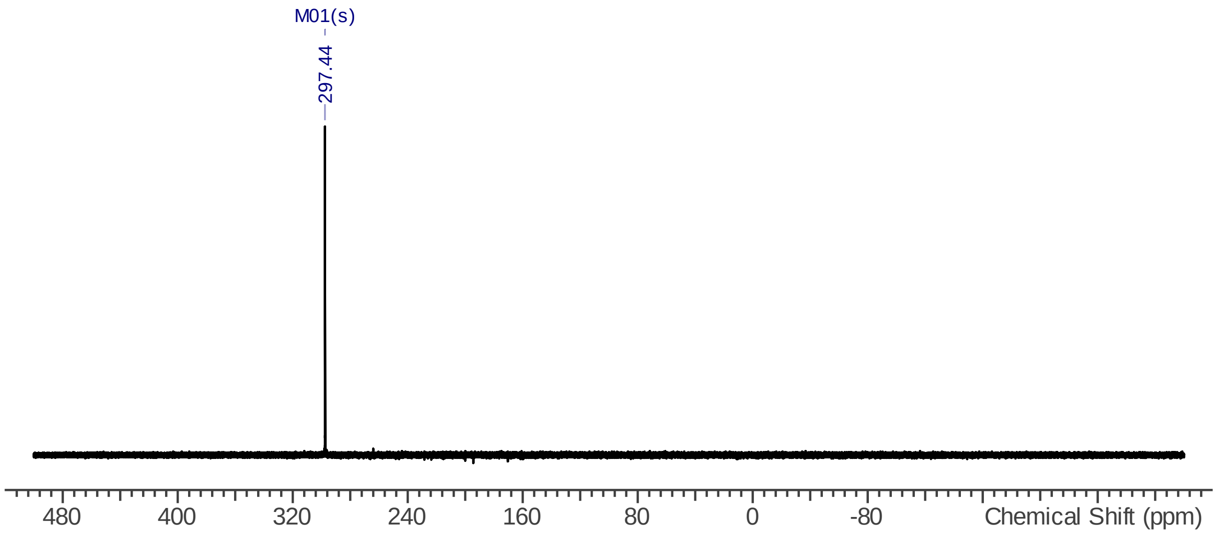

^{1}H NMR spectrum

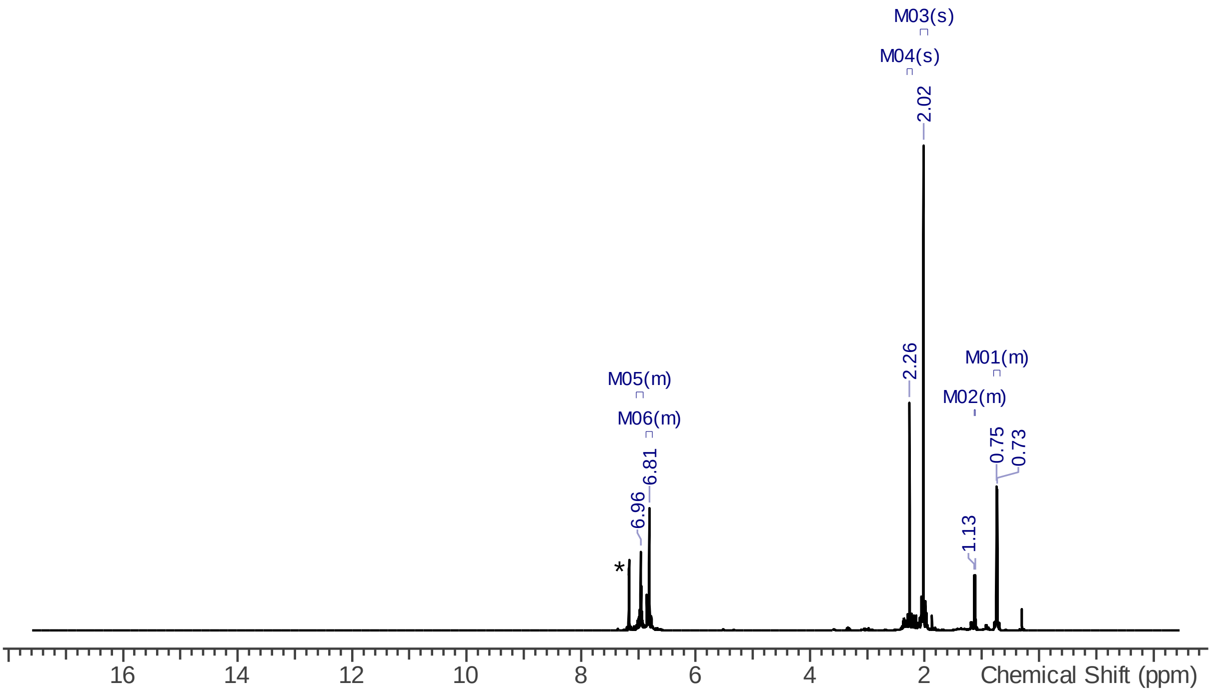

Figure S7 continued.

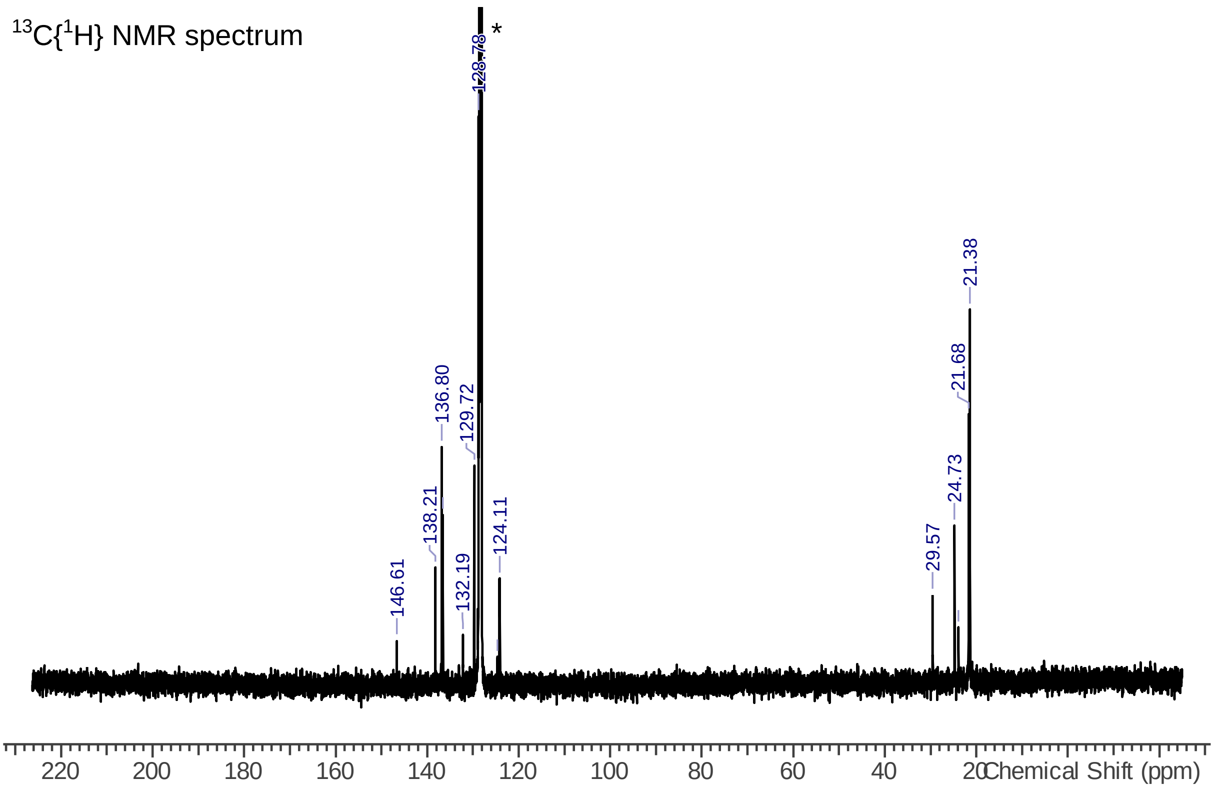

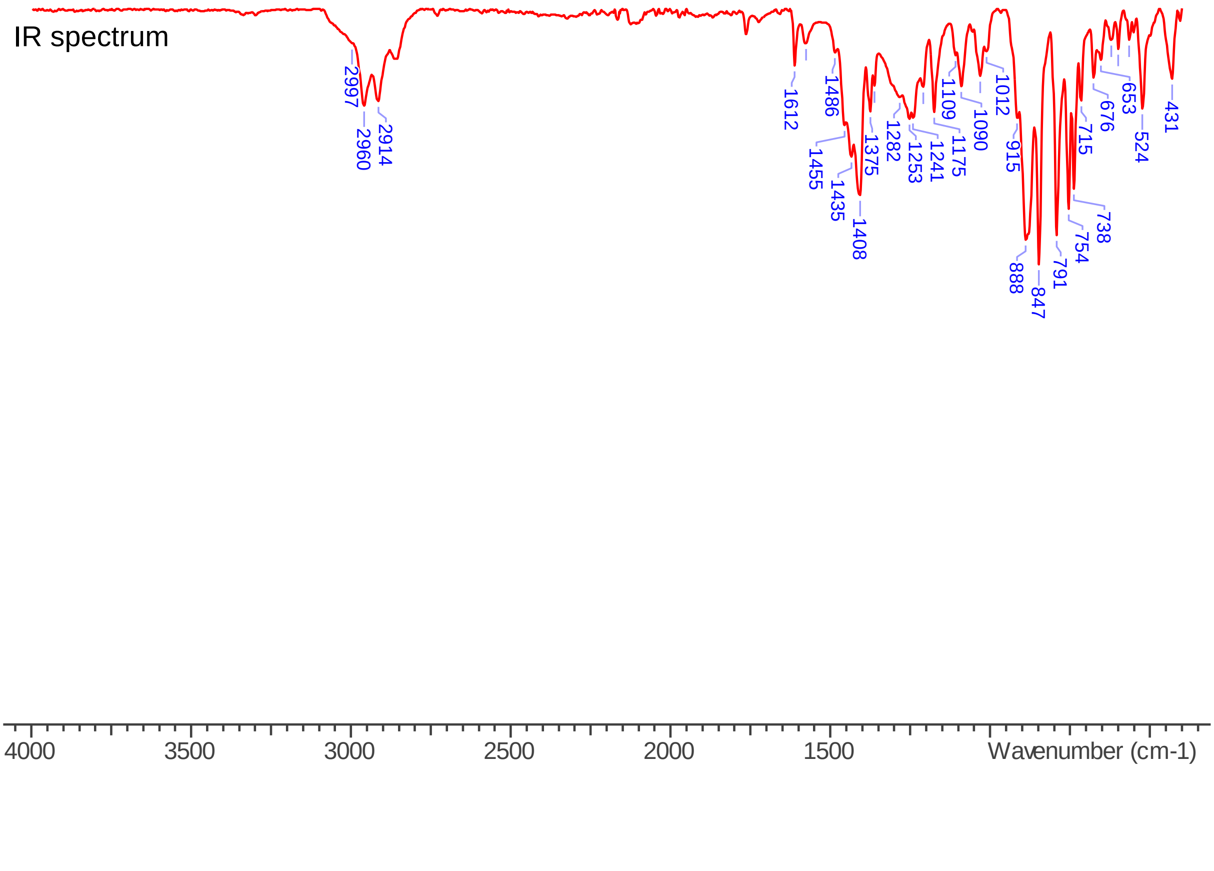

5 Additional spectroscopic details

5.1 Low temperature ^{31}P NMR spectroscopic measurements

As mentioned in section 4 the addition products **3Br** and **3*ⁱ*Pr** were obtained in the form of colorless crystals when reacting biradical **2** with the corresponding azides. However, if **3Br** and **3*ⁱ*Pr** are dissolved in fresh amounts of solvent (THF-*d*8) at room temperature the solution takes an intense blue color (Figure S8). ^{31}P NMR spectroscopy revealed, that in solution large amounts of starting material **2** are present again, causing the color of the solution to turn intensely blue (Figure S9 and Figure S10). If the addition products **3Br** and **3*ⁱ*Pr** are dissolved in cold THF-*d*8 (−40 °C) the color of the solution turns just slightly blue with only trace amounts of biradical **2** present (Figure S8).

Figure S8: A solution of **3*ⁱ*Pr** in THF-*d*8 at room temperature (left) and previously cooled THF-*d*8 (−40 °C, right).

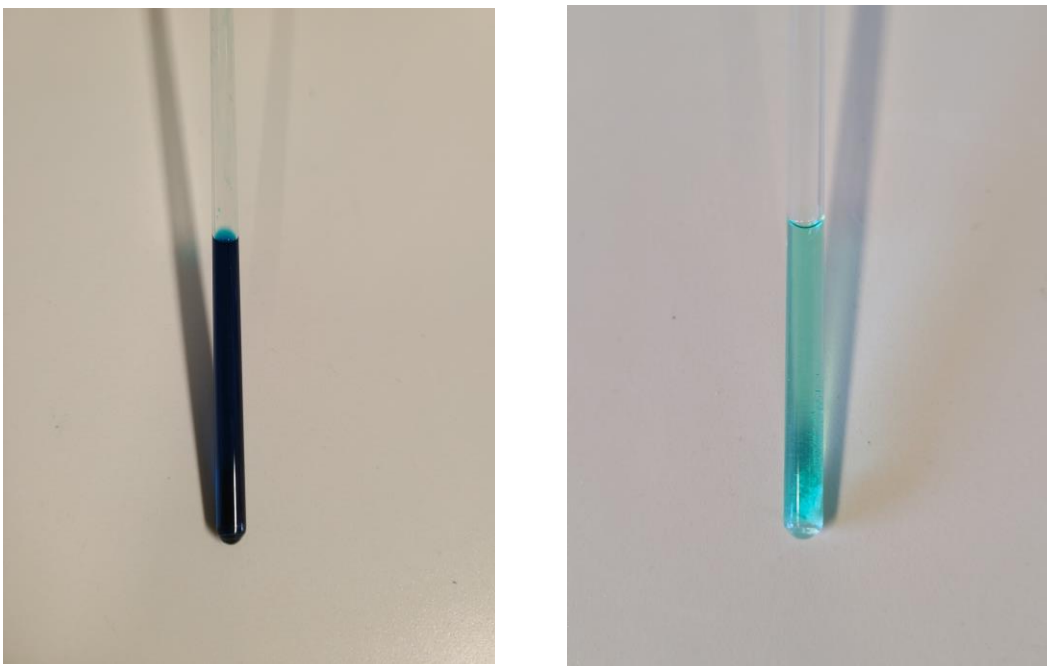

Therefore, compounds **3Br** and **3^{i}Pr** are not stable in solution at room temperature because the corresponding azide is eliminated from the addition product with re-formation of five-membered biradical **2**.

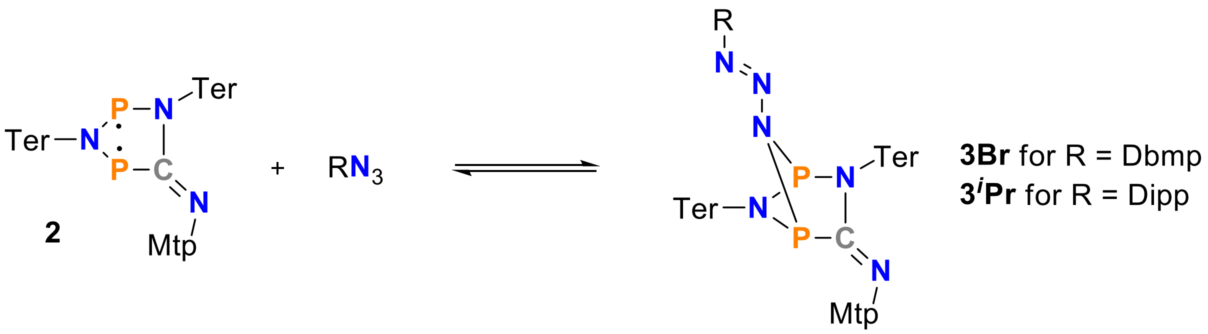

In order to get NMR data for pure compounds **3Br** and **3^{i}Pr**, low temperature NMR spectroscopic measurements were carried out. At −40 °C the equilibrium reaction with re-formation of biradical **2** is slowed down significantly, leaving **3Br** or **3^{i}Pr** as the main product in solution. This can also be seen in the corresponding ^{31}P NMR spectra at −40 °C (Figure S9 and Figure S10). For this the addition products **3Br** or **3^{i}Pr** were dissolved in cold THF-d_8 (−40 °C) inside an argon-filled glovebox and the sample transferred to the NMR spectrometer inside a previously cooled aluminium block (−40 °C). The sample was left in the NMR spectrometer for approximately 1 hour at −40 °C before the spectra were collected.

Figure S9: $^{31}P\{^1H\}$ NMR spectra of freshly dissolved **3Br** at room temperature (bottom) and at −40 °C (top) in THF-d_8.

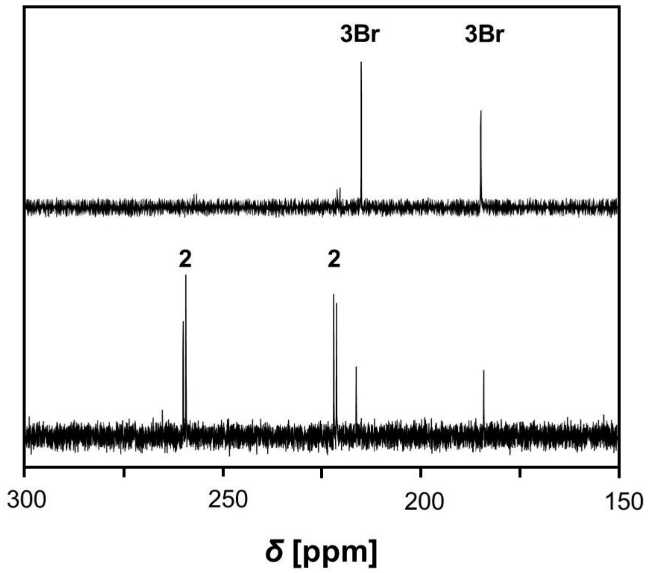

Figure S10: $^{31}P\{^1H\}$ NMR spectra of freshly dissolved **3^{i}Pr** at room temperature (bottom) and at −40 °C (top) in THF-d_8.

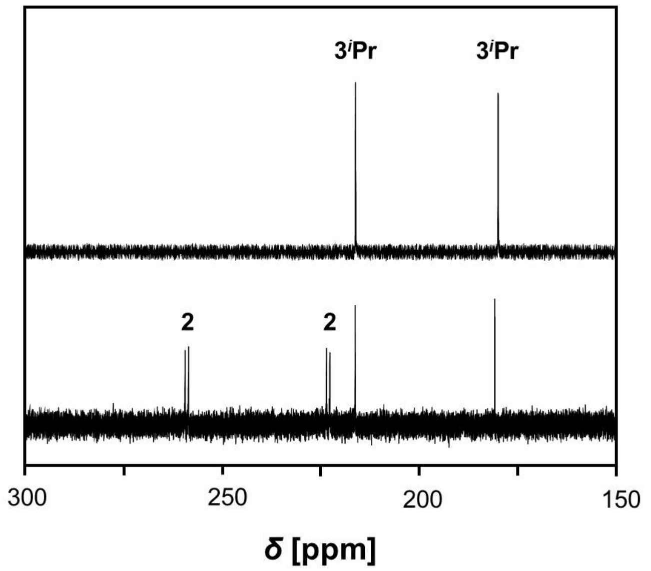

5.2 NMR and IR spectroscopic investigations

The reaction of the five-membered biradical **2** with different azides can be monitored by ^{31}P NMR spectroscopy. After stirring the solution of **2** with Dbmp-N_3 as described in section 4.1 for 2 h, the ^{31}P NMR spectrum shows that species **2**, **3Br** and **4Br** are present in the mixture at this stage (Figure S11, bottom). The addition product **3Br** is the main reaction product (singlets at 216.3 ppm and 183.9 ppm), with small amounts of starting material **2** and product **4Br** present alongside. This solution can be used to isolate **3Br** in the form of single crystals as described in section 4.1.

Figure S11: $^{31}P\{^1H\}$ NMR spectra of the reaction of **2** with Dbmp-N_3 after 2 h (bottom) and after 4 days (top) at room temperature, calibrated externally.

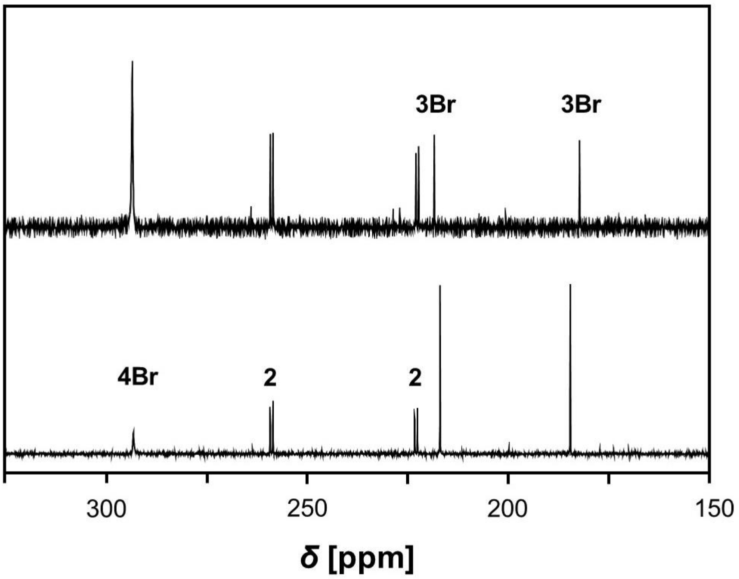

If the reaction is carried on and the mixture stirred for 4 days, a ^{31}P NMR spectrum of the solution shows, that only small amounts of addition product **3Br** are still present (Figure S11, top). The main product is the Staudinger type product **4Br** (heavily broadened singlet at 293.8 ppm) alongside significant amounts of starting material **2** (section 4.3, method A). This solution can no longer be used to isolate **3Br**, underlining the importance of isolating the addition products after 1-2 h of reaction time. Unfortunately, the time dependency leads to isolation of low-quality single crystals which show low resolution (**3Br**) or cannot be analysed accordingly by SCXRD (**3^{i}Pr**).

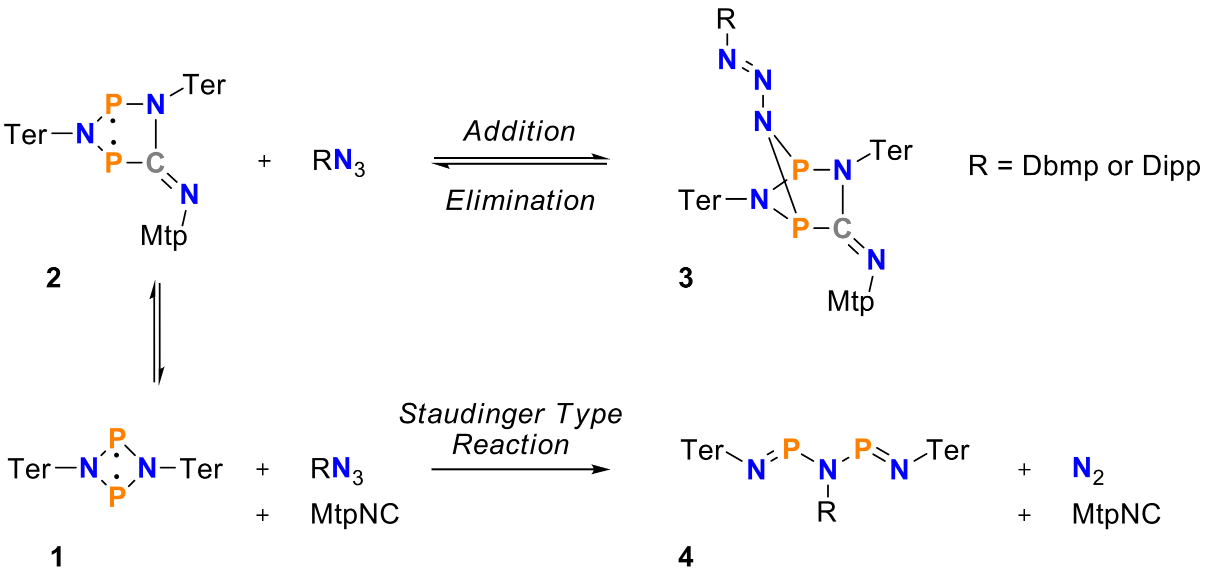

In addition to NMR spectroscopy the progress of the reaction can be investigated using IR spectroscopy. The IR spectrum of pure **3Br** for example can be seen in Figure S12 (blue line) and shows two characteristic bands located at 1632 cm^{-1} for the C=N stretching vibration (indicated by *) and at 1401 cm^{-1} for the N=N stretching vibration (indicated by **o**). Characteristic bands for a C≡N stretching vibration of MtpNC or N=N stretching vibrations of Dbmp-N_3 (2082 cm^{-1} and 2125 cm^{-1}) cannot be observed.[1] However, if **3Br** is dissolved in fresh benzene and the reaction to **4Br** commences, the IR spectrum changes significantly. If the solution is stirred for 4 days and the solvent removed *in vacuo* (1×10^{-3} mbar, 50 °C, water bath) the solid residue consists of small amounts of **3Br**, significant amounts of **2** and MtpNC as well as large amounts of **4Br** (see NMR spectra in Figure S11 as well). The corresponding IR spectrum (red line in Figure S12) of this mixture now shows a strong band for the C≡N stretching vibration of MtpNC at 2116 cm^{-1} (indicated by **+**). The signal for the C=N stretching vibration of **3Br** at 1632 cm^{-1} disappeared almost completely, while a signal for the C=N stretching vibration of **2** can be found at 1611 cm^{-1} (indicated by **#**). The signal for the N=N stretching vibration of **3Br** at 1401 cm^{-1} cannot be identified clearly anymore in the spectrum of the mixture.

Figure S12: Comparison of IR spectra of pure **3Br** (blue) and a mixture of **4Br**, **2**, MtpNC and trace amounts of **3Br** after 4 days of reaction time (red), ATR measurements, 32 scans.

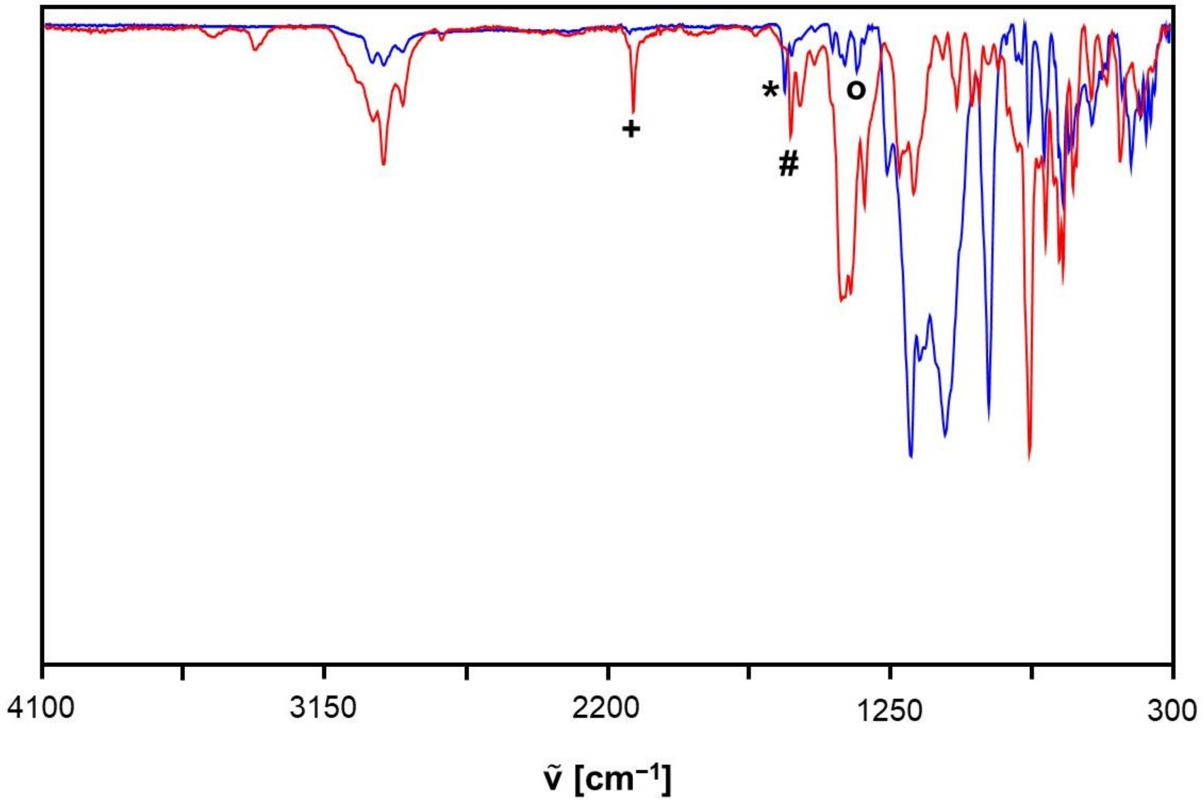

5.3 ^{31}P NMR data of 3Br, 3^{i}Pr, 4Br and 4^{i}Pr.

The following tables contain experimental ^{31}P NMR data. Calculated values for **3Br** and **4Br** are given in brackets for comparison (GIAO method, PBE0-D3/def2-SVP, see section 6 for further details).

Table S3. ^{31}P NMR data of isomers of **3Br** and **3^{i}Pr**.

	δ **(P1, N*P*N) [ppm]**	***δ*** **(P2, N*P*C) [ppm]**
**3Br**[a]	216.3 (172.2)	183.9 (158.3)
**3Br**[b]	214.8	184.5
**3^{i}Pr**[a]	216.4	180.9
**3^{i}Pr**[b]	216.1	179.9

[a] values at room temperature (approx. 25 °C), [b] values at −40 °C.

Table S4. ^{31}P NMR data of isomers of **4Br** and **4^{i}Pr**.

	δ **(P, N*P*N) [ppm]**
**4Br**[a]	293.8 (289.0)
**4^{i}Pr**[a]	297.4

[a] values at room temperature (approx. 25 °C)

6 Computational details

6.1 General remarks

Computations were carried out using Gaussian09[13] or ORCA 5.0.4[14] and the standalone version of NBO 6.0.[15–18]

Structure optimizations employed the DFT functional PBE[19–21] in conjunction with Grimme's dispersion correction D3(BJ)[22,23] and the def2-TZVP basis set[24] (notation PBE-D3/def2-TZVP). The resolution-of-identity (RI) approximation was applied for the pure functional PBE, using Weigend's accurate Coulomb-fitting basis set (W06).[25] All structures were fully optimized and confirmed as minima by frequency analyses.

Chemical shifts and coupling constants were derived by the GIAO method[26–30] at the PBE0-D3/def2-SVP level of theory.[19–21,31] The calculated absolute shifts for ^{31}P nuclei ($\sigma_{calc,X}$) were referenced to the experimental absolute shift of 85% H_3PO_4 in the gas phase ($\sigma_{ref,1}$ = 328.35 ppm),[32] using PH_3 ($\sigma_{ref,2}$ = 594.45 ppm) as a secondary standard:[33]

$$\begin{aligned}\delta_{calc,X} &= (\sigma_{ref,1} - \sigma_{ref,2}) - (\sigma_{calc,X} - \sigma_{calc,PH_3}) \\ &= \sigma_{calc,PH_3} - \sigma_{calc,X} - 266.1\ \text{ppm}\end{aligned}$$

At the PBE0-D3/def2-SVP level of theory, σ_{calc,PH_3} amounts to +629.17 ppm. Spin-spin coupling constants were calculated in a two-step process, using a modified basis set for computation of the Fermi contact term.[34]

More accurate electronic energies for optimized structures were computed by single-point DLPNO-CCSD(T)[35–38] calculations employing the def2-TZVP basis set and def2-TZVP/C correlation fitting basis (notation: DLPNO-CCSD(T)/def2-TZVP//PBE-D3/def2-TZVP).[24,31] Thermodynamic quantities at this level of theory were calculated using the DLPNO-CCSD(T) single point energy and the thermal corrections at the PBE-D3/def2-TZVP level of theory. The T_1 diagnostic was evaluated to ensure reliable results (empirically, CCSD(T) results are considered reliable if T_1 < 0.02).[39] Nonetheless, it should be noted that many of the species discussed here possess at least a small amount of multi-reference character (biradical character), so the use of single-reference methods always entails some loss of accuracy and should be regarded as an approximation!

Please note that all computations were carried out for single, isolated molecules in the gas phase (ideal gas approximation). There may well be significant differences between gas phase and condensed phase.

Figure S13: Gibbs free energy of the reaction of RN_3 with **2** at the DLPNO-CCSD(T)/def2-TZVP//PBE-D3/def2-TZVP level of theory ($c°$ = 1 mol/L).

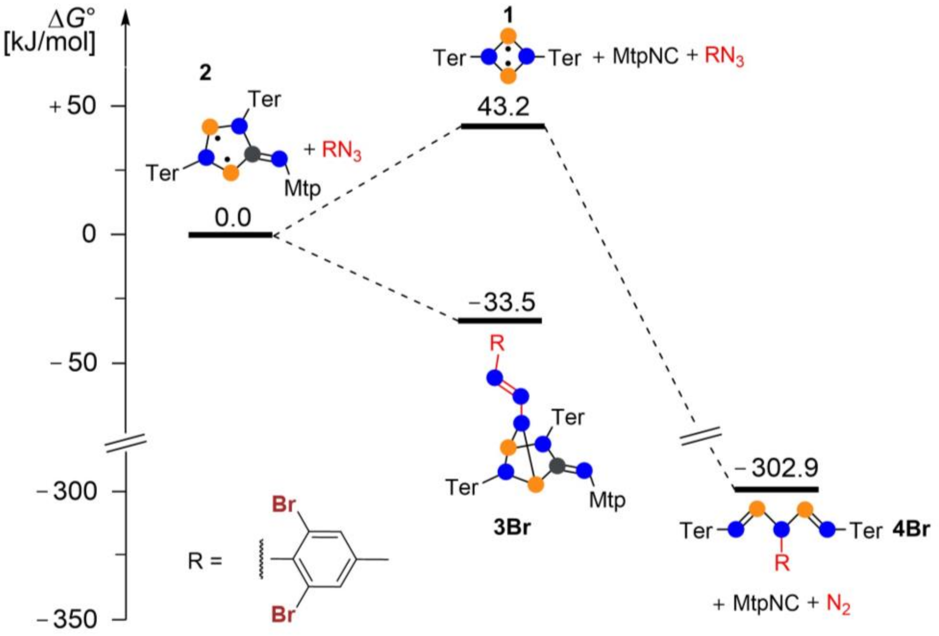

Figure S14: Schematic representation of the frontier MOs of model system **2H** (C_s symmetry) and HN_3 (C_s symmetry) with possible interactions shown.

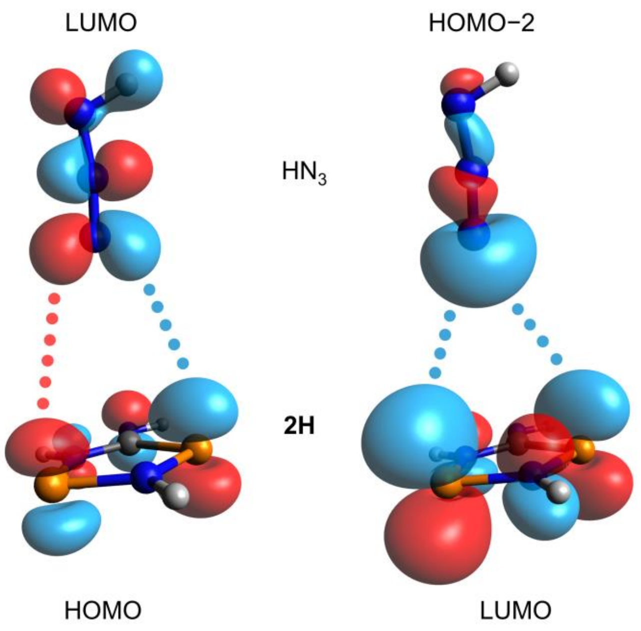

6.2 Reaction mechanism

The reaction mechanism of the azide addition reaction and isonitrile insertion was first investigated using a simple model system, where all Ter, Mtp and Dbmp substituents were replaced by Me groups. Several transition states were located on the PES using the Nudged Elastic Band (NEB) algorithm[40–44] implemented in ORCA[45] at the PBE-D3/def2-SVP level of theory. In total three possible reaction pathways were found: two for the MeNC insertion reaction (Figure S15 and Figure S17) and one for the MeN_3 addition reaction (Figure S19). All minima and TS structures of these reaction mechanisms were optimized at the PBE-D3/def2-TZVP level of theory and confirmed as minima or transition states by frequency analyses. Single point calculations at the DLPNO-CCSD(T)/def2-TZVP//PBE-D3/def2-TZVP level of theory were carried out as described above for comparison (Figure S16, Figure S18 and Figure S20).

A possible mechanism for the direct reaction from the addition product **3Me** to the corresponding staudinger type reaction product was not found. Different intermediate species and TS structures could not be located on the PES using the NEB algorithm at the PBE-D3/def2-SVP level of theory.

The mechanism for the azide addition was also investigated using the complete molecular structures of **2** and RN_3 with R = Dbmp using the NEB algorithm implemented in ORCA at the PBE-D3/def2-SVP level of theory. Due to the flatness of the potential and a vast number of degrees of freedom for the Me groups in the molecules, no electronic barriers could be found.

Figure S15: Gibbs free energy of the reaction of **1Me** to **2Me** (first pathway) at the PBE-D3/def2-TZVP level of theory ($c°$ = 1 mol/L).

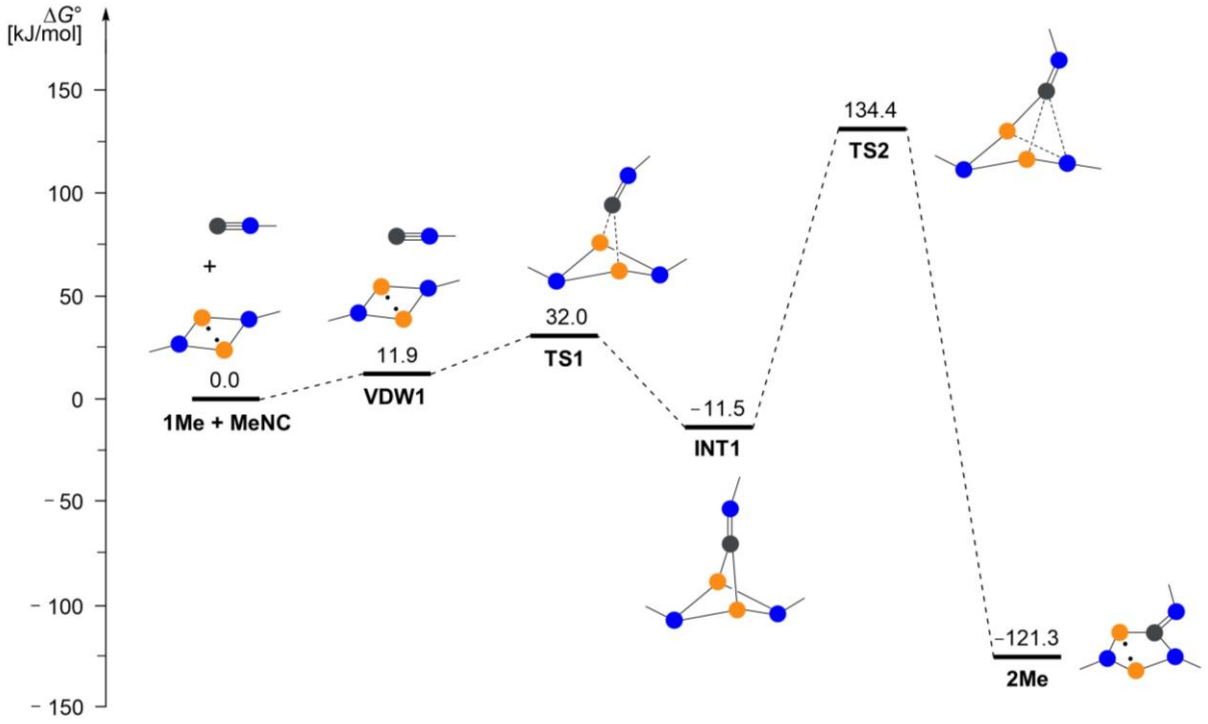

Figure S16: Gibbs free energy of the reaction of **1Me** to **2Me** (first pathway) at the DLPNO-CCSD(T)/def2-TZVP//PBE-D3/def2-TZVP level of theory ($c°$ = 1 mol/L).

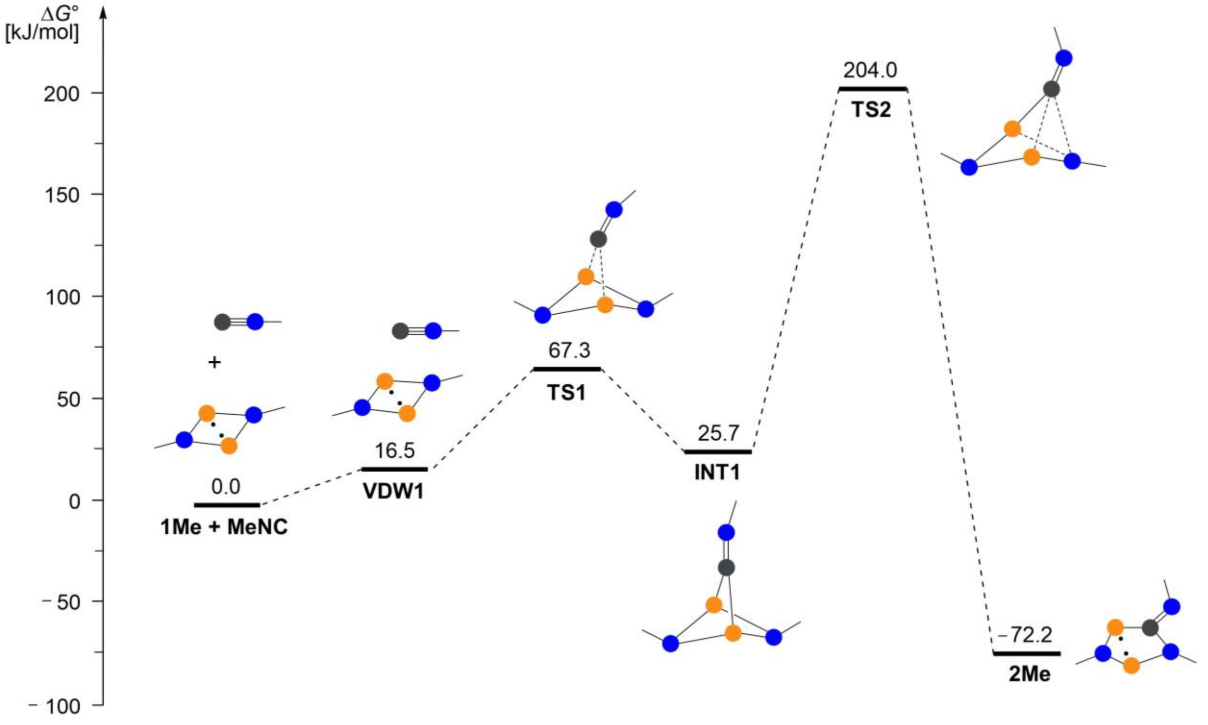

Figure S17: Gibbs free energy of the reaction of **1Me** to **2Me** (second pathway) at the PBE-D3/def2-TZVP level of theory ($c°$ = 1 mol/L).

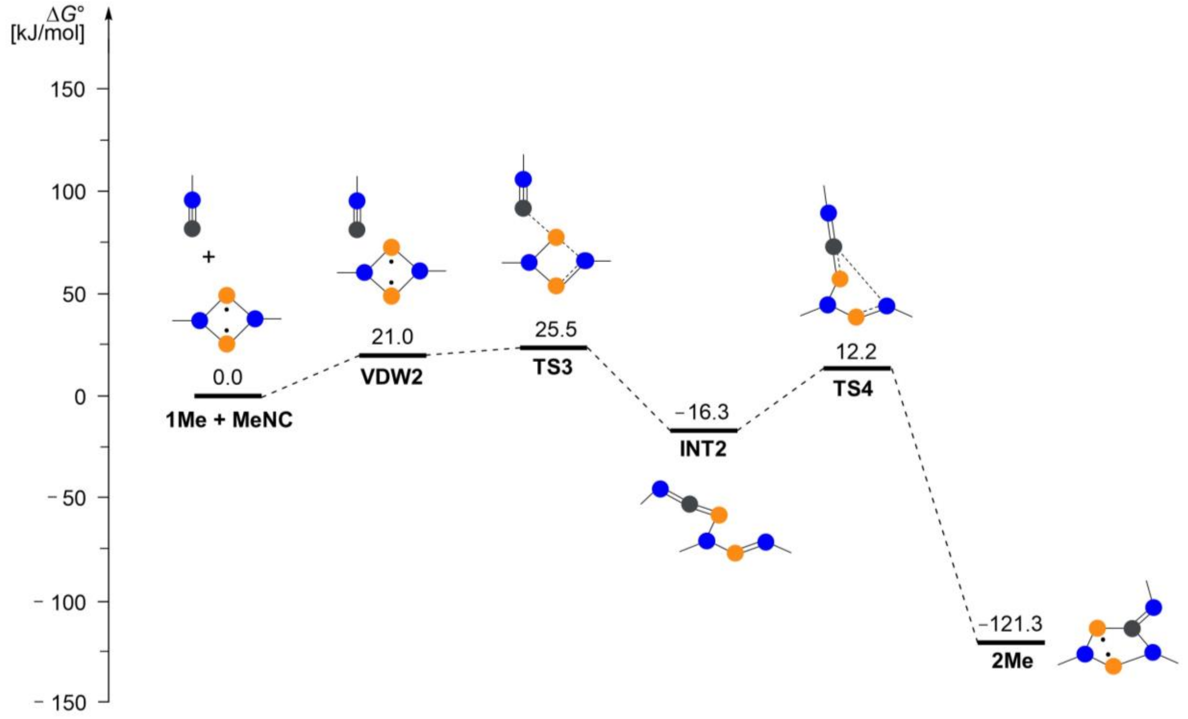

Figure S18: Gibbs free energy of the reaction of **1Me** to **2Me** (second pathway) at the DLPNO-CCSD(T)/def2-TZVP//PBE-D3/def2-TZVP level of theory ($c°$ = 1 mol/L).

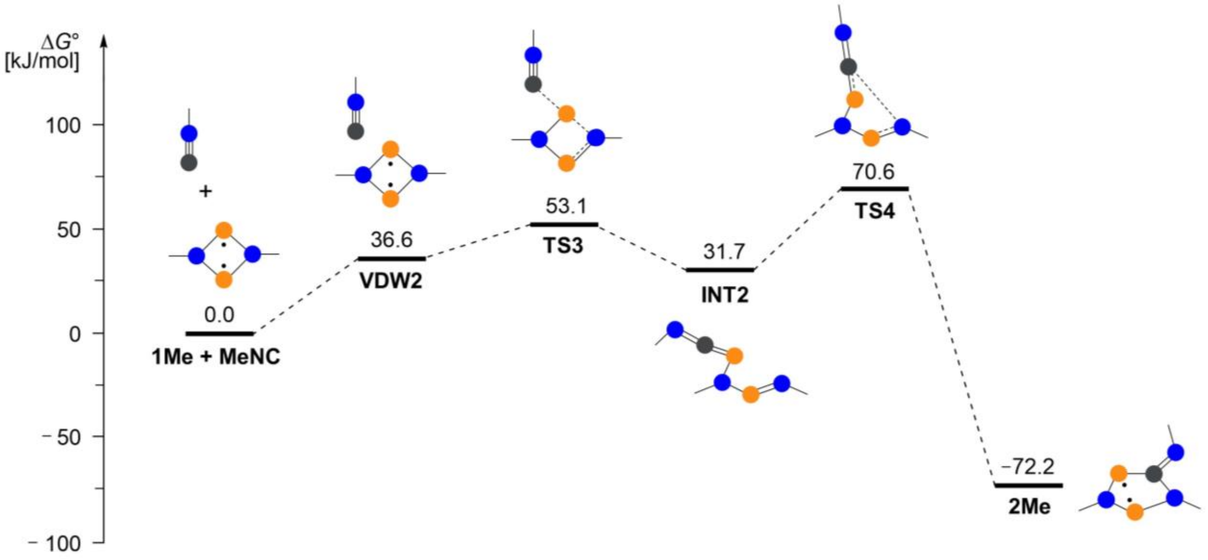

Figure S19: Gibbs free energy of the reaction of MeN_3 with **2Me** at the PBE-D3/def2-TZVP level of theory ($c°$ = 1 mol/L).

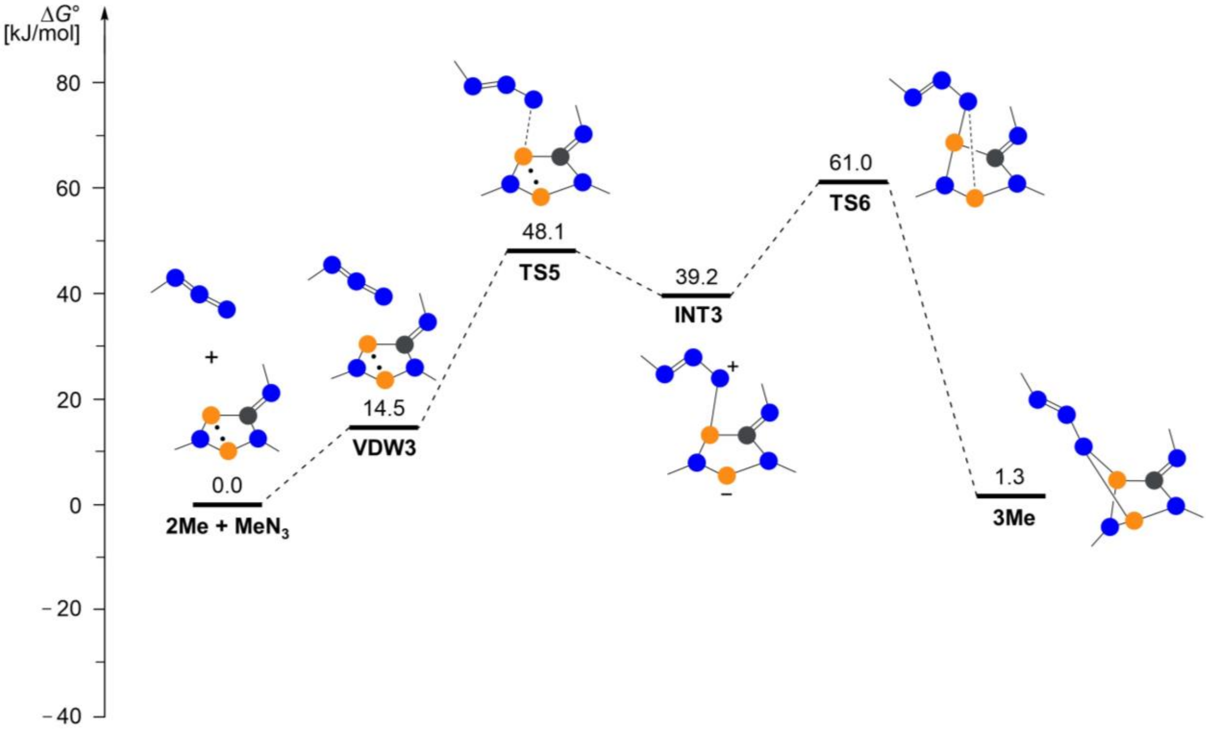

Figure S20: Gibbs free energy of the reaction of MeN_3 with **2Me** at the DLPNO-CCSD(T)/def2-TZVP//PBE-D3/def2-TZVP level of theory ($c°$ = 1 mol/L).

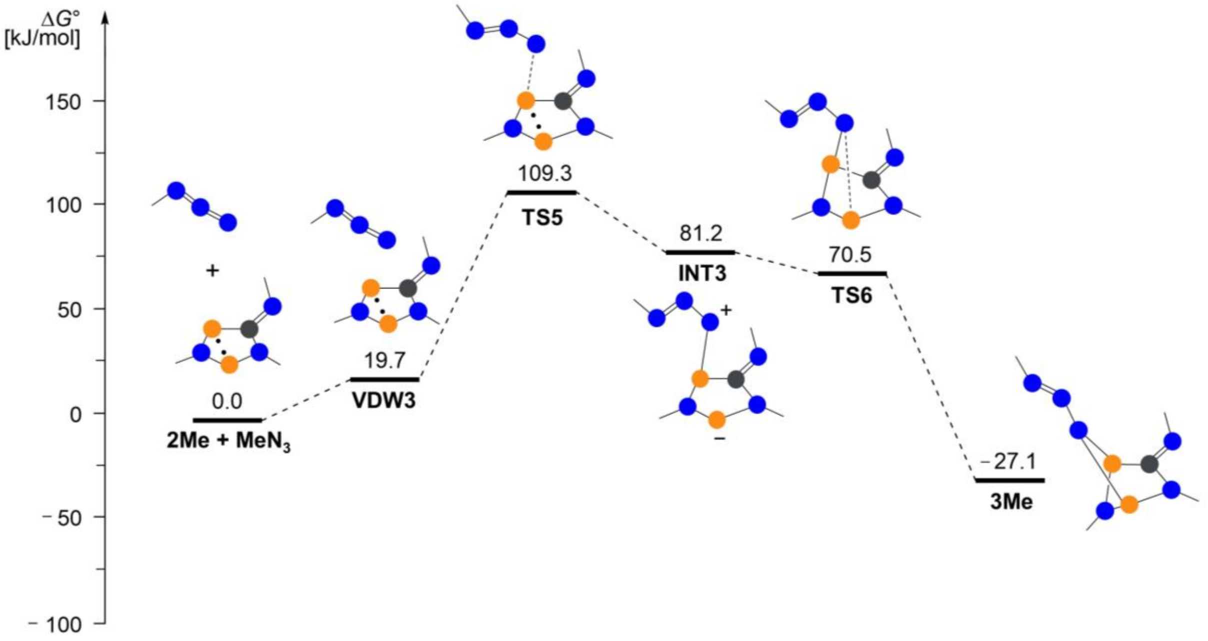

6.3 NBO Analysis and NLMOs of 3Br

NBO analysis revealed that the charge of the azide fragment in compound **3Br** sums up to −0.9. The remaining part of the addition product **3Br** including the phosphorus containing five-membered ring system has a charge of +0.9, as **3Br** has a charge of 0.0 in total.

Figure S21: Selected NLMOs of **3Br** at the PBE-D3/def2-TZVP level of theory. The orbital occupation number of the NBO calculation are given.

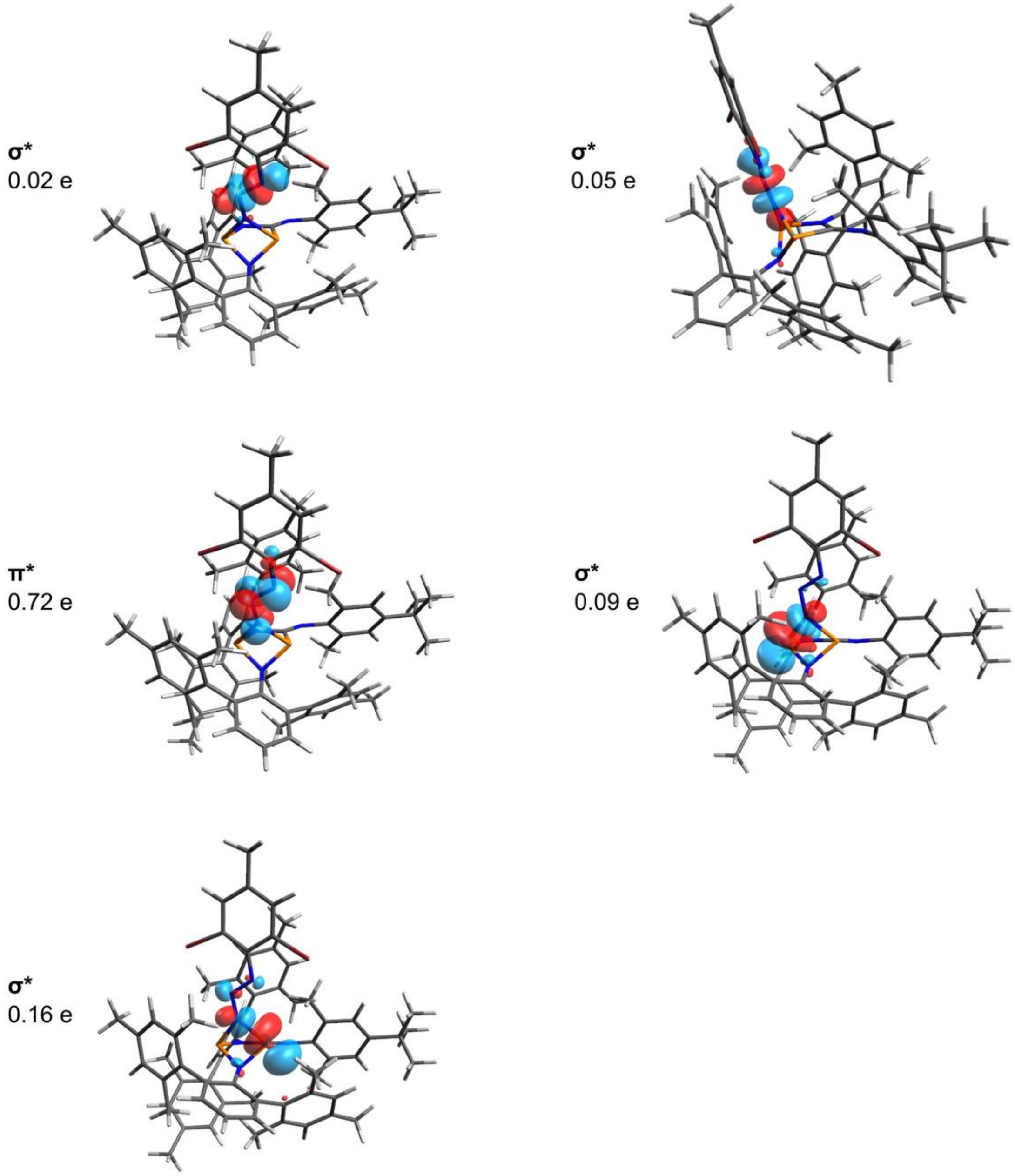

Figure S21 continued.

LP
1.90 e

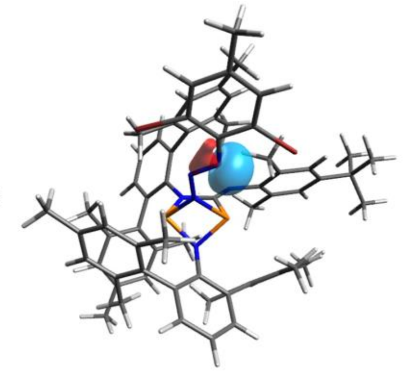

LP
1.24 e

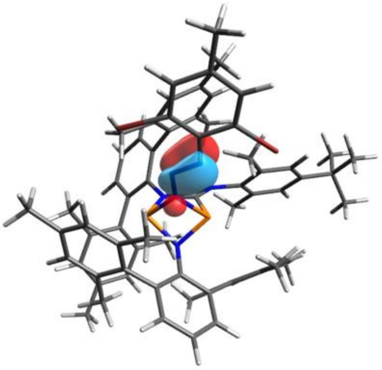

LP
1.89 e

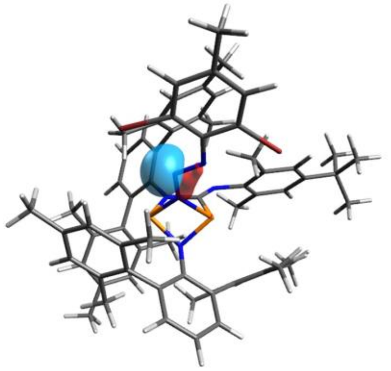

σ
1.98 e

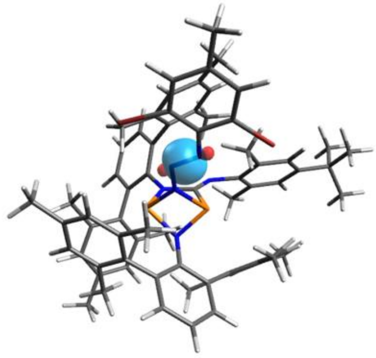

σ
1.99 e

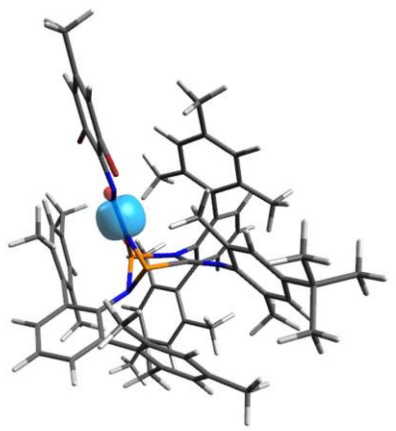

π
1.94 e

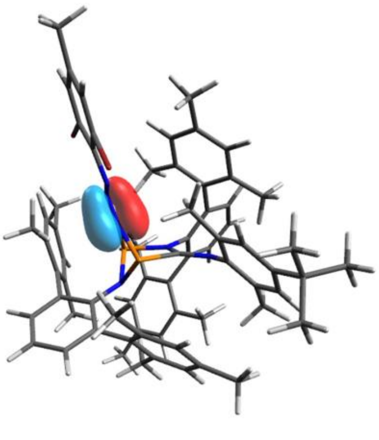

σ
1.96 e

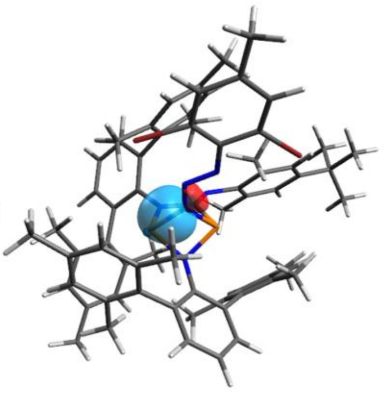

σ
1.96 e

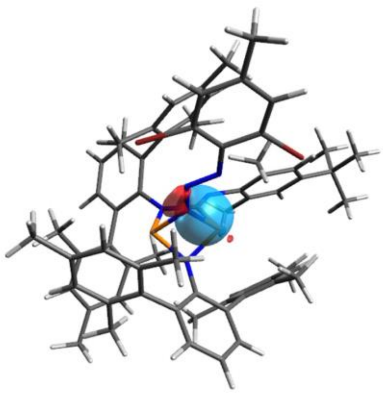

6.4 Summary of calculated data

Table S5. Summary of calculated data, including electronic energies, thermal corrections and chemical shifts.

Compd	PG	Opt. method	E_{tot}[a]	ΔG[b]	$E_{CCSD(T)}$[c]	T_1	δ (P1) [ppm]	δ (P2) [ppm]
**3Br**	C_1	PBE-D3 def2-TZVP	−8790.5922	1.0715	−8784.8153	0.01	172.2	158.3
**MtpNC**	C_1		−559.8314	0.2165	−559.2783	0.01	-	-
**1**	C_1		−2649.5089	0.7303	−2646.8693	0.01	-	-
**2**	C_1		−3209.3921	0.9735	−3206.1878	0.011	-	-
**Dbmp-N$_3$**	C_1		−5581.1636	0.0653	−5578.5851	0.01	-	-
**N$_2$**	$D_{\infty h}$		−109.4524	−0.013	−109.3765	0.013	-	-
**4Br**	C_1		−8121.3135	0.8104	−8116.2113	0.01	289.0	289.0
**1Me**	C_1		−871.5862	0.0544	−870.7905	0.011	-	-
**2Me**	C_1		−1004.2530	0.0991	−1003.3233	0.017	-	-
**3Me**	C_1		−1208.2292	0.1449	−1207.1157	0.014	-	-
**MeNC**	C_1		−132.5984	0.0196	−132.4832	0.014	-	-
**MeN$_3$**	C_1		−203.9563	0.0224	−203.7617	0.018	-	-
**VDW1**	C_1		−1004.1949	0.0918	−1003.2822	0.012	-	-
**VDW2**	C_1		−1004.1903	0.0906	−1003.2734	0.012	-	-
**VDW3**	C_1		−1208.2206	0.1413	−1207.0942	0.018	-	-
**INT1**	C_1		−1004.2097	0.0977	−1003.2846	0.015	-	-
**INT2**	C_1		−1004.2075	0.0936	−1003.2783	0.015	-	-
**INT3**	C_1		−1208.2127	0.1429	−1207.0724	0.018	-	-
**TS1**	C_1		−1004.1896	0.0960	−1003.2671	0.013	-	-
**TS2**	C_1		−1004.1512	0.0947	−1003.2137	0.015	-	-
**TS3**	C_1		−1004.1901	0.0922	−1003.2686	0.013	-	-
**TS4**	C_1		−1004.1958	0.0928	−1003.2626	0.015	-	-
**TS5**	C_1		−1208.2087	0.1440	−1207.0629	0.02	-	-
**TS6**	C_1		−1208.2055	0.1451	−1207.0787	0.017	-	-
**2H**	C_s		−886.4508	0.0244	-	-	-	-
**HN$_3$**	C_s		−164.6911	−0.0021	-	-	-	-

[a] Total SCF energy in a.u.; [b] thermal correction to Gibbs energy in a.u. (298 K unless stated otherwise); [c] single-point DLPNO-CCSD(T)/def2-TZVP energy.

6.5 Optimized structures (.xyz-files)

6.5.1 PH_3

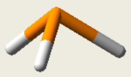

```
4
PH3 @ PBE-D3/def2-SVP
P          0.00000          0.00000          0.13012
H          0.00000          1.19618         -0.65060
H          1.03592         -0.59809         -0.65060
H         -1.03592         -0.59809         -0.65060
```

6.5.2 3Br

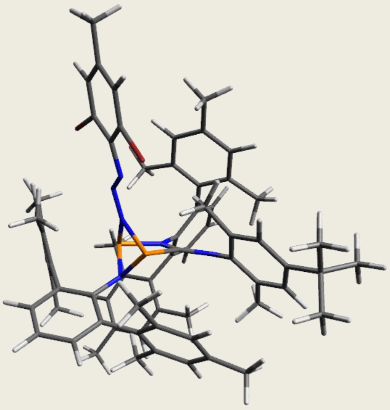

```
150
3Br @ PBE-D3/def2-TZVP
Br        -3.79686          2.60675         -2.12544
C         -2.91632          4.06294         -1.26405
C         -1.61030          3.88123         -0.72959
C         -3.60237          5.26845         -1.21966
N         -1.12591          2.57228         -0.77879
C         -1.02032          5.06595         -0.21532
H         -4.60483          5.32409         -1.64460
C         -3.01422          6.39976         -0.64368
N          0.04679          2.35520         -0.32031
Br         0.77936          5.15942          0.42145
C         -1.70850          6.27800         -0.16329
C         -3.76482          7.69880         -0.54271
N          0.43888          1.11365         -0.42640
H         -1.20039          7.14699          0.25655
H         -4.37371          7.87942         -1.43963
H         -3.07996          8.54720         -0.41314
H         -4.45004          7.69028          0.32005
P          1.94016          0.29560          0.11286
P         -0.28788         -0.46685         -1.00086
N          1.03288         -0.64365          1.30290
N          1.45631         -0.80526         -1.20057
C         -0.17360         -1.12406          0.77531
C          1.38206         -0.81818          2.68106
C          1.86659         -0.79455         -2.56779
N         -0.94576         -1.91606          1.40634
C          2.57287         -1.47841          3.03803
C          0.51209         -0.33262          3.69003
C          1.24329         -1.73776         -3.42726
C          2.81637          0.10744         -3.09710
C         -2.20265         -2.35864          0.99051
C          2.83578         -1.71864          4.39700
C          3.61806         -1.94608          2.08164
C          0.79964         -0.61925          5.02916
C         -0.62110          0.58942          3.40551
C          1.54961         -1.72404         -4.79226
C          0.32385         -2.79275         -2.90673
C          3.10701          0.07355         -4.46719
C          3.53263          1.06987         -2.21920
C         -2.49778         -3.72583          1.23719
C         -3.21850         -1.50932          0.49502
H          3.75794         -2.24167          4.65716
```

C	1.94628	-1.32299	5.38890
C	4.82400	-1.21728	1.98267
C	3.49685	-3.20417	1.45760
H	0.11979	-0.24314	5.79532
C	-1.95955	0.21782	3.65183
C	-0.31595	1.91242	3.01213
C	2.47272	-0.82388	-5.32136
H	1.06265	-2.45742	-5.43771
C	0.88364	-3.88902	-2.20961
C	-1.06091	-2.76750	-3.18160
H	3.84363	0.78005	-4.85505
C	4.71717	0.65086	-1.57363
C	3.05817	2.38801	-2.05345
C	-3.76670	-4.21556	0.94685
C	-1.43673	-4.62605	1.79983
C	-4.48199	-2.05761	0.22528
C	-3.02955	-0.03540	0.28461
H	2.15796	-1.53666	6.43766
C	5.89725	-1.77870	1.28435
C	4.96605	0.13693	2.62512
C	4.59495	-3.72255	0.76277
C	2.22035	-3.98972	1.56654
C	-2.97378	1.15065	3.40494
C	-2.31938	-1.14367	4.18666
C	-1.36298	2.80790	2.77377
C	1.10911	2.39287	2.93012
H	2.70394	-0.83125	-6.38719
C	0.04864	-4.92482	-1.78794
C	2.36354	-3.96157	-1.96233
C	-1.86279	-3.82068	-2.72164
C	-1.68890	-1.65094	-3.97253
C	5.35068	1.52667	-0.69011
C	5.28019	-0.71714	-1.84072
C	3.71419	3.22561	-1.14340
C	1.90097	2.90703	-2.86329
H	-3.96220	-5.27218	1.14565
C	-4.78915	-3.40221	0.43164
H	-1.86282	-5.58930	2.11103
H	-0.93912	-4.15530	2.66055
H	-0.64722	-4.82025	1.05696
H	-5.24789	-1.37505	-0.14566
H	-3.95691	0.50929	0.50975
H	-2.78012	0.20946	-0.75987
H	-2.24511	0.38788	0.92353
H	6.83512	-1.21955	1.22303
C	5.80739	-3.03242	0.67237
H	5.96663	0.55121	2.44597
H	4.79717	0.09553	3.71085
H	4.22689	0.84258	2.21445
H	4.50429	-4.70593	0.29397
H	2.33684	-4.99444	1.14057
H	1.39742	-3.48849	1.03631
H	1.89832	-4.08906	2.61344
H	-4.01269	0.84733	3.56276
C	-2.70141	2.43831	2.93405
H	-3.31502	-1.44806	3.83659
H	-2.34191	-1.13973	5.28864
H	-1.59565	-1.90261	3.86780
H	-1.12203	3.82657	2.46136
H	1.61769	2.06077	2.00929

```
H          1.70542         2.01919         3.77444
H          1.14473         3.48852         2.92580
H          0.49126        -5.77699        -1.26392
C         -1.32945        -4.91311        -2.03569
H          2.63865        -4.92815        -1.52166
H          2.69187        -3.16893        -1.27442
H          2.93189        -3.83026        -2.89523
H         -2.93916        -3.78237        -2.90855
H         -2.76505        -1.58615        -3.76294
H         -1.56797        -1.81463        -5.05521
H         -1.23418        -0.67802        -3.74227
H          6.25989         1.19701        -0.18100
C          4.84200         2.80499        -0.43172
H          6.27860        -0.81976        -1.39975
H          5.34608        -0.91513        -2.92018
H          4.64566        -1.50680        -1.40955
H          3.32848         4.23607        -0.98988
H          1.46504         3.79960        -2.39793
H          1.11071         2.15547        -2.98605
H          2.23712         3.18040        -3.87652
C         -6.17720        -3.99168         0.15872
C          6.96539        -3.60421        -0.09903
C         -3.81058         3.38802         2.57171
C         -2.19355        -6.06920        -1.61405
C          5.48651         3.69791         0.59359
C         -6.76453        -4.53886         1.47593
C         -6.05806        -5.14367        -0.85895
C         -7.15199        -2.95089        -0.40841
H          6.99349        -4.70052        -0.02887
H          7.92462        -3.20975         0.26354
H          6.88807        -3.34863        -1.16879
H         -3.48639         4.43485         2.65304
H         -4.69360         3.24384         3.21003
H         -4.12841         3.22911         1.52790
H         -3.25764        -5.80862        -1.66032
H         -1.96406        -6.38794        -0.58724
H         -2.02799        -6.93911        -2.26990
H          5.27112         4.75705         0.39769
H          6.57716         3.56376         0.61691
H          5.10877         3.46603         1.60286
H         -6.86682        -3.73476         2.21897
H         -6.12334        -5.31839         1.91005
H         -7.75958        -4.97603         1.30018
H         -5.64533        -4.78077        -1.81199
H         -7.04762        -5.58250        -1.05901
H         -5.40347        -5.94464        -0.48834
H         -6.79234        -2.53322        -1.36020
H         -7.31336        -2.11942         0.29292
H         -8.12727        -3.42309        -0.59650
```

6.5.3 MtpNC

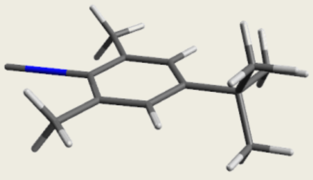

```
31
MtpNC @ PBE-D3/def2-TZVP
C         -4.39004        -0.02850        -0.00010
N         -3.20774        -0.01471        -0.00003
C         -1.82566         0.00143         0.00003
C         -1.15736         1.23875         0.00003
```

```
C          -1.12868        -1.22448         0.00007
C           0.24129         1.22395        -0.00000
C          -1.93067         2.52484         0.00003
C           0.26413        -1.17501         0.00004
C          -1.87528        -2.52619         0.00001
H           0.75568         2.18481        -0.00002
C           0.97778         0.03404         0.00000
H          -1.25318         3.38755        -0.00007
H          -2.58626         2.59612         0.88119
H          -2.58641         2.59603        -0.88103
H           0.80548        -2.12328         0.00010
H          -1.18013        -3.37475         0.00116
H          -2.52827        -2.61116        -0.88182
H          -2.53016        -2.61023         0.88051
C           2.51002         0.01036        -0.00003
C           3.00592        -0.73055         1.25884
C           3.11627         1.42018        -0.00033
C           3.00594        -0.73109        -1.25855
H           2.63807        -1.76517         1.29280
H           2.66626        -0.22096         2.17203
H           4.10577        -0.76242         1.27075
H           2.82096         1.99158        -0.89239
H           4.21322         1.34715        -0.00034
H           2.82103         1.99198         0.89150
H           2.66628        -0.22193        -2.17198
H           2.63818        -1.76575        -1.29207
H           4.10579        -0.76291        -1.27046
```

6.5.4 Biradical 1

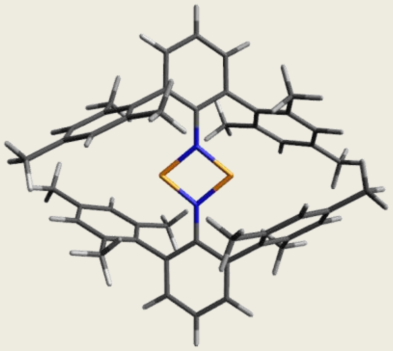

```
102
Biradical 1 @ PBE-D3/def2-TZVP
N           0.00003         1.11372        -0.00007
N          -0.00003        -1.11372        -0.00007
P           1.34194        -0.00004        -0.00006
P          -1.34194         0.00004        -0.00006
C          -0.00003        -2.51578        -0.00012
C           1.15143        -3.22973         0.41354
C          -1.15150        -3.22968        -0.41387
C           1.13061        -4.62875         0.40407
C          -1.13064        -4.62869        -0.40470
C           0.00000        -5.33557        -0.00040
H           2.02578        -5.15968         0.73418
H          -2.02581        -5.15959        -0.73487
H           0.00002        -6.42629        -0.00051
C           0.00003         2.51578        -0.00012
C           1.15150         3.22968        -0.41387
C          -1.15143         3.22973         0.41354
C           1.13064         4.62869        -0.40470
C          -1.13061         4.62875         0.40407
C           0.00000         5.33557        -0.00040
H           2.02581         5.15959        -0.73487
H          -2.02578         5.15968         0.73418
H          -0.00002         6.42629        -0.00051
C          -2.37992         2.53304         0.88859
C          -3.49601         2.39693         0.03717
C          -2.42981         2.03339         2.21090
C          -4.64170         1.75734         0.52329
```

C	-3.59452	1.40473	2.65385
C	-4.71023	1.25039	1.82254
H	-5.50011	1.63747	-0.14235
H	-3.62691	1.01253	3.67423
C	2.38006	2.53292	-0.88865
C	3.49601	2.39690	-0.03703
C	2.43017	2.03319	-2.21092
C	4.64180	1.75732	-0.52294
C	3.59497	1.40453	-2.65364
C	4.71056	1.25030	-1.82215
H	5.50011	1.63752	0.14283
H	3.62752	1.01224	-3.67398
C	-2.38006	-2.53292	-0.88865
C	-3.49601	-2.39690	-0.03703
C	-2.43017	-2.03319	-2.21092
C	-4.64180	-1.75732	-0.52294
C	-3.59497	-1.40453	-2.65364
C	-4.71056	-1.25030	-1.82215
H	-5.50011	-1.63752	0.14283
H	-3.62752	-1.01224	-3.67398
C	2.37992	-2.53304	0.88859
C	2.42981	-2.03339	2.21090
C	3.49601	-2.39693	0.03717
C	3.59452	-1.40473	2.65385
C	4.64170	-1.75734	0.52329
C	4.71023	-1.25039	1.82254
H	3.62691	-1.01253	3.67423
H	5.50011	-1.63747	-0.14235
C	-3.44379	-2.86425	1.39292
H	-4.42357	-2.75195	1.87543
H	-3.13335	-3.91528	1.47445
H	-2.71311	-2.26895	1.96422
C	-1.23166	-2.13903	-3.11353
H	-0.84635	-3.16816	-3.15772
H	-1.47728	-1.81192	-4.13212
H	-0.40848	-1.50682	-2.74194
C	-5.94608	-0.54969	-2.31948
H	-5.71526	0.47646	-2.64496
H	-6.38102	-1.07118	-3.18596
H	-6.71585	-0.49323	-1.53818
C	3.44402	-2.86423	-1.39281
H	4.42372	-2.75128	-1.87535
H	3.13426	-3.91545	-1.47439
H	2.71294	-2.26938	-1.96405
C	1.23115	-2.13925	3.11331
H	0.40846	-1.50610	2.74221
H	0.84510	-3.16813	3.15652
H	1.47688	-1.81322	4.13222
C	5.94562	-0.54971	2.32010
H	5.71478	0.47662	2.64500
H	6.38010	-1.07085	3.18701
H	6.71575	-0.49370	1.53912
C	5.94608	0.54969	-2.31948
H	5.71526	-0.47646	-2.64496
H	6.38102	1.07118	-3.18596
H	6.71585	0.49323	-1.53818
C	-5.94562	0.54971	2.32010
H	-5.71478	-0.47662	2.64500
H	-6.38010	1.07085	3.18701
H	-6.71575	0.49370	1.53912

```
C          -1.23115        2.13925        3.11331
H          -0.84510        3.16813        3.15652
H          -1.47688        1.81322        4.13222
H          -0.40846        1.50610        2.74221
C           1.23166        2.13903       -3.11353
H           0.84635        3.16816       -3.15772
H           1.47728        1.81192       -4.13212
H           0.40848        1.50682       -2.74194
C           3.44379        2.86425        1.39292
H           4.42357        2.75195        1.87543
H           3.13335        3.91528        1.47445
H           2.71311        2.26895        1.96422
C          -3.44402        2.86423       -1.39281
H          -4.42372        2.75128       -1.87535
H          -3.13426        3.91545       -1.47439
H          -2.71294        2.26938       -1.96405
```

6.5.5 Biradical 2

```
133
Biradical 2 @ PBE-D3/def2-TZVP
P           1.94113        0.42902        0.28027
P          -0.37780       -0.53709       -1.30754
N           0.44764        0.37702        1.08868
N           1.30566       -0.13114       -1.16601
C          -0.71524       -0.09649        0.40233
C           0.37973        0.71852        2.48746
C           2.14602       -0.25621       -2.33363
N          -1.82090       -0.23940        1.06048
C           1.20005        0.02930        3.40627
C          -0.49971        1.73491        2.93041
C           2.33264       -1.53342       -2.91005
C           2.73493        0.89158       -2.90486
C          -3.02719       -0.56302        0.43925
C           1.09099        0.33541        4.77198
C           2.20194       -1.01856        3.04458
C          -0.58170        1.99250        4.30506
C          -1.28144        2.62300        2.02599
C           3.10407       -1.63770       -4.07350
C           1.77816       -2.77104       -2.29134
C           3.51115        0.73300       -4.06371
C           2.57095        2.28053       -2.37959
C          -3.77876       -1.63205        1.00240
C          -3.59685        0.18024       -0.62639
H           1.72291       -0.21000        5.47507
C           0.19549        1.29642        5.22628
C           3.57800       -0.69275        3.05895
C           1.79449       -2.35623        2.86485
H          -1.25729        2.78062        4.64005
C          -2.69479        2.64230        2.09210
C          -0.60337        3.55050        1.20655
C           3.69246       -0.51512       -4.65115
H           3.25000       -2.62686       -4.51071
C           2.45209       -3.35423       -1.20101
C           0.61522       -3.37354       -2.81557
H           3.96110        1.62276       -4.50761
C           3.61493        2.87560       -1.64173
C           1.44059        3.04215       -2.74933
```

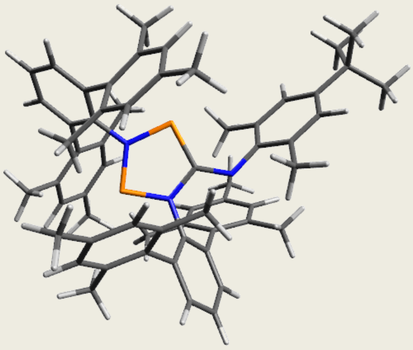

C	-5.02384	-1.95664	0.47468
C	-3.21295	-2.41533	2.15285
C	-4.85869	-0.19203	-1.11801
C	-2.93076	1.38376	-1.23534
H	0.11594	1.51584	6.29208
C	4.51871	-1.71433	2.90013
C	4.03778	0.72910	3.24608
C	2.77248	-3.34752	2.72934
C	0.33745	-2.72077	2.85511
C	-3.39520	3.55896	1.30394
C	-3.46357	1.70425	2.98487
C	-1.35057	4.44442	0.42783
C	0.89689	3.64606	1.18399
H	4.29307	-0.61324	-5.55672
C	1.91070	-4.50082	-0.60943
C	3.73910	-2.77406	-0.67897
C	0.10163	-4.50864	-2.18206
C	-0.07174	-2.81603	-4.03353
C	3.50707	4.22429	-1.28023
C	4.84135	2.09121	-1.25532
C	1.37843	4.38562	-2.36922
C	0.33323	2.43946	-3.56995
H	-5.56614	-2.79093	0.92771
C	-5.59560	-1.25607	-0.60023
H	-3.97942	-3.06306	2.59978
H	-2.80893	-1.74608	2.92666
H	-2.37465	-3.05203	1.82960
H	-5.26738	0.40512	-1.93516
H	-3.68539	2.13394	-1.51302
H	-2.37606	1.11852	-2.15215
H	-2.21632	1.85627	-0.55254
H	5.58143	-1.45623	2.89969
C	4.13739	-3.05236	2.75271
H	5.12572	0.80600	3.12121
H	3.77846	1.11156	4.24461
H	3.56118	1.40701	2.52061
H	2.45089	-4.38489	2.60666
H	0.20184	-3.78432	2.61902
H	-0.22756	-2.12927	2.12199
H	-0.12968	-2.52187	3.83251
H	-4.48809	3.55240	1.34166
C	-2.74545	4.46125	0.45483
H	-4.45593	1.50099	2.56060
H	-3.61279	2.13604	3.98763
H	-2.93633	0.75009	3.09906
H	-0.81693	5.15724	-0.20716
H	1.33290	3.03818	0.37332
H	1.34499	3.30709	2.12716
H	1.21700	4.68014	1.00442
H	2.43696	-4.95271	0.23482
C	0.72260	-5.07723	-1.06485
H	4.30624	-3.52882	-0.12054
H	3.55167	-1.93733	0.01322
H	4.37111	-2.39176	-1.49247
H	-0.81626	-4.95748	-2.57105
H	-1.03170	-3.32034	-4.20314
H	0.54498	-2.94405	-4.93664
H	-0.26722	-1.73865	-3.92971
H	4.31649	4.68092	-0.70424
C	2.40174	4.99914	-1.63860

```
H           5.49802         2.68844         -0.60954
H           5.41947         1.78142         -2.13902
H           4.57943         1.16851         -0.71479
H           0.50346         4.97187         -2.66329
H          -0.17039         1.62561         -3.02438
H           0.71863         2.00522         -4.50504
H          -0.42165         3.19493         -3.82265
C          -6.97143        -1.66451         -1.13703
C           5.17096        -4.13934          2.62863
C          -3.52955         5.39386         -0.42806
C           0.11646        -6.26157         -0.36123
C           2.29583         6.44437         -1.23347
C          -8.01806        -1.55220         -0.00984
C          -6.91255        -3.12453         -1.63123
C          -7.42732        -0.78057         -2.30571
H           4.73273        -5.06740          2.23669
H           5.61906        -4.37329          3.60752
H           5.99243        -3.83852          1.96202
H          -2.93357         6.27055         -0.71730
H          -4.44449         5.74625          0.06885
H          -3.83933         4.88537         -1.35600
H          -0.63280        -5.93374          0.37789
H           0.87784        -6.84239          0.17765
H          -0.39563        -6.93200         -1.06552
H           2.00803         7.07893         -2.08448
H           3.24626         6.81949         -0.83137
H           1.52716         6.58384         -0.45577
H          -8.08526        -0.51714          0.35567
H          -7.76113        -2.19391          0.84424
H          -9.01164        -1.85521         -0.37540
H          -6.17715        -3.23029         -2.44208
H          -7.89584        -3.44218         -2.01186
H          -6.62332        -3.81136         -0.82380
H          -6.72906        -0.83711         -3.15354
H          -7.51977         0.27356         -2.00576
H          -8.41339        -1.11563         -2.65987
```

6.5.6 Dbmp-N_3

```
17
Dbmp-N3 @ PBE-D3/def2-TZVP
Br          2.85055        -0.90497         -0.10677
C           1.34839         0.25508         -0.02503
C           0.05019        -0.29848         -0.05799
C           1.56324         1.62482          0.07389
N          -0.04769        -1.68932         -0.21193
C          -1.02292         0.61472         -0.01892
H           2.58665         1.99969          0.09961
C           0.48364         2.51347          0.14856
N          -0.92914        -2.36667          0.32421
Br         -2.81904         0.00679         -0.18865
C          -0.81069         1.98717          0.09614
C           0.71272         3.99309          0.29564
N          -1.63591        -3.15859          0.74722
H          -1.67384         2.65266          0.12551
H           1.58129         4.32291         -0.29074
H           0.90835         4.25625          1.34720
H          -0.16357         4.56753         -0.03199
```

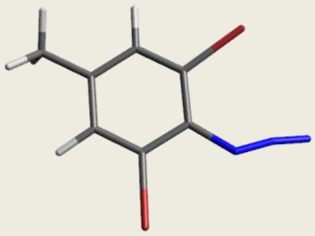

6.5.7 N_2

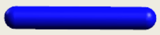

```
2
N₂ @ PBE-D3/def2-TZVP
N          -0.00000         0.00000         0.55145
N           0.00000         0.00000        -0.55145
```

6.5.8 4Br

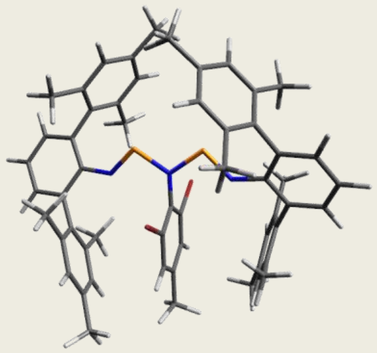

```
117
4Br @ PBE-D3/def2-TZVP
Br         -0.06362         0.74220        -2.87598
C           0.00949         1.68434        -1.22334
C           0.01024         0.96465        -0.01877
C           0.06697         3.07261        -1.25151
C           0.05318         1.71607         1.16445
H           0.07515         3.58766        -2.21163
C           0.11130         3.80209        -0.05896
Br          0.06660         0.81843         2.84311
C           0.09685         3.10609         1.15288
C           0.15431         5.30429        -0.08356
N          -0.01302        -0.44903        -0.00019
H           0.13803         3.64781         2.09696
H           0.80625         5.67054        -0.88894
H           0.52226         5.70733         0.86840
H          -0.85016         5.71836        -0.26122
P          -1.45964        -1.41048         0.00394
P           1.40962        -1.44681         0.01463
N          -2.57498        -0.29366         0.10228
N           2.55359        -0.36060        -0.09749
C          -3.94039        -0.40072         0.28640
C           3.91505        -0.50453        -0.28517
C          -4.56571        -1.53618         0.88044
C          -4.73582         0.71496        -0.08109
C           4.50558        -1.65731        -0.88192
C           4.74359         0.58820         0.07886
C          -5.93664        -1.51162         1.14056
C          -3.73253        -2.72693         1.19329
C          -6.11088         0.69204         0.17299
C          -4.12530         1.89581        -0.75412
C           5.87425        -1.66897        -1.15442
C           3.63978        -2.82775        -1.18295
C           6.11527         0.52850        -0.18768
C           4.17524         1.78091         0.76788
H          -6.39888        -2.38170         1.61179
C          -6.71310        -0.40462         0.79066
C          -2.98148        -2.77254         2.38964
C          -3.60980        -3.75996         0.24093
H          -6.71103         1.55564        -0.12105
C          -3.87661         1.86402        -2.14121
C          -3.84066         3.05870        -0.01319
C           6.68270        -0.58268        -0.81224
H           6.30929        -2.55159        -1.62822
C           3.49176        -3.84871        -0.22111
C           2.88628        -2.86493        -2.37802
H           6.74110         1.37453         0.10388
C           3.92605         1.73720         2.15523
C           3.93128         2.96420         0.04537
```

H	-7.78567	-0.40188	0.99024
C	-2.09169	-3.83278	2.59067
C	-3.10387	-1.67049	3.40529
C	-2.71665	-4.80834	0.48903
C	-4.38597	-3.70981	-1.04802
C	-3.38970	3.01342	-2.77100
C	-4.12062	0.61010	-2.93695
C	-3.36690	4.19135	-0.68429
C	-4.00269	3.07505	1.48331
H	7.75315	-0.60816	-1.02127
C	2.57160	-4.87592	-0.45870
C	4.27046	-3.80667	1.06656
C	1.96846	-3.90312	-2.56802
C	3.03972	-1.77793	-3.40551
C	3.48218	2.89351	2.80297
C	4.12169	0.46310	2.93207
C	3.49673	4.10272	0.73393
C	4.08870	2.99666	-1.45147
H	-1.50056	-3.85783	3.51030
C	-1.92952	-4.85058	1.64352
H	-2.52479	-1.90148	4.30870
H	-4.15245	-1.50352	3.69141
H	-2.73035	-0.71753	2.99797
H	-2.61854	-5.60462	-0.25422
H	-4.14026	-4.56724	-1.68779
H	-4.16512	-2.78710	-1.60772
H	-5.47114	-3.70909	-0.86865
H	-3.20554	2.98502	-3.84869
C	-3.14255	4.19429	-2.06340
H	-4.00630	0.79820	-4.01243
H	-5.12660	0.20343	-2.75700
H	-3.40013	-0.17346	-2.65299
H	-3.16873	5.09864	-0.10628
H	-3.34303	2.32577	1.94913
H	-5.02894	2.82711	1.79124
H	-3.74581	4.05991	1.89535
H	2.45413	-5.66275	0.29177
C	1.78167	-4.90794	-1.61163
H	4.01135	-4.65740	1.70999
H	4.06522	-2.87840	1.62319
H	5.35521	-3.82418	0.88585
H	1.37535	-3.92109	-3.48654
H	2.43898	-1.99256	-4.29873
H	4.09038	-1.65839	-3.70781
H	2.71304	-0.80633	-3.00268
H	3.29508	2.85428	3.87985
C	3.27733	4.09316	2.11328
H	4.02637	0.64180	4.01101
H	5.10664	0.01446	2.73653
H	3.36394	-0.28364	2.64564
H	3.31772	5.02269	0.17039
H	3.85547	3.99342	-1.84891
H	3.40960	2.27034	-1.92542
H	5.10766	2.72834	-1.76653
C	-0.90439	-5.93368	1.84411
C	-2.62310	5.41988	-2.76722
C	0.72882	-5.96618	-1.80131
C	2.81829	5.32923	2.84080
H	-1.15500	-6.83744	1.27210
H	-0.80902	-6.20865	2.90384

```
H           0.08620        -5.59070         1.50317
H          -2.75120         6.32106        -2.15186
H          -3.14054         5.58340        -3.72353
H          -1.54876         5.32627        -2.99640
H           0.62315        -6.24609        -2.85877
H          -0.25176        -5.59610        -1.45977
H           0.95847        -6.87194        -1.22368
H           2.74437         6.18938         2.16161
H           3.51295         5.59886         3.65081
H           1.83120         5.17937         3.30726
```

6.5.9 1Me

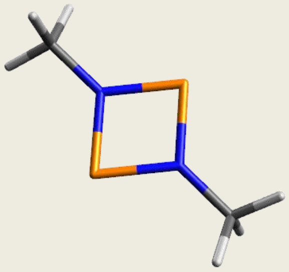

```
12
1Me @ PBE-D3/def2-TZVP
N           0.51299         0.30182        -0.36434
N           1.71058        -0.73382         1.18698
P           0.61019         0.57541         1.32703
P           1.61324        -1.00722        -0.50345
C           2.60648        -1.30198         2.17090
C          -0.38226         0.86759        -1.35000
H           0.07043         0.79737        -2.34946
H          -0.55713         1.93143        -1.13383
H          -1.35374         0.34962        -1.36937
H           2.80543        -2.35679         1.93277
H           2.14183        -1.26344         3.16660
H           3.56667        -0.76462         2.21163
```

6.5.10 2Me

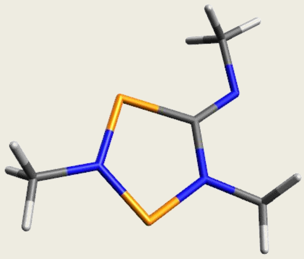

```
18
2Me @ PBE-D3/def2-TZVP
N           0.16423        -0.12961        -0.35541
N           1.94033        -0.92007         1.13196
P           0.57770        -1.81445         1.71200
P           1.80488         0.15092        -0.13999
C           3.25270        -1.09724         1.76374
C          -0.52582        -1.05253         0.47693
N          -1.78531        -1.24191         0.28197
C          -2.41109        -2.19596         1.17265
C          -0.59945         0.55756        -1.39284
H          -1.95250        -3.20389         1.09301
H          -3.47865        -2.29315         0.93361
H          -2.32827        -1.88950         2.23650
H           0.06411         1.23465        -1.94648
H          -1.42180         1.12191        -0.93271
H          -1.04448        -0.18037        -2.07398
H           3.57399        -2.14428         1.67863
H           3.19929        -0.82591         2.82688
H           3.99491        -0.45509         1.26855
```

6.5.11 3Me

```
25
3Me @ PBE-D3/def2-TZVP
```

```
C          -0.23201          0.21648          0.23161
N          -1.31416          0.88465          0.26093
N           0.57292          0.12351         -0.90126
N           2.21958         -0.09900          1.00780
P           0.67886         -0.78980          1.59299
P           2.00992         -0.77740         -0.59127
C           3.42984         -0.43364          1.76803
C          -2.11449          0.89585          1.46518
C           0.24444          0.82253         -2.13152
N           0.19915         -2.83802         -0.20348
N           1.14081         -2.05330          0.35547
N          -0.88577         -2.78487          0.43299
C          -1.90780         -3.68744         -0.07486
H          -1.58323         -4.20981         -0.98774
H          -2.81846         -3.10258         -0.27205
H          -2.15421         -4.42169          0.70771
H           1.00080          0.58258         -2.88948
H           0.22099          1.91103         -1.96990
H          -0.74689          0.51204         -2.49211
H          -3.11413          0.49010          1.24329
H          -2.25912          1.93394          1.80301
H          -1.67841          0.30979          2.29686
H           4.30895         -0.04920          1.23080
H           3.57033         -1.51614          1.93870
H           3.38689          0.06716          2.74547
```

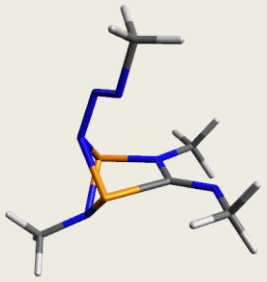

6.5.12 MeNC

```
6
MeNC @ PBE-D3/def2-TZVP
C          -3.03490         -1.31913         -0.53279
N          -2.30242         -1.86819          0.20726
C          -1.41971         -2.52189          1.10245
H          -0.39446         -2.48706          0.70964
H          -1.71983         -3.57134          1.22558
H          -1.44437         -2.02555          2.08206
```

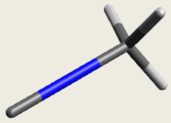

6.5.13 MeN_3

```
7
MeN3 @ PBE-D3/def2-TZVP
N          -1.04217         -2.41419         -0.78921
N          -0.17350         -2.30588         -1.52952
N          -1.99351         -2.67053         -0.05247
C          -2.43099         -1.60094          0.85579
H          -3.29127         -1.98945          1.40994
H          -2.74132         -0.70023          0.30267
H          -1.63948         -1.33014          1.57306
```

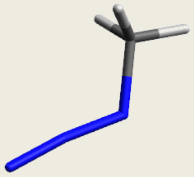

6.5.14 VDW1

```
18
VDW1 @ PBE-D3/def2-TZVP
N           0.48117          0.27157         -0.35802
N           1.70929         -0.73830          1.19046
```

```
P           0.59162         0.55522         1.32706
P           1.60077        -1.01925        -0.50218
C           2.61348        -1.29741         2.17212
C          -3.01908        -1.31688        -0.53883
N          -2.31173        -1.87359         0.21801
C          -1.42262        -2.52525         1.10784
C          -0.38504         0.87230        -1.35489
H          -0.40042        -2.47745         0.69642
H          -1.71882        -3.57617         1.22712
H          -1.44301        -2.02382         2.08363
H           0.08730         0.80025        -2.34455
H          -0.53285         1.93701        -1.12588
H          -1.36675         0.37642        -1.39178
H           2.82008        -2.35159         1.93710
H           2.15550        -1.25729         3.17095
H           3.57012        -0.75355         2.20506
```

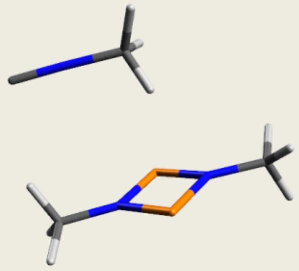

6.5.15 VDW2

```
18
VDW2 @ PBE-D3/def2-TZVP
N           0.69578         0.39325         0.19069
N           1.88231        -1.15024         1.25313
P           1.23457         0.33366         1.84277
P           1.34407        -1.08048        -0.36712
C           2.68395        -2.20178         1.84153
C           2.24835        -0.20387         4.22662
N           2.18010         0.42429         5.22000
C           2.04467         1.24512         6.36405
C          -0.11618         1.39302        -0.46130
H           3.02659         1.63535         6.66503
H           1.62091         0.66526         7.19542
H           1.37729         2.08878         6.13818
H           0.35280         1.74164        -1.39435
H          -0.22626         2.26071         0.20588
H          -1.12309         1.01350        -0.69712
H           2.16536        -2.67484         2.68648
H           3.64716        -1.81708         2.20361
H           2.87594        -2.96637         1.07264
```

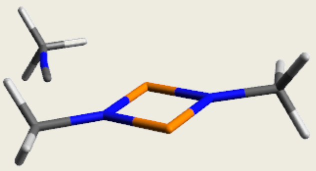

6.5.16 VDW3

```
25
VDW3 @ PBE-D3/def2-TZVP
C          -0.09338         1.02889         0.47888
N          -1.17253         1.73098         0.37843
N           0.63921         0.72995        -0.70000
N           1.92925        -0.43394         1.02168
P           0.67310         0.30617         1.96324
P           1.99647        -0.25064        -0.63115
C           2.95000        -1.25207         1.68558
C          -1.83251         2.02568         1.63294
C           0.13005         1.22997        -1.97442
N          -1.03872        -2.41113        -0.79006
N          -0.18166        -2.31521        -1.55122
N          -1.97244        -2.66453        -0.03770
C          -2.43828        -1.60034         0.86185
```

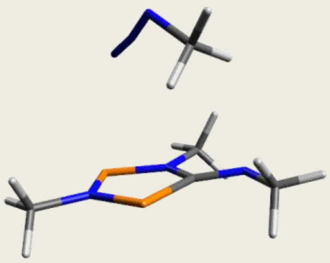

```
H          -3.30501       -1.99746        1.39912
H          -2.72837       -0.69679        0.30415
H          -1.64776       -1.32589        1.58412
H           0.81621        0.93718       -2.77933
H           0.03669        2.32287       -1.92751
H          -0.86819        0.81405       -2.16632
H          -2.77413        2.56006        1.44856
H          -1.20535        2.66032        2.29123
H          -2.07010        1.10761        2.21342
H           3.66907       -1.62597        0.94316
H           2.47988       -2.10897        2.18730
H           3.48702       -0.65150        2.43237
```

6.5.17 INT1

```
18
INT1 @ PBE-D3/def2-TZVP
N           0.53880        0.73498       -0.46377
N           2.00753       -0.34184        0.91392
P           0.36302        0.23296        1.22368
P           1.42513       -0.77835       -0.69905
C           2.63604       -1.33561        1.77761
C          -0.04640       -1.48063        0.32246
N          -0.78613       -2.49017        0.32755
C          -1.83037       -2.63471        1.33070
C          -0.62562        1.05711       -1.28166
H          -1.66173       -3.56563        1.89202
H          -2.80175       -2.73697        0.82463
H          -1.87418       -1.78526        2.03693
H          -0.29666        1.24988       -2.31333
H          -1.09046        1.97727       -0.89819
H          -1.39417        0.26189       -1.30576
H           2.04127       -2.25882        1.91031
H           2.81240       -0.89109        2.76812
H           3.61227       -1.61276        1.35346
```

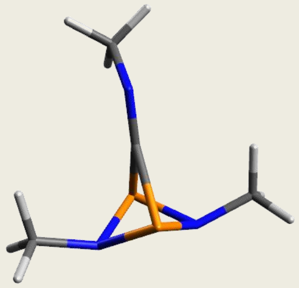

6.5.18 INT2

```
18
INT2 @ PBE-D3/def2-TZVP
N          -1.10787        0.63964       -2.50935
N          -0.05428       -0.64809       -0.59046
P          -0.72472        0.70799        0.31589
P          -0.29542       -0.68727       -2.26196
C           0.68678       -1.73705        0.03365
C          -0.16618        0.27718        1.84275
N           0.06287        0.10417        3.03089
C           1.21485        0.57176        3.78445
C          -1.58393        1.08981       -3.79571
H           1.90765        1.16762        3.16955
H           1.73979       -0.30006        4.19996
H           0.85723        1.17239        4.63236
H          -1.33333        0.42632       -4.64520
H          -1.17272        2.08924       -4.01399
H          -2.68029        1.20273       -3.76659
H           0.06904       -2.25486        0.78296
H           1.59532       -1.36026        0.53065
```

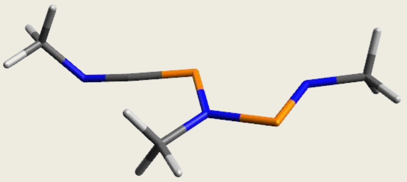

```
H           0.98518        -2.46125        -0.73985
```

6.5.19 INT3

```
25
INT3 @ PBE-D3/def2-TZVP
C           0.20238         1.05550        -0.43563
N          -0.82400         1.81957        -0.49421
N           1.21759         1.24573        -1.41694
N           2.29242        -0.34803         0.12796
P           0.64684        -0.29236         0.77048
P           2.69018         0.49120        -1.23133
C           3.21056        -1.34281         0.68473
C          -1.83933         1.71225         0.52639
C           0.98987         2.21685        -2.48674
N          -0.92175        -2.25076         0.37425
N           0.25175        -1.89480         0.03325
N          -1.72260        -1.45417         0.97085
C          -2.98853        -2.03770         1.39327
H          -3.12304        -3.04867         0.97904
H          -3.82137        -1.39683         1.06342
H          -3.03219        -2.09718         2.49367
H           1.87640         2.25659        -3.13294
H           0.78665         3.20544        -2.05521
H           0.11131         1.91552        -3.07155
H          -2.64192         2.43282         0.32402
H          -1.42541         1.91789         1.53109
H          -2.24979         0.68371         0.57353
H           4.17627        -1.30601         0.16060
H           2.76456        -2.34060         0.56528
H           3.37312        -1.14316         1.75276
```

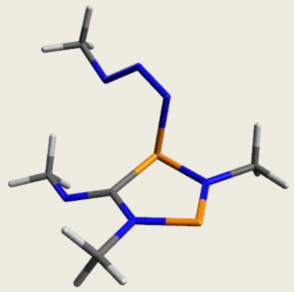

6.5.20 TS1

```
18
TS1 @ PBE-D3/def2-TZVP
N           0.47645         1.79589        -1.14032
N           1.66037         0.60102         0.33901
P           0.31540         1.64597         0.56408
P           1.47329         0.41872        -1.35398
C           2.27969        -0.29645         1.28357
C          -0.68940        -0.53840        -0.25987
N          -1.21747        -1.53059         0.14062
C          -2.01027        -2.31762         1.01790
C          -0.54568         2.25633        -2.05285
H          -1.45870        -3.21822         1.32268
H          -2.92563        -2.64112         0.50250
H          -2.28957        -1.74539         1.91735
H          -0.12083         2.37360        -3.06074
H          -0.92083         3.23829        -1.72843
H          -1.39279         1.54992        -2.10447
H           1.65778        -1.18735         1.47907
H           2.45393         0.22548         2.23609
H           3.25427        -0.63009         0.89780
```

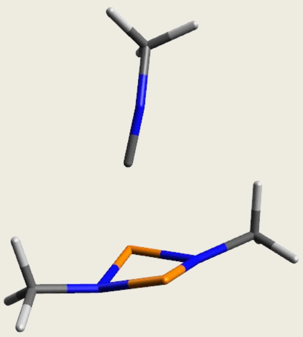

6.5.21 TS2

```
18
TS2 @ PBE-D3/def2-TZVP
N         0.07982      1.04942     -0.83962
N         1.97229      0.46072      0.36187
P         0.51017      1.22591      0.89677
P         1.21548     -0.33653     -0.97962
C         2.90469     -0.20259      1.26416
C        -0.52060     -0.27992      0.03978
N        -1.74015     -0.56651     -0.18137
C        -2.35336     -1.68361      0.50746
C        -0.82007      1.72782     -1.74317
H        -1.84540     -2.64228      0.28798
H        -3.39999     -1.77865      0.18732
H        -2.34790     -1.55340      1.60667
H        -0.42972      1.67689     -2.76758
H        -0.92251      2.78008     -1.44887
H        -1.80327      1.22052     -1.68681
H         2.45563     -1.05248      1.80590
H         3.27772      0.52464      2.00012
H         3.76716     -0.57002      0.68901
```

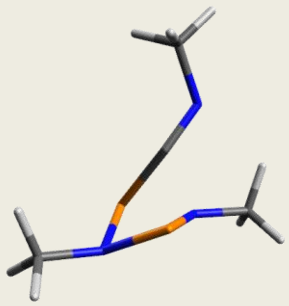

6.5.22 TS3

```
18
TS3 @ PBE-D3/def2-TZVP
N        -0.85795      0.32937     -2.22648
N         0.32879     -1.19427     -1.13257
P        -0.30963      0.30590     -0.55498
P        -0.21229     -1.14875     -2.74706
C         1.11129     -2.25541     -0.53702
C         0.61450     -0.17956      1.60345
N         0.60209      0.37811      2.64276
C         0.50685      1.14626      3.82538
C        -1.63795      1.33783     -2.90172
H         1.44319      1.69685      3.99301
H         0.32035      0.49173      4.68809
H        -0.32014      1.86559      3.73621
H        -1.10388      1.74978     -3.77239
H        -1.83390      2.16459     -2.20240
H        -2.60857      0.94387     -3.24232
H         0.58261     -2.72103      0.30627
H         2.08037     -1.88596     -0.17411
H         1.29427     -3.02488     -1.30412
```

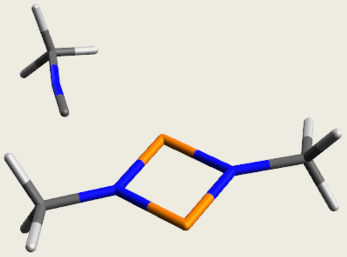

6.5.23 TS4

```
18
TS4 @ PBE-D3/def2-TZVP
N        -1.19461      0.55348     -2.54347
N         0.10909     -0.61030     -0.75042
P        -0.47832      0.85055      0.07471
P        -0.34586     -0.76807     -2.35875
C         0.93451     -1.63324     -0.13044
C         0.23808      0.51213      1.59729
```

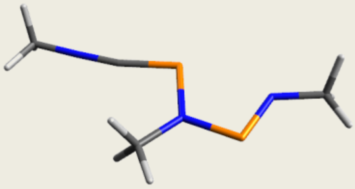

```
N           0.63192         0.49067         2.72240
C           1.06940         0.51394         4.05398
C          -1.85699         0.96818        -3.75530
H           1.67264        -0.38432         4.25551
H           0.21714         0.51705         4.75441
H           1.69791         1.39863         4.25218
H          -1.73765         0.27488        -4.60979
H          -1.48048         1.95613        -4.06980
H          -2.93759         1.08928        -3.57005
H           0.41965        -2.09396         0.72820
H           1.89201        -1.21427         0.21919
H           1.14915        -2.42075        -0.86985
```

6.5.24 TS5

```
25
TS5 @ PBE-D3/def2-TZVP
C           0.31047         1.15817        -0.32112
N          -0.75146         1.88202        -0.33643
N           1.14651         1.14061        -1.46834
N           2.41366        -0.24185         0.12152
P           0.95668         0.06400         0.99699
P           2.58945         0.29421        -1.44437
C           3.37568        -1.20096         0.67101
C          -1.56958         1.87005         0.85411
C           0.74041         1.92568        -2.63183
N          -0.97710        -2.01054         0.51967
N           0.17483        -1.85367         0.23813
N          -1.93858        -1.43830         1.10323
C          -3.20449        -2.17062         1.11021
H          -3.57752        -2.36866         0.09134
H          -3.93564        -1.53984         1.62974
H          -3.12820        -3.12926         1.65015
H           1.48941         1.81191        -3.42615
H           0.64222         2.98259        -2.35050
H          -0.24048         1.57900        -2.98278
H          -2.36732         2.61928         0.76691
H          -0.98033         2.09251         1.76728
H          -2.02708         0.86876         1.01295
H           4.24051        -1.29163        -0.00136
H           2.89439        -2.18399         0.77434
H           3.72354        -0.85945         1.65529
```

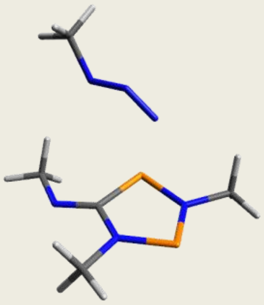

6.5.25 TS6

```
25
TS6 @ PBE-D3/def2-TZVP
C           0.05158         0.95352        -0.24107
N          -0.85955         1.84220        -0.18701
N           0.88356         0.89465        -1.38701
N           2.31895        -0.18521         0.34559
P           0.51383        -0.43591         0.93685
P           2.25299        -0.06136        -1.28093
C           3.29591        -1.07498         0.97028
C          -1.71994         1.87479         0.97453
C           0.53727         1.65196        -2.58530
N          -0.69780        -2.17146        -0.27825
```

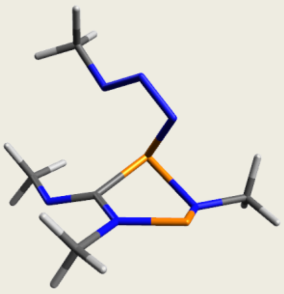

N	0.53916	-1.79672	-0.27924
N	-1.38773	-1.33761	0.45907
C	-2.69466	-1.78088	0.90493
H	-3.06929	-2.56713	0.23198
H	-3.39467	-0.93339	0.90704
H	-2.65345	-2.19111	1.92876
H	1.26420	1.42165	-3.37501
H	0.55489	2.72892	-2.37012
H	-0.47441	1.38339	-2.91687
H	-1.83885	2.91698	1.30631
H	-1.35038	1.26675	1.82139
H	-2.72142	1.51095	0.69406
H	4.24448	-1.07482	0.41269
H	2.91471	-2.10809	1.01301
H	3.49062	-0.72711	1.99434

6.5.26 2H

9
2H @ PBE-D3/def2-TZVP

C	0.00000	1.15501	0.00000
N	-0.15732	2.43603	0.00000
N	-1.16117	0.33760	0.00000
N	0.55239	-1.34012	-0.00000
P	1.49338	0.10709	-0.00000
P	-1.11813	-1.34048	0.00000
H	1.03355	-2.23848	-0.00000
H	0.74371	2.92225	-0.00000
H	-2.04325	0.85237	0.00000

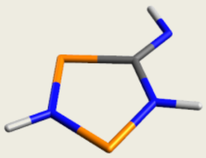

6.5.27 HN_3

4
HN_3 @ PBE-D3/def2-TZVP

N	0.00000	0.10856	0.00000
N	-0.29129	1.21232	0.00000
N	0.13083	-1.12305	0.00000
H	1.12318	-1.38479	0.00000

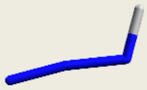

7 References

[1] H. Braunschweig, F. Hupp, I. Krummenacher, L. Mailänder, F. Rauch, *Chem. Eur. J.* **2015**, *21*, 17844–17849.

[2] A. A. Ageshina, G. A. Chesnokov, M. A. Topchiy, I. V. Alabugin, M. S. Nechaev, A. F. Asachenko, *Org. Biomol. Chem.* **2019**, *17*, 4523–4534.

[3] K. Tanaka, A. R. Pradipta (RIKEN), *EP4233916A1*, **2023**.

[4] B. L. Small, R. Rios, E. R. Fernandez, D. L. Gerlach, J. A. Halfen, M. J. Carney, *Organometallics* **2010**, *29*, 6723–6731.

[5] S. Wu, J. Huang, S. Gazzarrini, S. He, L. Chen, J. Li, L. Xing, C. Li, L. Chen, C. G. Neochoritis, G. P. Liao, H. Zhou, A. Dömling, A. Moroni, W. Wang, *ChemMedChem* **2015**, *10*, 1837–1845.

[6] Y. Pilopp, J. Bresien, D. T. Gschwind, A. Villinger, D. Michalik, A. Schulz, *Chem. Eur. J.* **2023**, *29*, e202300764.

[7] F. Reiß, A. Schulz, A. Villinger, N. Weding, *Dalton Trans.* **2010**, *39*, 9962.

[8] E. Zander, J. Bresien, V. V. Zhivonitko, J. Fessler, A. Villinger, D. Michalik, A. Schulz, *J. Am. Chem. Soc.* **2023**, *145*, 14484–14497.

[9] J. Bresien, T. Kröger-Badge, S. Lochbrunner, D. Michalik, H. Müller, A. Schulz, E. Zander, *Chem. Sci.* **2019**, *10*, 3486–3493.

[10] G. M. Sheldrick, *Acta Crystallogr. Sect. A Found. Adv.* **2015**, *71*, 3–8.

[11] G. M. Sheldrick, *Acta Crystallogr. Sect. C Struct. Chem.* **2015**, *71*, 3–8.

[12] G. M. Sheldrick, *SADABS Version 2*, University of Göttingen, Germany, **2004**.

[13] *Gaussian 09, Revision E.01*, M. J. Frisch, G. W. Trucks, H. B. Schlegel, G. E. Scuseria, M. A. Robb, J. R. Cheeseman, G. Scalmani, V. Barone, B. Mennucci, G. A. Petersson, H. Nakatsuji, M. Caricato, X. Li, H. P. Hratchian, A. F. Izmaylov, J. Bloino, G. Zheng, J. L. Sonnenberg, M. Hada, M. Ehara, K. Toyota, R. Fukuda, J. Hasegawa, M. Ishida, T. Nakajima, Y. Honda, O. Kitao, H. Nakai, T. Vreven, J. A. Montgomery Jr., J. E. Peralta, F. Ogliaro, M. Bearpark, J. J. Heyd, E. Brothers, K. N. Kudin, V. N. Staroverov, T. Keith, R. Kobayashi, J. Normand, K. Raghavachari, A. Rendell, J. C. Burant, S. S. Iyengar, J. Tomasi, M. Cossi, N. Rega, J. M. Millam, M. Klene, J. E. Knox, J. B. Cross, V. Bakken, C. Adamo, J. Jaramillo, R. Gomperts, R. E. Stratmann, O. Yazyev, A. J. Austin, R. Cammi, C. Pomelli, J. W. Ochterski, R. L. Martin, K. Morokuma, V. G. Zakrzewski, G. A. Voth, P. Salvador, J. J. Dannenberg, S. Dapprich, A. D. Daniels, O. Farkas, J. B. Foresman, J. V. Ortiz, J. Cioslowski, D. J. Fox, Gaussian, Inc., Wallingford CT, **2013**.

[14] F. Neese, *The ORCA program system*, Wiley Interdiscip. Rev.: Comput. Mol. Sci., **2012**, *2*, 1, 73–78.

[15] E. D. Glendening, J. K. Badenhoop, A. E. Reed, J. E. Carpenter, J. A. Bohmann, C.

M. Morales, C. R. Landis, F. Weinhold, *NBO 6.0*, Theoretical Chemistry Institute, University of Wisconsin, Madison, **2013**.

[16] J. E. Carpenter, F. Weinhold, *J. Mol. Struct. THEOCHEM* **1988**, *169*, 41–62.

[17] F. Weinhold, J. E. Carpenter, in *The Structure of Small Molecules and Ions* (Eds.: R. Naaman, Z. Vager), Springer, Boston, MA, **1988**, pp. 227–236.

[18] F. Weinhold, C. R. Landis, *Valency and Bonding. A Natural Bond Orbital Donor-Acceptor Perspective*, Cambridge University Press, **2005**.

[19] J. P. Perdew, K. Burke, M. Ernzerhof, *Phys. Rev. Lett.* **1996**, *77*, 3865–3868.

[20] J. P. Perdew, K. Burke, M. Ernzerhof, *Phys. Rev. Lett.* **1997**, *78*, 1396–1396.

[21] C. Adamo, V. Barone, *J. Chem. Phys.* **1999**, *110*, 6158–6170.

[22] S. Grimme, J. Antony, S. Ehrlich, H. Krieg, *J. Chem. Phys.* **2010**, *132*, 154104.

[23] S. Grimme, S. Ehrlich, L. Goerigk, *J. Comput. Chem.* **2011**, *32*, 1456–1465.

[24] F. Weigend, R. Ahlrichs, *Phys. Chem. Chem. Phys.* **2005**, *7*, 3297.

[25] F. Weigend, *Phys. Chem. Chem. Phys.* **2006**, *8*, 1057–1065.

[26] F. London, *J. Phys. le Radium* **1937**, *8*, 397–409.

[27] R. McWeeny, *Phys. Rev.* **1962**, *126*, 1028–1034.

[28] R. Ditchfield, *Mol. Phys.* **1974**, *27*, 789–807.

[29] K. Wolinski, J. F. Hinton, P. Pulay, *J. Am. Chem. Soc.* **1990**, *112*, 8251–8260.

[30] J. R. Cheeseman, G. W. Trucks, T. A. Keith, M. J. Frisch, *J. Chem. Phys.* **1996**, *104*, 5497–5509.

[31] A. Hellweg, C. Hättig, S. Höfener, W. Klopper, *Theor. Chem. Acc.* **2007**, *117*, 587–597.

[32] C. J. Jameson, A. De Dios, A. Keith Jameson, *Chem. Phys. Lett.* **1990**, *167*, 575–582.

[33] C. van Wüllen, *Phys. Chem. Chem. Phys.* **2000**, *2*, 2137–2144.

[34] W. Deng, J. R. Cheeseman, M. J. Frisch, *J. Chem. Theory Comput.* **2006**, *2*, 1028–1037.

[35] C. Riplinger, F. Neese, *J. Chem. Phys.* **2013**, *138*, 034106.

[36] D. G. Liakos, Y. Guo, F. Neese, *J. Phys. Chem. A* **2020**, *124*, 90–100.

[37] D. G. Liakos, M. Sparta, M. K. Kesharwani, J. M. L. Martin, F. Neese, *J. Chem. Theory Comput.* **2015**, *11*, 1525–1539.

[38] C. Riplinger, P. Pinski, U. Becker, E. F. Valeev, F. Neese, *J. Chem. Phys.* **2016**, *144*, 024109.

[39] Christopher J. Cramer, *Essentials of Computational Chemistry: Theories and Models*, John Wiley & Sons, Ltd, Chichester, UK, **2004**.

[40] G. Mills, H. Jónsson, G. K. Schenter, *Surf. Sci.* **1995**, *324*, 305–337.

[41] H. Jónsson, G. Mills, K. W. Jacobsen, in *Classical and Quantum Dynamics in Condensed Phase Simulations* (Eds.: B. J. Berne, G. Ciccotti, D. F. Coker), World Scientific, Singapore, **1998**, pp. 385–404.

[42] G. Henkelman, H. Jónsson, *J. Chem. Phys.* **2000**, *113*, 9978–9985.

[43] G. Henkelman, B. P. Uberuaga, H. Jónsson, *J. Chem. Phys.* **2000**, *113*, 9901–9904.

[44] E. Maras, O. Trushin, A. Stukowski, T. Ala-Nissila, H. Jónsson, *Comput. Phys. Commun.* **2016**, *205*, 13–21.

[45] V. Ásgeirsson, B. O. Birgisson, R. Bjornsson, U. Becker, F. Neese, C. Riplinger, H. Jónsson, *J. Chem. Theory Comput.* **2021**, *17*, 4929–4945.
